# Bacterial Community Structure in the Asian Rice Gall Midge Reveals a Varied Microbiome Rich in Proteobacteria

**DOI:** 10.1038/s41598-017-09791-0

**Published:** 2017-08-25

**Authors:** Abhishek Ojha, Deepak Kumar Sinha, A. P. Padmakumari, J. S. Bentur, Suresh Nair

**Affiliations:** 1 International Centre for Genetic Engineering and Biotechnology (ICGEB), Aruna Asaf Ali Marg, New Delhi, 110 067 India; 2 Indian Institute of Rice Research, Rajendranagar, Hyderabad, 500030 India; 3 Agri Biotech Foundation, Rajendranagar, Hyderabad, 500 030 India; 4 Present Address: State Key Laboratory of Biocontrol, School of Life Sciences, Sun Yat-sen University, Guangzhou, 510275 China

## Abstract

The Asian rice gall midge (ARGM) has emerged as a model gall forming pest of rice. The ARGM infestation of rice results in failure of panicle formation and economic loss. Understanding the molecular basis of ARGM-rice interactions is very crucial in order to control this devastating pest of rice. The current investigation was devised to identify bacterial communities present in the ARGM and in addition the bacterial diversity in the maggots during their interaction with susceptible or resistant rice varieties. Sequencing of 16S rRNA bacterial gene (V3-V4 region) revealed differences in the microflora of the ARGM maggots feeding on susceptible or resistant rice hosts. Results revealed that *Wolbachia* was the predominant bacterium in pupae and adults while *Pseudomonas* was predominant in maggots. Further, we observed that members of proteobacteria were predominant across all the samples. There was high species diversity in maggots isolated from susceptible rice and a high representation of unclassified bacteria in maggots isolated from resistant rice. This is the first study that reports variation of microbiome of the ARGM, based on host phenotype from which it was isolated, and results suggest that these variations could have an important role in host’s susceptibility.

## Introduction

The Asian rice gall midge (ARGM) is one of the most destructive insect pests of rice^1^. As a member of the specialized gall making insect family Cecidomyiidae, it spends most part of its life cycle within the rice plant. Maggot hatching from egg is a tiny larva that twists and slides down the space between leaf sheaths facilitated by a thin film of water and moisture. Upon reaching the apical meristem or crown tissue, maggot initiates feeding by lacerating the tissue with its sternal spatula, secreting saliva and engulfing the oozing cell contents. This results in noticeable change in growth of surrounding tissue that encircles the maggot to form a gall chamber and form nutritive nurse cells (i.e. shifting of the metabolic process from a reproductive phase to a vegetative phase)^1^. Maggot molts twice and passes through three instars prior to pupation within the now tubular gall. Pupa is exceptionally active and moves upwards in the gall through wriggling movement, drills an exit hole at the tip of gall using its cephalic horns and partially protrudes out to facilitate adult emergence. Adult fly resembling mosquito with pink body has short life span of 1–2 days. Adults do not feed on rice but mate and lay eggs on plant within the short life span. Successful establishment of the insect in the plant and formation of gall renders that tiller sterile without further formation of panicles and grains that result in economic loss. However, several of the rice genotypes resist insect establishment and gall formation offering genetic host-plant resistance^1^. Gall flies lay eggs almost indiscreetly with reference to such genotypes and egg hatching or movement of hatched maggots into the plant to reach apical meristem is not hindered. However, maggot survival is dramatically reduced to nil within 48 h of feeding on resistant genotypes. Resistance in rice against the ARGM is often accompanied by tissue necrosis referred as hypersensitive response (HR+) and in few other cases where resistance is without accompanying necrosis (HR−)^2^. On the contrary, in a susceptible plant, maggots lacerate and feed at the meristematic region of the rice seedling resulting in formation of an enclosed tube like structure referred to as gall. Gall formation renders rice tillers sterile i.e. shifts the developmental process from a reproductive phase to a vegetative phase^3^. Various studies have described and defined the mechanism involved during rice defense^1^ and the ARGM counter defense^4^ but no study has reported the involvement of microbes in the ARGM-rice interaction. Moreover, insect associated microbiome is known to be involved in a three-way interaction between plant-insect-microbe^5^ and therefore, information regarding identity and diversity of bacterial community composition within the ARGM from both susceptible and resistant rice hosts is likely to contribute towards a better understanding of ARGM biology and gall midge-rice interactions as well. Here, we investigate and evaluate the ARGM-associated microbiome in an effort to understand this interaction and derive meaningful insights into this interaction to evolve novel strategies to control this important insect pest of rice.

We analysed the bacterial community composition in the Asian rice gall midge (ARGM) biotype 1 using a Next Generation Sequencing (NGS) protocol with Illumina-MiSeq platform with focus on the V3-V4 hypervariable region of 16S rRNA bacterial gene. We demonstrated the presence of specific bacterial community structure within each sample of the ARGM (isolated at different developmental stages and from resistant and susceptible rice host) and further showed that the bacterial community structure differed among the samples with reference to the developmental stages and the host. Furthermore, these observed variations of the microbiome of the ARGM, with reference to the host from which they were isolated, may have an influential impact on the nature of the interaction between ARGM and rice.

## Results

### PCR amplification of 16S V3-V4 hypervariable region from the Asian rice gall midge samples

**Figure 1:**
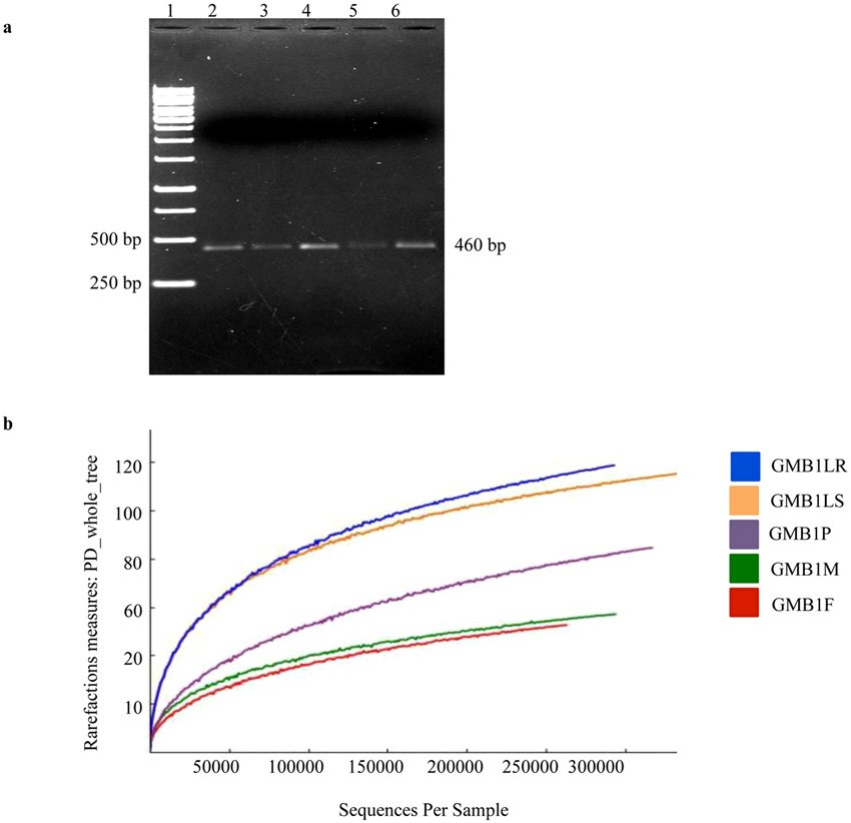
(**a**) PCR amplification of hypervariable region (V3-V4) of the Asian rice gall midge biotype 1 (GMB1). 1, 1 kb DNA ladder, 2, GMB1M (adult male), 3, GMB1F (adult female), 4, GMB1P (pupa), 5, GMB1LS (maggots from susceptible host) and 6, GMB1LR (maggots from resistant host). (**b**) Comparative rarefaction curve analyses for observed microbial OTUs. The rarefaction curves of microbial community in GMB1 samples; GMB1F (red), GMB1LS (dark yellow), GMB1LR (blue), GMB1M (green) and GMB1P (violet). (Multiple exposure gels are presented in Supplementary Figure 1).

A 460 bp fragment of the hypervariable V3-V4 region of the 16S rRNA was PCR amplified using specific universal primers (see Material and Methods section) from total genomic DNAs (OD_260/280_ ratio = 1.80–1.91 and OD_260/230_ ratio = 1.45–1.85) of the Asian rice gall midge biotype 1 (GMB1) adults (male and female), pupae and maggots isolated from TN1 and RP (Fig. 1a).

### Analysis of Illumina-MiSeq raw reads

Libraries (of the V3-V4 region) prepared from the 460 bp PCR-amplified fragment (OD_260/280_ ratio = 1.75–1.92 and OD_260/230_ ratio = 1.50–1.82) from the different GMB1 samples (Supplementary Table S1) yielded total of 4.3 million raw reads on the Illumina-MiSeq sequencing platform.

Pre-processing of the raw reads for elimination of the primer containing sequences, possible adapter sequences, low quality reads and unique dual index barcodes sequences containing 5**–**7 nucleotides, yielded 4.1 million high quality reads. These high quality reads ranged from 94.0**–**96.1% across the GMB1 samples (Supplementary Table S2). Duplicate and chimeric reads were removed using CD-HIT-DUP tool to block overestimated abundance of species, genes and or function. The non-overlapping reads were retained for further analysis (Supplementary Table S2).

### Identification, assignment of operational taxonomic units (OTUs)

**Figure 2:**
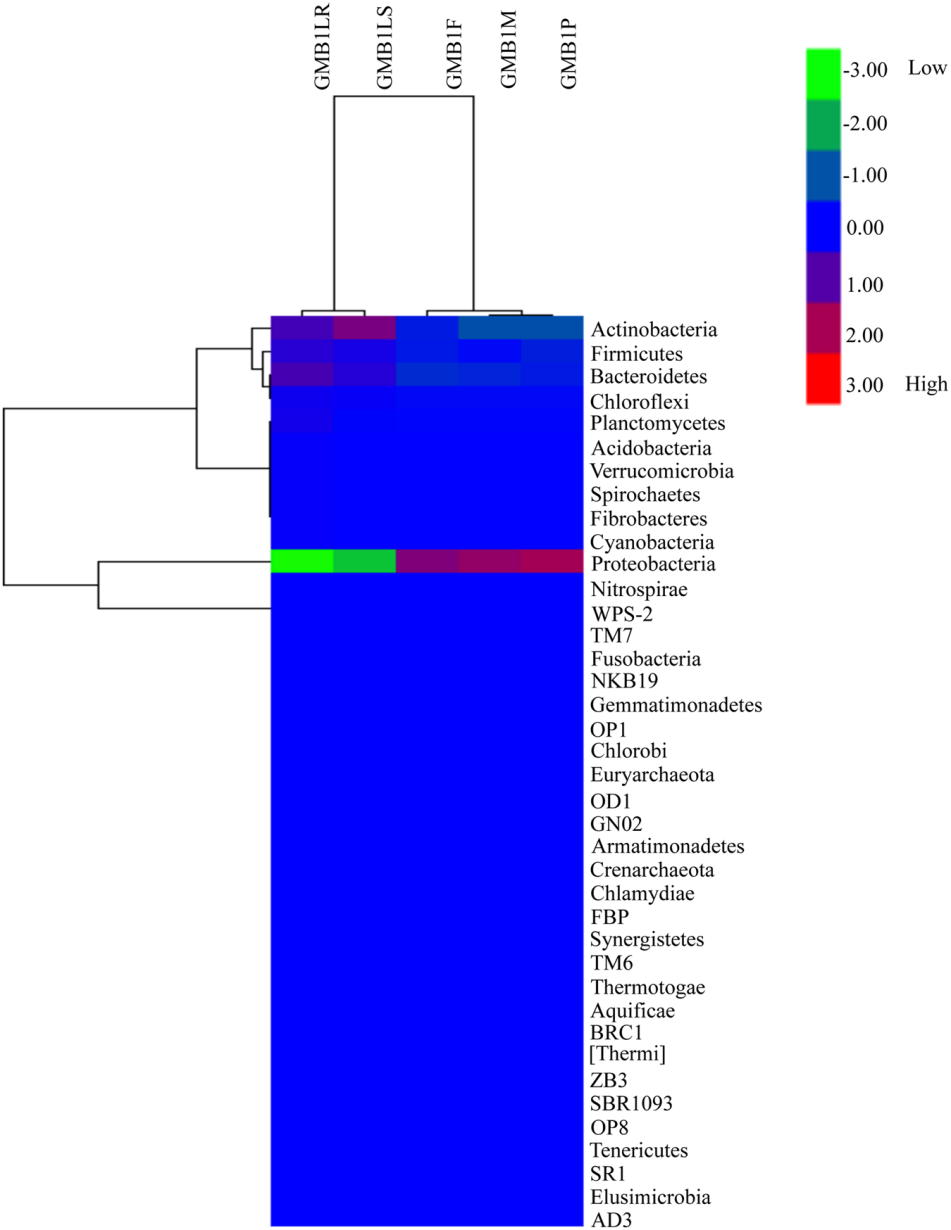
Heat map showing relative distribution and abundance of the OTUs among the GMB1 samples at phylum level.

All sequences from GMB1 samples were clustered into OTUs at 97% sequence similarity using QIIME (see ref. 43). A total of 7609 OTUs were identified that represented the bacterial species present in these samples (Table 1, Supplementary Data S1) at a confidence limit of 0.8 for assignment of each representative sequences. These assignments contained both specific bacterial species as well as non-assignable species that were labelled as unassigned OTUs. For all GMB1 samples, the rarefaction curves reached near plateau, indicating that the sampling depth and sequencing coverage were good (Fig. 1b). Maggots from susceptible hosts (GMB1LS) showed highest number (2280) of OTUs followed by maggots from resistant hosts (GMB1LR) with 2161 OTUs, pupae (GMB1P) with 1270 OTUs, adult male (GMB1M) with 992 OTUs and adult female (GMB1F) with 906 OTUs (Table 1, Supplementary Data S1). A heat map image of OTUs was generated using QIIME pipeline and results clearly illustrated the phylum-level microbial abundance and differences in the taxonomic composition among the GMB1 samples (GMB1F, GMB1M, GMB1P, GMB1LS and GMB1LR) (Fig. 2). An interactive graphical overview of relative abundance of bacterial diversity and confidence limits within the complex hierarchies of OTUs classifications from phylum up to species level was obtained through the Krona chart (Supplementary Data S2).

**Table 1:** Alpha-diversity indices of microbial community structure in the GMB1 samples.

S. No.	Sample name	Shannon	Simpson	Chao1	PD_whole_tree	Observed species
1.	GMB1F	1.89	0.59	1604	51.92	906
2.	GMB1M	2.51	0.67	1620	56.39	992
3.	GMB1P	1.48	0.54	2321	82.78	1270
4.	GMB1LS	4.63	0.84	3247	112.3	2280
5.	GMB1LR	4.68	0.82	3140	115.3	2161
	Total	15.19	3.46	11932	418.69	7609

The diversity indices (Shannon and Simpson index) and species richness indices (Chao1, PD (phylogenetic diversity)_whole_tree and observed OTUs) were determined at 97% sequence similarity from each GMB1 sample. Maggot (GMB1LR and GMB1LS) samples showed highest alpha diversity among the GMB1 samples. GMB1F, rice gall midge biotype 1 female (adult); GMB1M, rice gall midge biotype 1 male (adult); GMB1P, rice gall midge biotype 1 pupae; GMB1LS, rice gall midge biotype 1 larvae isolated from susceptible host; GMB1LR, rice gall midge biotype 1 larvae isolated from resistant host.

### Diversity Index

The diversity of microbial community structure in the GMB1 samples was estimated by alpha and beta diversity indices. We used 5 groups of alpha diversity (diversity within sample) indices to compare the species richness estimators (observed OTUs, Chao1 and PD_whole_tree) and diversity index (Shannon and Simpson index) of the microbiome from each GMB1 sample and these index values were significantly different between the samples (Table 1). Shannon information index of diversity ranged from 1.48 to 4.68, with an average of 3.04 and the observed Simpson index of diversity ranged from 0.54 to 0.84, with an average of 0.69 (Table 1). The Chao1 species richness estimator value revealed that OTUs richness varied from 1604 to 3247 with the highest value in GMB1LS among the GMB1 samples. In addition, the rarefaction curves also revealed the same pattern of the highest relative diversity being present in the GMB1LR. Significantly, more OTUs were observed in GMB1LS followed by GMB1LR (Table 1).

**Figure 3:**
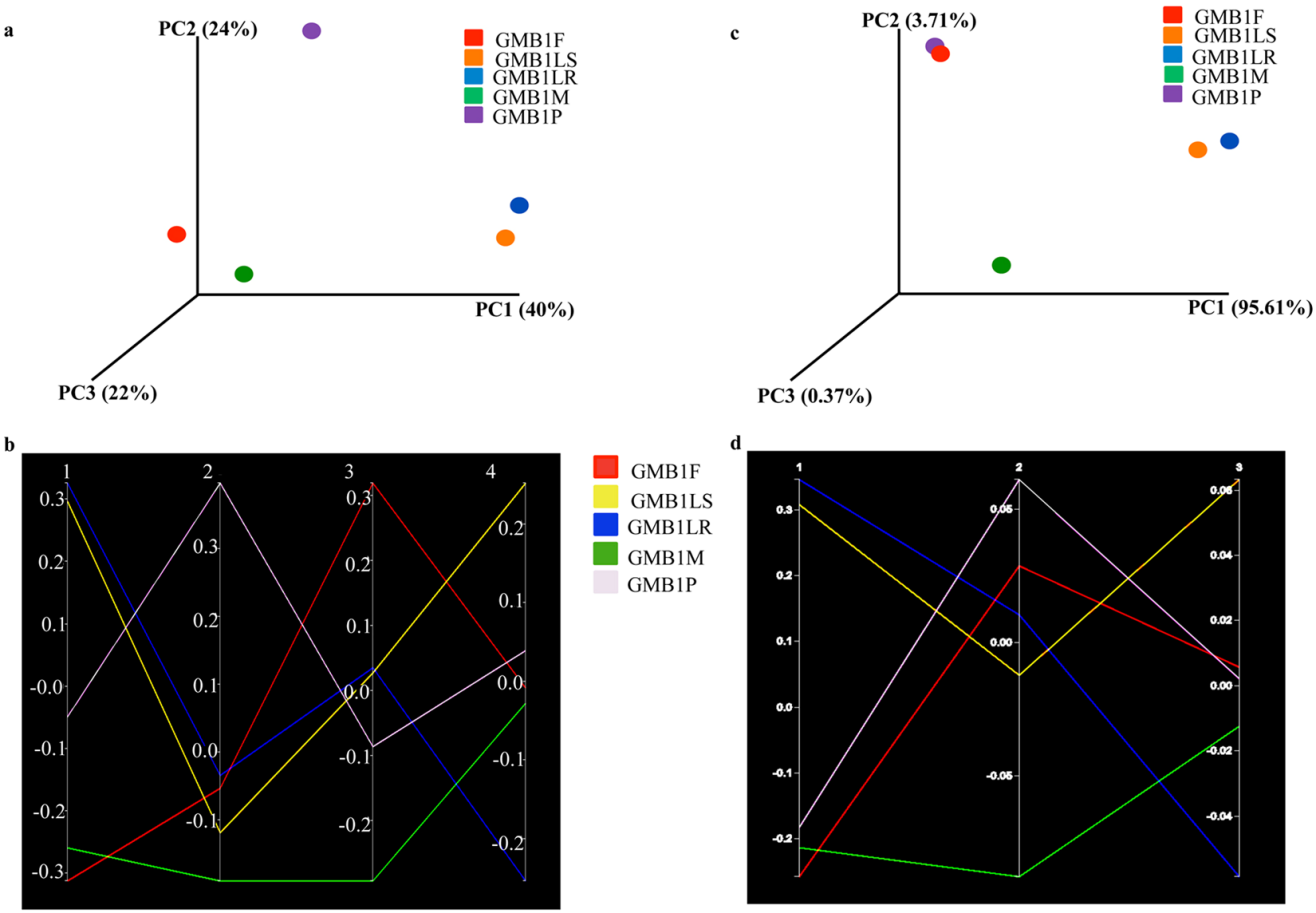
Assessment of microbial diversity in the GMB1 sample using Principal Co-ordinate Analysis (PCoA) (**a**) unweighted 3-D visualization and (**b**) parallel visualization of UniFrac plots to reveal quantitative assessment of microbial diversity between the GMB1 samples. (**c**) Principal Co-ordinate Analysis (PCoA)-weighted 3-D visualization and (**d**) parallel visualization of UniFrac plots to reveal qualitative assessment of microbial diversity between the GMB1 samples.

Beta diversity (using QIIME) analysis for assessing microbial diversity between the GMB1 samples (as represented by principal co-ordinate analysis (PCoA): unweighted and weighted UniFrac) was carried out. Unweighted and weighted UniFrac analysis accounted for the presence/absence of OTUs distribution between samples (GMB1LR, GMB1LS, GMB1P, GMB1M, GMB1F) along axes PC1, PC2, and PC3 (Fig. 3a). Bacterial variance in the GMB1 samples clearly separated GMB1LS, GMB1LR, GMB1P, GMB1F and GMB1M samples in unweighted (Fig. 3a) and weighted (Fig. 3b) plots. The first axis (PC1) showed maximum (40%) bacterial variation in GMB1LS and GMB1LR, followed by the second axis (PC2) 24% bacterial variation in GMB1P, and the third axis (PC3) 22% bacterial variation in GMB1F and GMB1M (Fig. 3a). And in 3-D weighted plot, more than 95% bacterial variation was found in GMB1LS and GMB1LR, followed by 3.71% bacterial variation in GMB1P and GMB1F and 0.37% bacterial variation in GMB1M (Fig. 3b). Similarly, Parallel unweighted and weighted UniFrac analysis revealed maximum bacterial variance in GMB1LS and GMB1LR samples respectively (Fig. 3c,d). PCoA weighted and unweighted UniFrac data was evaluated in pair-wise GMB1 samples comparisons (Tables 2 and 3) using Bonferroni corrected *P*-value. Bonferroni correction was based on randomization of sequence (100) assignments of each pair-wise GMB1 sample. Except one pair, GMB1F and GMB1M, Bonferroni corrected *P*-value 1.0 was calculated for weighted UniFrac analysis of all nine pair-wise GMB1 sample comparisons (Table 2) and Bonferroni corrected *P*-value for unweighted UniFrac analysis of all ten pair-wise comparisons were found to be *P* ≤ 1.0e-02 (Table 3).

**Table 2:** Pair-wise comparison of microbial diversity (based on Principal Co-ordinate Analysis (PCoA)-weighted UniFrac statistical test).

S. No.	Sample name (Group 1)	Sample name (Group 2)	*P*-value	Bonferroni corrected *P*-value
1.	GMB1F	GMB1LR	0.34	1.0
2.	GMB1F	GMB1LS	0.44	1.0
3.	GMB1F	GMB1M	0.05	0.5
4.	GMB1F	GMB1P	0.95	1.0
5.	GMB1LR	GMB1LS	0.79	1.0
6.	GMB1LR	GMB1M	0.48	1.0
7.	GMB1LR	GMB1P	0.40	1.0
8.	GMB1LS	GMB1M	0.46	1.0
9.	GMB1LS	GMB1P	0.49	1.0
10.	GMB1M	GMB1P	0.14	1.0

The Bonferroni corrected *P*-value was calculated from 100 randomizations of bacterial OTUs assignments of the GMB1 samples.

**Table 3:** Pair-wise comparison of microbial diversity (based on Principal Co-ordinate Analysis (PCoA)-unweighted UniFrac statistical test).

S. No.	Sample name (Group 1)	Sample name (Group 2)	*P*-value	Bonferroni corrected *P*-value
1.	GMB1F	GMB1LR	0.0	≤1.0e-02
2.	GMB1F	GMB1LS	0.0	≤1.0e-02
3.	GMB1F	GMB1M	0.0	≤1.0e-02
4.	GMB1F	GMB1P	0.0	≤1.0e-02
5.	GMB1LR	GMB1LS	0.0	≤1.0e-02
6.	GMB1LR	GMB1M	0.0	≤1.0e-02
7.	GMB1LR	GMB1P	0.0	≤1.0e-02
8.	GMB1LS	GMB1M	0.0	≤1.0e-02
9.	GMB1LS	GMB1P	0.0	≤1.0e-02
10.	GMB1M	GMB1P	0.0	≤1.0e-02

The Bonferroni corrected *P*-value was calculated from 100 randomizations of bacterial OTUs assignments of the GMB1 samples.

### Dissimilarity matrix

The dissimilarity between OTUs of the GMB1 samples ranged from 0.04**–**0.47. GMB1LS and GMB1LR showed highest dissimilarity between samples based on OTUs and the maximum similarity was between OTUs from adult female and pupa (dissimilarity index 0.04; Table 4).

**Table 4:** Pair-wise dissimilarity matrix between GMB1 samples based on bacterial OTUs.

Sample name	GMB1F	GMB1LR	GMB1LS	GMB1M	GMB1P
GMB1F	0.0	0.471	0.447	0.127	0.045
GMB1LR	0.471	0.0	0.047	0.431	0.485
GMB1LS	0.447	0.047	0.0	0.406	0.461
GMB1M	0.127	0.431	0.406	0.0	0.136
GMB1P	0.045	0.485	0.461	0.136	0.0

### Bacterial distribution and its comparative study

**Figure 4:**
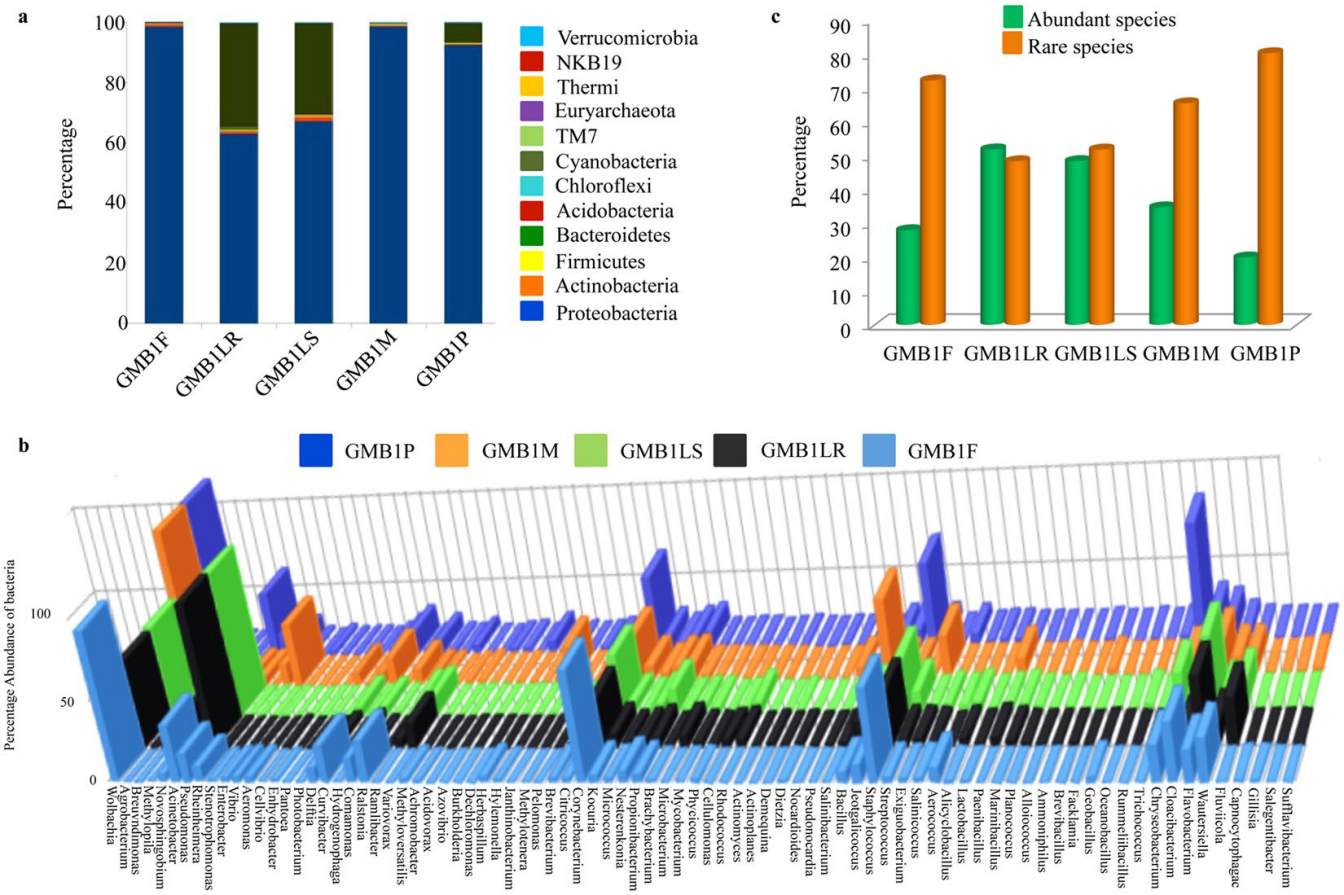
Relative abundance of different bacterial communities among all the GMB1 samples at the level of (**a**) phyla and (**b**) genus. (**c**) Percentage of abundant and rare species among the different GMB1 samples.

Different OTUs identified in GMB1 samples were categorised into different phyla, classes, orders, families, and genera (Table 5, Supplementary Data S3). The relative abundance of different bacterial phyla across all GMB1 samples were identified as follows: Proteobacteria, Actinobacteria, Firmicutes, Bacteroidetes, Acidobacteria, Chloroflexi, Cyanobacteria, TM7, Euryarchaeota, Thermi, NKB19 and Verrucomicrobia (Fig. 4a, Supplementary Data S3).The predominant phylum was Proteobacteria, accounting for 97.8% of the total bacterial sequences. This phylum alone contributed to 98.7%, 94.2%, 94.8%, 98.9%, and 99.1% of the total bacterial sequences in the samples GMB1F, GMB1LR, GMB1LS, GMB1M and GMB1P, respectively. Even though bacterial sequences representing Actinobacteria were second most abundant sequences, it contributed approximately to 1.0% of the total bacterial sequences identified. The remaining sequences representing Firmicutes, Bacteroidetes, Chloroflexi, Planctomycetes, Acidobacteria, Verrucomicrobia, Cynobacteria, Fibrobacteres, Spirochaetes and others, together contributed to <1.0% of the total bacterial sequences (Supplementary Data S4). The major taxa, representing Proteobacteria in GMB1 samples (Fig. 4b), belonged to the genera *Wolbachia* (72.5%), *Pseudomonas* (9.8%), *Enterobacter* (1.9%), *Methylopila* (1.8%), unclassified bacteria (1.6%, belongs to f_Comamonadaceae), *Novosphingobium* (1.2%), unclassified bacteria (1.1%, belongs to f_Enterobacteriaceae), *Acinetobacter* (0.9%), *Stenotrophomonas* (0.7%), unclassified bacteria (0.5%, belongs to f_Sphingomonadaceae), *Pantoea* (0.4%), *Variovorax* (0.3%), unclassified bacteria (0.2%, belongs to f_Pseudomonadaceae), *Rheinheimera* (0.2%), *Breuvndimonas* (0.2%), *Agrobacterium* (0.2%), *Ramlibacter* (0.2%), unclassified bacteria (0.2%, belongs to f_Oxalobacteraceae and o_Rhizobiales), *Delftia* (0.1%) and Actinobacteria (*Corynebacterium* (0.4%) and 0.1% of *Propionibacterium*, *Kouria*, *Nesterenkonia* and *Citricoccus*) (Supplementary Data S4).

**Table 5:** Distribution of bacterial OTUs in the GMB1 samples.

S. No.	Sample name	Phylum	Class	Order	Family	Genus
1.	GMB1F	16	41	79	163	281
2.	GMB1M	21	42	76	158	297
3.	GMB1LS	31	76	143	284	539
4.	GMB1LR	29	84	158	298	578
5.	GMB1P	28	76	138	236	420

In individual samples, *Wolbachia* sp. was predominant in GMB1F (89.8%), GMB1M (79.6%) and GMB1P (97.3%) while *Pseudomonas* sp. was the predominant in GMB1LR (20.9%) and GMB1LS (24.1%). Surprisingly, *Enterobacter* sp. was present at a higher level (7.2%) in GMB1M samples alone while in the other samples of GMB1 its presence was <1%. *Methylopila* sp. was present in higher levels in GMB1LS (8.1%) and GMB1LR (5.3%) while *Novosphingobium* sp. was observed in GMB1F (4.8%) and at 0.1% level in both GMB1LR and GMB1LS. *Acinetobacter* sp. was found at 1.6%, 1.4%, 1.3%, 0.5% and 0.4% levels in GMB1LS, GMB1LR, GMB1M, GMB1F and GMB1P samples, respectively. *Stenotrophomonas* sp. was found in GMB1M (2.5%), GMB1LS (0.2%) and GMB1LR (0.1%) (Supplementary Data S4). Approximately, 37% of the sequences across all samples were unclassified due the unavailability of matching sequences in the GenBank and may represent unique unreported bacterial sequences (Table 6, Supplementary Data S4).

**Table 6:** Unclassified bacterial OTUs (upto 0.1% cut off) across the GMB1 samples.

S. No.	Distribution of unidentified OTUs into family (f)/order (o)	GMB1F (%)	GMB1LR (%)	GMB1LS (%)	GMB1M (%)	GMB1P (%)
1.	f_Comamonadaceae	0.0	6.3	6.3	0.0	0.0
2.	f_Enterobacteriaceae	0.0	0.1	0.0	3.9	0.0
3.	f_Caulobacteraceae	0.0	4.5	4.0	0.0	0.0
4.	f_Sphingomonadaceae	1.9	0.1	0.1	0.0	0.0
5.	f_Pseudomonadaceae	0.1	0.2	0.2	0.6	0.0
6.	f_Oxalobacteraceae	0.0	0.5	0.7	0.0	0.0
7.	o_Rhizbiales	0.0	0.6	0.7	0.0	0.0
8.	f_Aeromonadaceae	0.2	0.2	0.3	0.0	0.1
9.	f_Xanthomonadaceae	0.0	0.1	0.1	0.2	0.1
10.	f_Morasellaceae	0.0	0.3	0.3	0.0	0.0
11.	f_Alphaproteobacteria	0.3	0.0	0.0	0.0	0.0
12.	f_Rhodobacteraceae	0.0	0.2	0.2	0.0	0.0
13.	f_Betaproteobacteria	0.0	0.2	0.2	0.0	0.0
14.	f_Planococcaceae	0.0	0.1	0.1	0.0	0.0
15.	f_Phyllobacteriaceae	0.0	0.1	0.1	0.0	0.0
16.	f_Alteromonadaceae	0.0	0.2	0.1	0.0	0.0
17.	f_Rhodocyclaceae	0.0	0.2	0.1	0.0	0.0
18.	f_Methylobacteriaceae	0.0	0.1	0.1	0.0	0.0
19.	f_Rhizobiaceae	0.0	0.1	0.1	0.0	0.0
20.	f_Micrococcaceae	0.0	0.1	0.1	0.0	0.0
21.	f_Flavobacteriaceae	0.0	0.1	0.0	0.1	0.0
22.	f_Ectothiorhodospira	0.0	0.1	0.1	0.0	0.0
23.	f_Rhodospirillaceae	0.0	0.1	0.1	0.0	0.0
24.	f_Erythrobacteraceae	0.0	0.1	0.1	0.0	0.0
25.	o_Myxococcales	0.0	0.1	0.1	0.0	0.0
26.	f_Pirellulaceae	0.0	0.2	0.0	0.0	0.0
27.	f_Bradyrhizobiaceae	0.0	0.1	0.1	0.0	0.0
28.	f_Intrasporangiaceae	0.0	0.0	0.1	0.0	0.0
29.	o_envOPS12	0.0	0.0	0.1	0.0	0.0
30.	f_A4b	0.0	0.1	0.0	0.0	0.0
31.	f_Nocardioidaceae	0.0	0.1	0.0	0.0	0.0
32.	f_Chitinophagaceae	0.0	0.1	0.0	0.0	0.0
33.	f_Cyclobacteraceae	0.0	0.1	0.0	0.0	0.0
TOTAL		2.5	15.4	14.5	4.8	0.2

### Analysis of abundant and rare species

The bacterial population present in GMB1 samples were represented by both abundant and rare species (the rare species were defined as those with a species frequency of <0.01% of the total population and the remaining ones were considered to be abundant species). In all the samples there was a preponderance of rare species except in GMB1LR. While the rare species were present at 48.2%, 51.7%, 65.3%, 72.1% and 80.1% in GMB1LR, GMB1LS, GMB1M, GMB1F and GMB1P, respectively, the abundant species were present at 19.8%, 27.9%, 34.6%, 48.3% and 51.7% in GMB1P, GMB1F, GMB1M, GMB1LS and GMB1LR, respectively (Fig. 4c).

**Figure 5:**
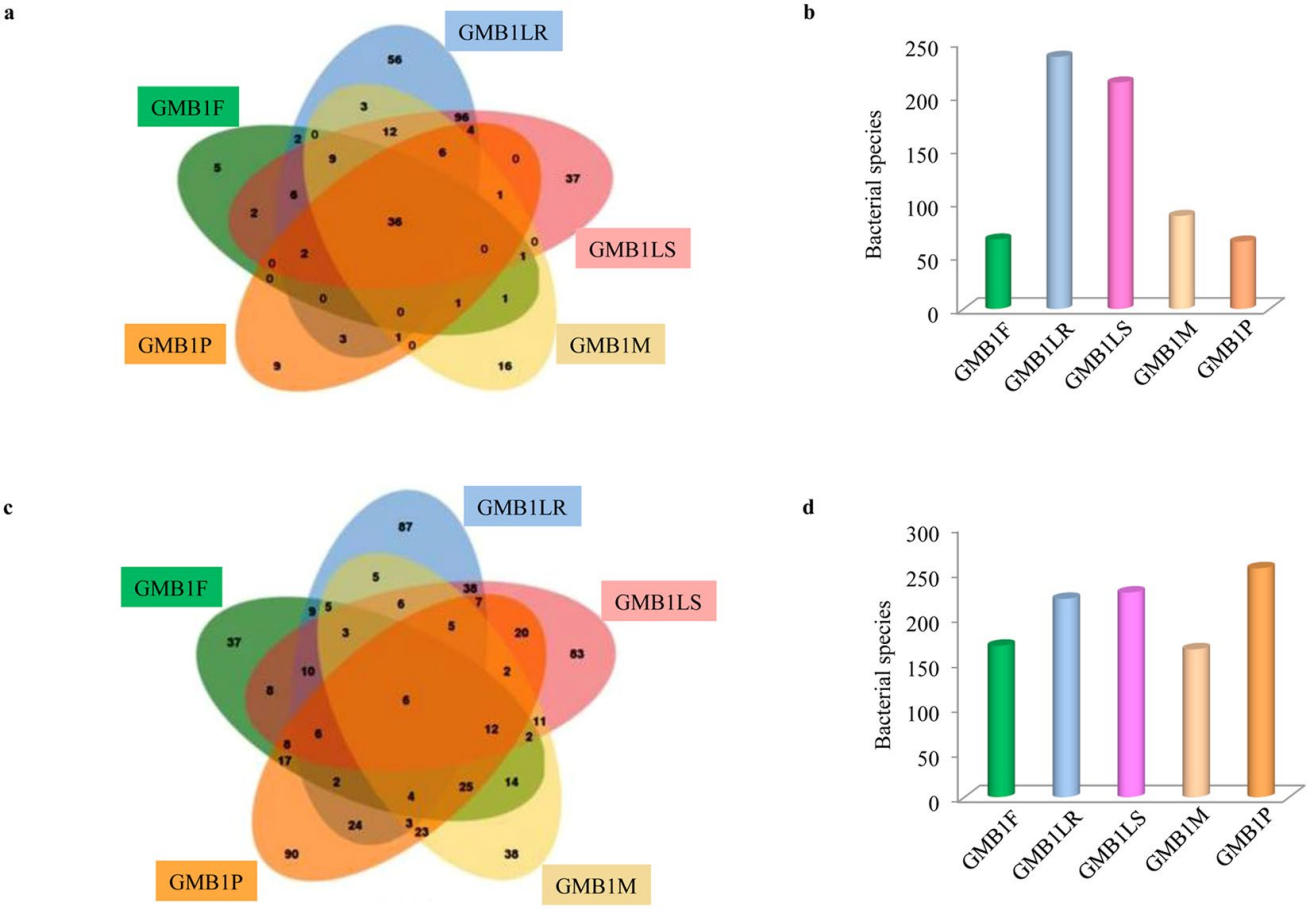
Distribution of abundant and rare bacterial population (shared and specific) in the GMB1 samples. (**a**) Venn diagram and (**b**) bar chart showing distribution of abundant species (shared and specific), (**c**), Venn diagram and (**d**) bar chart showing distribution of rare species (shared and specific) among the GMB1 samples.

A Venn diagram was plotted to calculate the number of specific and shared species among the GMB1 samples (Fig. 5). Across all the samples, 335 rare species and 123 abundant species were uniquely present while the rest (273 rare species and 186 abundant species) were shared among the samples (Fig. 5a–d, Supplementary Data S5). Of the 186 abundant and 273 rare species shared between all the samples, 36 (which includes *Wolbachia* and *Pseudomonas*) of the abundant and 6 species (including *Acinetobacter*) of the rare were commonly present in all GMB1 samples.

### Dynamics of the presence of *Pseudomonas* and *Wolbachia* in developmental stages of the Asian rice gall midge

**Figure 6:**
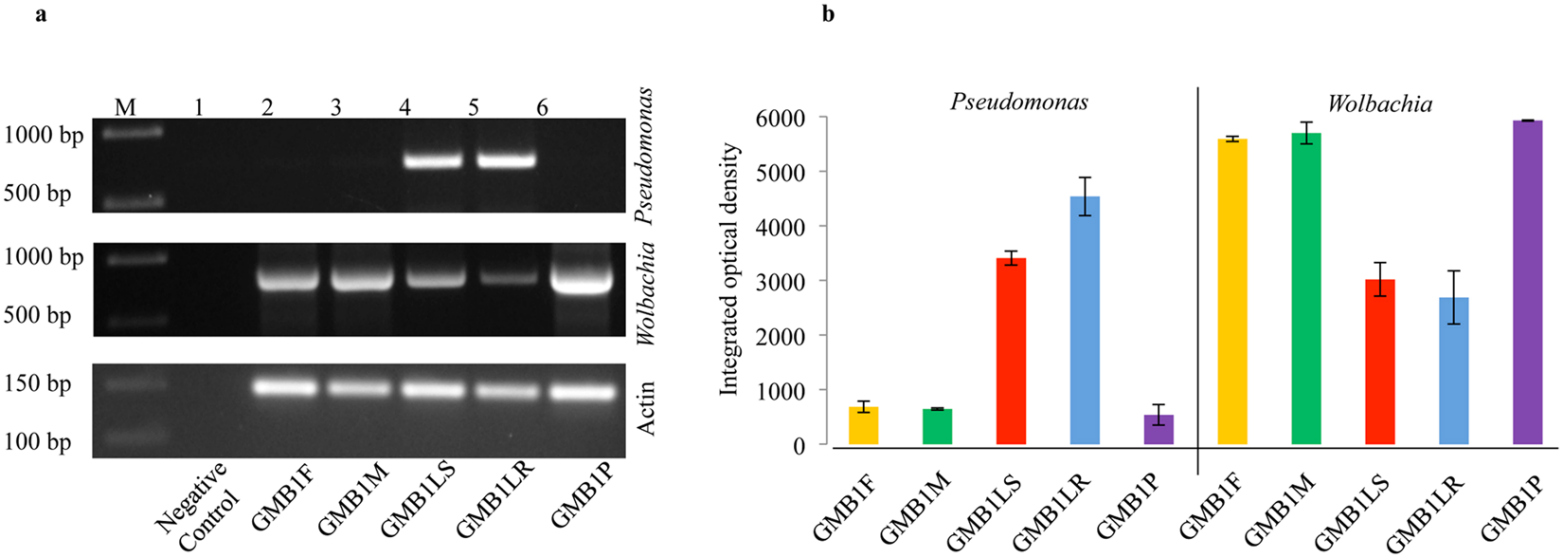
(**a)** Semi quantitative PCR and (**b**) image analyses of the agarose gel in ‘a’, for quantifying abundance of *Pseudomonas* and *Wolbachia* in different GMB1 samples. Actin gene served as the internal control.

To analyse the predominant genera in a developmental stage of the GMB1, we PCR amplified *Pseudomonas*-specific and *Wolbachia*-specific fragments using species-specific primers. The 618 bp *Pseudomonas*-specific fragment was observed in maggots (GMB1LS and GMBILR) dissected 24 hours after infestation (hai). In contrast, we observed a weak amplification of the *Pseudomonas*-specific fragment from GMB1P, GMB1F and GMB1M (Fig. 6). Conversely, the 870 bp *Wolbachia*-specific fragment was predominant in GMB1P, GMB1F and GMB1M, while we observed less amplification of *Wolbachia*-specific fragment in maggots (GMB1LS and GMBILR) (Fig. 6). However, it is yet to be verified whether this dynamic variability in the occurrence of *Pseudomonas* and *Wolbachia* during the different stages of the midge’s life cycle, hints at their probable importance during the developmental stages of the GMB1 within the host.

### Comparison of microbial communities between Asian rice gall midge and Hessian fly

In an effort to understand and correlate the microbiome of the Asian rice gall midge microbial communities with that present in the Hessian fly (also a Cecidomyiid and a major pest of wheat), we carried out a comparative study using microbiome data of the Hessian fly^6^ in the public domain.

The identified OTUs of the first instar maggots of these two gall insects were compared (Table 7) at >97% similarity cutoff. Alpha-proteobacteria and gamma-proteobacteria were present at 78.7 and 16.1%, respectively in the Asian rice gall midge, while in the Hessian fly they were reported at 5.1 and 91.9%, respectively^6^(similar trend observed in *Drosophila* ^7^). *Pseudomonas* sp. was present at 36.9% in the Asian rice gall midge and at 90.2% in the Hessian fly (Table 7).

**Table 7:** Comparative overview of microbial communities identified in maggots of the Asian rice gall midge and the Hessian fly.

S. No.	Reference	Present study	Bansal *et al*., 2014^6^
1.	Insect	*Orseolia oryzae*	*Mayetiola destructor*
2.	Common name	Asian rice gall midge	Hessian Fly
3.	Insect Order	Diptera	Diptera
4.	Member	gall midge	gall midge
5.	Location	Asia	USA
6.	Feed on	Rice	Wheat
7.	Platform used for microbial study	Illumina-MiSeq	454-Pyrosequencing
8.	Targeted 16S-rRNA hypervariable region	V3-V4	V3
9.	Raw Reads from I^st^ instar maggots	782167	6062
10.	High quality reads	739176	3654
11.	Developmental stages for microbial study	I^st^ instar maggots, pupa, adult male and female	I^st^, 2^nd^ & 3^rd^ instar larvae
12.	OTUs picked at	>97% similarity cut-off	>97% similarity cut-off
13.	Predominant proteobacteria in I^st^ instar	97.8%	96.53%
14.	Alpha-proteobacteria in I^st^ instar	78.7%	5.08%
15.	Beta-proteobacteria in I^st^ instar	2.9%	2.96%
16.	Gamma-proteobacteria in I^st^ instar	16.1%	91.89%
17.	Predominant bacteria in I^st^ instar	*Pseudomonas* sp., 36.9%	*Pseudomonas* sp., 90.2%
18.	Predominant bacteria in 2^nd^ instar	ND	*Buttiauxella* sp., 9.7%
19.	Predominant bacteria in 3^rd^ instar	ND	*Buttiauxella* sp.,
20.	Predominant bacteria in pupa	*Wolbachia* sp., 97.3%	ND
21.	Predominant bacteria in adult female	*Wolbachia* sp., 89.8%	ND
22.	Predominant bacteria in adult male	*Wolbachia* sp., 79.6%	ND

(ND; Not determined).

## Discussion

Currently, there is very little information with regard to the role of ARGM microbiome in the gall midge-rice interaction. Microscopic first instar rice gall midge maggot and lack of artificial diets make the studies on ARGM difficult^8^. But more importantly, we expect that the identification of constituents of the microbiome of the rice gall midge would yield valuable information regarding their likely role in the gall midge’s interaction with its rice host^9–11^, if any. In addition, it would be worth investigating whether gall midge maggots feeding on resistant rice host are pushed towards death due to a decrease in the number of beneficial microbes within them. Hence, the present study was initiated for the identification of microbial communities and to obtain a better understanding of the microbiome in the interaction between the rice and gall midge.

Our current study identified several distinct OTUs, based on the NGS sequencing of the V3-V4 region of the 16S rRNA gene, in 5 different samples of the ARGM. Members of Proteobacteria were the predominant bacterial constituents in all GMB1 samples and this could be due to the fact that during the feeding stage of the Asian rice gall midge, the maggots (GMB1LS and GMB1LR) could be actively recruiting Proteobacteria from the rice hosts and these then could be actively transferred from the maggots to the pupal stage and finally to adults^10–12^. Moreover, GMB1LS and GMB1LR samples, representing the only feeding stage of the rice gall midge, showed the highest alpha diversity indices (Table 1). It may be noted that member of the Proteobacteria has been reported as the major bacterial population in the biosphere^13^ due to their efficient colonization ability^14–17^. Proteobacteria have also been reported as the predominant bacteria in wild *Drosophila*–a polyphagous dipteran insect^7^. This is true for hematophagous insects as well i.e. bed bugs^18^ and mosquitoes (*Aedes albopictus* and *Aedes aegypti*)^19^ as well as ticks^20^.

*Wolbachia* sp and *Pseudomonas* sp formed the major constituents of the Proteobacteria in all the GMB1 samples analysed. *Wolbachia* sp has been described as a microbe with a capacity to change several traits of its host. However, it is not known whether *Wolbachia*, present within the gall midge, imparts any benefits to its host during the gall midge-rice interaction. It has been shown previously that phenotypes mediated by *Wolbachia* influence insulin signalling and glucose metabolism in *Drosophila* ^21, 22^, male killing in ladybird (*Adaliabibi punctata*)^23^ and butterflies (*Acraea encedon*, *Hypolimnas bolina*)^23–25^, feminization of host in a wide variety of invertebrates including insects, spiders, crustaceans and nematode worms^26^, sex ratio distortion and affect fecundity in terrestrial isopod *Oniscus asellus* ^27^, manipulate sex life and boosts reproductive success in Ugandan butterflies^28^. Also, an earlier study has shown that there is correlation between gall midge mitotypes and infection status of ARGM with respect to *Wolbachia* ^29^. However, currently there is no information whether *Wolbachia* present within the ARGM exerts any influence on the rice - gall midge interaction.

*Pseudomonas*, a prominent constituent of Proteobacteria, was also present in high numbers in all the GMB1 samples. *Pseudomonas* sp present in agricultural pests such as *Plutella xylostella*, *Spodoptera exigua*, *Spodoptera litura*, *Trichoplusia ni* is known to be involved in biopesticide breakdown^30–32^. Therefore, it is likely to play a similar role in the Asian rice gall midge as well. Taking this observation further, we speculate that *Pseudomonas* may also play a role in the breakdown of plant defence biomolecules produced by the rice host in response to the gall midge infestation. However, this is yet to be verified. Furthermore, it has been shown that *Pseudomonas* play an important role in protecting silkworm (*Bombyx mori*) larvae from oxidative stress and bacterial infections^33^. Whether a similar role is played by *Pseudomonas* in the rice gall midge is yet to be elucidated.

The microbiome of the ARGM was made up of both rare and abundant species. The presence of these rare and abundant species which are unique to a particular sample, may be indicative of its crucial role in biological function of that particular stage in the life cycle of ARGM and might facilitate its interaction with the rice host. Maggots (GMB1LR) on resistant rice host survived only for 48–96 hours^1^ after infestation (hai) and during this time period maggots stop feeding and die and are thus unable to induce galls on such resistant rice. In contrast, maggots (GMB1LS) feeding on susceptible rice host continue feeding and are able to develop further, complete their life cycle and emerge from the galls as adults. Compared to the bacterial population observed in GMB1LS, the maggots (GMB1LR) dissected from resistant rice plant showed reduced bacterial population of those belonging to Actinobacteria, Bacteriodetes, Proteobacteria while there was an increase in the population of bacteria belonging to Cyanobacteria and Planctomycetes (Supplementary Table 3a,b, Supplementary Data S6). This reduction of several bacterial populations in GMB1LR within 48 hai could have a bearing on GMB1LR mortality as hypothesized for humans where host stability and demise are correlated to change in the indigenous microbiome^34^.

Insect diets are often nutrient-poor or incomplete and some insects have specialized cells, called bacteriocytes that house beneficial intracellular bacteria considered to be responsible for supplementing the otherwise nutrient-poor diets of insects^35, 36^. Facultative bacteria such as *Enterobacter* and lactic-acid bacteria have been found in GMB1 samples and both these have been reported to promote insect fitness by contributing essential amino acids and vitamins to their host. Further, during molting from 1^st^ instar to 2^nd^ instar and to subsequent instar, followed by pupa and finally into adults, associated proteobacteria could be involved in host survival by synthesizing and providing essential nutrients^37^ during the molting period which are probably essential during the non-feeding stages in the insect’s life cycle.

Insect metamorphosis is a metabolically dynamic and complex process and it is likely that the insect-associated microbes play a role in facilitating metamorphosis^38^. Moreover, it is believed that during the different stages of metamorphosis there is a sterilization process at each stage and insects acquire new set of microbes at each stage^39, 40^. Though the present study did not reveal metamorphic stage-specific microbes, results indicate fluctuations in the relative abundance of these microbes at each stage of life cycle of the midge. It is interesting to note that while *Wolbachia* is predominant in GMB1F, GMB1M and GMB1P, *Pseudomonas* is predominant in GMB1LR and GM1LS and one reason for this observed phenomenon could be that the larval stages (GMB1LR and GM1LS) represent the only feeding stage in the life cycle of the Asian rice gall midge and therefore, *Pseudomanas* could be helping maggots in the breakdown of plant allelochemicals. In the subsequent non-feeding stages, the role of *Pseudomanas* may not be that predominant and therefore, not maintained. Moreover, data indicate that though *Wolbachia* is not predominant in GMB1LR and GM1LS samples, they are the second most abundant ones. Therefore, as the larval stages metamorphose to pupal and adult stages the population of *Pseudomonas* reduces and that of *Wolbachia* predominates in both the later stages. Certainly, concerted studies would be required to determine whether these microbes could be involved in bringing about changes in the internal morphology and physicochemical conditions that accompany metamorphosis in GMB1.

The results obtained from present study indicated that microbes present in the ARGM are predominantly members of the Proteobacteria. This is also true for another economically important member of Cecidomyiidae, the Hessian fly (*Mayetiola destructor*). It is also reasonable to assume that microbial diversity observed in GMB1 is likely due to acquisition of different bacterial species from the external environment. The microbial diversity in GMB1 may influence GMB1-rice interaction and could contribute to gall formation in susceptible rice hosts. To the best of our knowledge, this is the first attempt of a comparative cataloguing of the microbiome in the different stages of the ARGM life cycle using a high-throughput next generation sequencing protocol. In addition, the extensive microbiome catalogue thus established (through data obtained from PCoA UniFrac analyses) will enable future studies involving association of the microbial genes with insect phenotypes and, even more broadly, its influence on insect interaction with its host and also metamorphosis.

## Methods

### Sample Collection

The GMB1 colony used in this study is being maintained in the greenhouse at the Indian Institute of Rice Research (IIRR) (formerly Directorate of Rice Research), Hyderabad, India under standard conditions^41^. Briefly, pre-germinated seeds of different rice varieties were grown in rows, 5 cm apart, with 10–15 plants per row, in plastic trays with 8 cm deep, puddled, fertilizer-enriched, soil. 10–15-days-old plants were exposed to about 50 females and 10–20 males. On the third day, the trays were shifted to a high humidity (90–100% relative humidity) chamber and left undisturbed for egg incubation and maggot establishment. Maggots were isolated from the rice varieties TN1 and RP2068-18-3-5 (henceforth RP). GMB1 is virulent on TN1 (susceptible to GMB1; compatible interaction) and avirulent on RP (resistant to GMB1; incompatible interaction). Maggots feeding on TN1 complete their development and emerge as adults while those feeding on RP die within 96 h post hatching. Hence, first instar maggots were collected individually from both TN1 and RP within 96 h using entomological needles and stored in 100% alcohol or RNAlater (Qiagen, USA) for further use. Care was taken not to injure the maggots. Approximately 150 maggots from each rice variety (TN1 and RP) were collected for the whole experiment. In addition, several pupae and adults were also collected from infested TN1. These stages will not be available, obviously, from RP.

### DNA extraction and PCR amplification of 16S ribosomal V3-V4 region from ARGM

DNA was extracted from maggots, pupae and adults using HiPurA insect DNA purification kit (Himedia, India) as per manufacturer’s instructions. DNA concentrations were estimated by NanoVue Plus (GE Healthcare, UK). 30 ng of extracted DNA was used as a template for PCR to amplify the V3-V4 region of the 16S rRNA region using the primers V3-341F 5′-CCTACGGGNGGCWGCAG-3′ and V4-805R 5′-GACTACHVGGGTATCTAATCC-3′ (see Klindworth *et al*.^42^). PCR was carried out in 25 μl reaction volume containing 200 μM dNTPs, 5U *Taq* DNA polymerase (Promega Corporation, USA) and 13 μM of each of the primers. PCR conditions were 95 °C for 2 min, followed by 25 cycles at 95 °C for 30 sec, 55 °C for 30 sec, 72 °C for 30 sec, with a final extension step at 72 °C for 5 min. The amplified PCR products (~460 bp) from each sample were run on 1% agarose gel and gel-purified using QIAquick Gel Extraction kit (Qiagen, Inc, Germany) and quantified using NanoVue Plus spectrophotometer. The purified ~460 bp PCR products from each sample were sent to M/s Genotypic Technology Pvt. Ltd. (India) for metagenomic sequencing of the amplicon (16S rRNA V3-V4 region) using NGS protocol on an Illumina-MiSeq platform.

### Library Preparation and Illumina-MiSeq Sequencing

The library preparation followed the general library construction protocol as detailed in the 16S metagenomic sequencing library preparation methodology: (http://support.illumina.com/downloads/16s_metagenomic_sequencing_library_preparation.html). Gel purified fragment (~2 ng), of each sample was used for re-amplifying V3-V4 region of 16S rRNA region with specific primers that possess a tag sequence that are complementary to the Illumina sequence adapter and index primers from the Nextera XT Index kit V2 (IIIumina, Cat # FC-131-2002) as per manufacture’s protocol. This generated ~530 bp amplicons. Amplification was verified on 1% agarose gel before proceeding for indexing PCR. In the next round of PCR (indexing PCR), Illumina sequencing adapters and dual indexing barcodes were added using limited cycle PCR to generate a final product of ~600 bp. The library was cleaned using High Prep PCR post PCR clean up system (MagBio Genomics, Inc., USA). Further, quality control check of the library was performed using a Bioanalyzer Chip (Agilent Technologies, USA). PCR products with unique indices from each library were taken in equal (~2 ng) quantities and subjected to 300 paired-end multiplex sequencing using Illumina-MiSeq platform.

### Initial Processing and bioinformatics analysis of sequence reads

QIIME (www.qiime.org)^43^ platform was used to extract high-quality sequences. Raw reads were selected based on read length, quality, primer and tag. The raw reads were quality checked using SeqQC v2.1 (genotypic.co.in/SeqQC.html), a proprietary software available with Genotypic Technology Pvt. Ltd, Bangalore, India. Based on the following criteria, the total sequences were screened to remove: (i) raw reads <110 nucleotides, (ii) reads with adapter sequences, (iii) raw reads with primer sequences, barcode and degenerate bases, (iv) reads that had a quality (phred) score of less than 20, (v) duplicates from processed reads using CD-HIT-DUP^44^, (vi) chimeric sequences from the operational taxonomic units (OTUs). The screened sequences were aligned to obtain representative sequences. The unique sequences set was classified into OTUs under threshold of 97% identity with phred score >20. The identity of each OTU sequence was confirmed using the microbial 16S ribosomal Greengenes database (Lawrence Berkeley National Laboratory, http://greengenes.lbl.gov). Next, a de novo taxonomic tree was constructed employing chimera-checked OTU set in QIIME (www.qiime.org)^43, 45^ for downstream analysis which included estimation of alpha and beta diversity. The Shannon-Wiener^46^, Simpson’s diversity^47^, the Chao1^48^ and rarefaction curve were calculated in order to evaluate the alpha diversity. Consequently, the phylogenetic relationships among the individuals were estimated to provide a complete picture of alpha diversity. UniFrac distance was based on the phylogenetic tree by QIIME. To estimate the dissimilarities between the different samples, principal co-ordinate analysis (PCoA) was carried out. Both weighted and unweighted calculations were performed for the PCoA (www.qiime.org).

### Nucleotide Sequence accession number

Paired end Illumina sequence data from this study was submitted to the NCBI Sequence Read Archive (SRA) under accession number SRP064774.

### PCR amplification of 16S ribosomal DNA (rDNA) of *Wolbachia* and *Pseudomonas* from the Asian rice gall midge samples

*Wolbachia*-specific primers^29^ (forward: 5′-TTGTAGCCTGCTATGGTATAACT-3′ and reverse: 5′-GAATAGGTATGATTTTCATGT-3′) and *Pseudomonas*-specific primers^49^ (forward: 5′-GACGGGTGAGTAATGCCA-3′ and reverse: 5′-CACTGGTGTTCCTTCCTATA-3′) were used for PCR amplification of ~870 bp and ~618 bp 16S regions from *Wolbachia* and *Pseudomonas*, respectively to quantify their relative presence in the different developmental stages of the GMB1. The actin gene (~149 bp) of the ARGM^50^ was used as the internal control. Genomic DNA (10 ng) of maggots, pupae and adults from TN1 and genomic DNA (20 ng) of maggots from RP were used for PCR. Twenty five μl PCR reaction mix contained 200 μM dNTPs, 2.0 U of *Taq* DNA polymerase (Agilent Technologies, USA) and 12 μΜ of each primer. The 16S region of *Wolbachia* and actin gene were PCR amplified from each sample using the following conditions: an initial denaturation of 5 min at 95 °C followed by 30 cycles of 30 s at 95 °C, 1 min at 55 °C, and 1 min at 72 °C, with a final extension of 5 min at 72 °C. The 16S region of *Pseudomonas* sp. was PCR amplified using an initial denaturation of 5 min at 95 °C followed by 30 cycles of 30 s at 95 °C, 30 s at 58 °C, and 45 s at 72 °C, with a final extension of 5 min at 72 °C. The PCR amplified products were analysed on 1% agarose gel. The gels were photographed using a gel documentation system (Alpha Imager, Cell Biosciences, UK) and the images were captured in JPEG format. The images were subsequently analysed and relative intensities of each fragment was estimated using Image J software (http://imagej.nih.gov/ij/).

### Statistical Analysis

Rarefaction analysis and alpha diversity index (Shannon and Simpson’s diversity index, Chao1) were used to estimate the differences in microbial communities between GMB1 samples (obtained from both TN1 and RP) used in the present study. The phylogeny-based unweighted UniFrac distance metric, and PCoA plots and rarefaction curves were plotted using QIIME (www.qiime.org). To visualize the relative abundance of bacterial phyla for individual GMB1 samples, heat maps were generated using QIIME.

## Electronic supplementary material

Supplementary Files

Supplementary data 1

Supplementary data 1

Supplementary data 1

Supplementary data 1

Supplementary data 1

Supplementary data 3

Supplementary data 3

Supplementary data 3

Supplementary data 3

Supplementary data 3

Supplementary data 4

Supplementary data 5

## References

[1] Bentur JS (2016). Rice-gall midge interactions: Battle for survival. J. Insect Physiol..

[2] Sinha DK, Atray I, Bentur JS, Nair S (2015). Feeding on resistant rice leads to enhanced expression of defender against apoptotic cell death (*OoDAD1*) in the Asian Rice gall midge. BMC Plant Biol..

[3] Agarrwal R, Bentur JS, Nair S (2014). Gas chromatography mass spectrometry based metabolic profiling reveals biomarkers involved in rice-gall midge interactions. J. Int. Plant Biol..

[4] Sinha DK, Atray I, Agarrwal R, Bentur JS, Nair S (2017). Genomics of the Asian rice gall midge and its interactions with rice. Curr. Opin. Insect Sci..

[5] Felton GW, Tumilson JH (2008). Plant-insect dialogs: complex interactions at the plant-insect interface. Curr. Opin. Plant Biol..

[6] Bansal R (2014). Pyrosequencing reveals the predominance of pseuomonadaceae in gut microbiome of a gall midge. Pathogens.

[7] Broderick NA, Lemaitre B (2012). Gut-associated microbes of *Drosophilla melanogaster*. Gut Microbes.

[8] Sinha DK, Bentur JS, Nair S (2011). Compatible interaction with its rice host lads to enhanced expression of gamma subunit of oligosaccharyl transferase (*OoOST*) in the Asian rice gall midge (*Orseolia oryzae*). Insect Mol. Biol..

[9] Yun JH (2014). Insect gut bacterial diversity determined by environmental habitat, diet, developmental stage, and phylogeny of host. Appl. Environ. Microbiol..

[10] Jones RT, Sanchez LG, Fierer N (2013). A cross-taxon analysis of insect-associated bacterial diversity. PLos One.

[11] Kikuchi Y (2009). Endosymbiotic bacteria in insects: their diversity and culturability. Microbes Environ..

[12] Douglas AE (2015). Multiorganismal insects: diversity and function of resident microorganisms. Annu. Rev. Entomol..

[13] Galand PE, Casamayor EO, Kirchman DL, Lovejoy C (2009). Ecology of the rare microbial biosphere of the Arctic Ocean. Proc. Natl. Acad. Sci. USA..

[14] Maheshwari, D.K. *Bacterial Diversity in Sustainable Agriculture*. Springer, Cham Heidelberg New York Dordrecht London (2014).

[15] Roesch LF (2007). Pyrosequencing enumerates and contrasts soil microbial diversity. ISME J..

[16] Arruda L (2013). Screening of Rhizobacteria isolated from maize (*Zea mays* L.) in Rio Grande do Sul State (South Brazil) and analysis of their potential to improve plant growth. Appl. Soil Ecol..

[17] Rampelotto PH, de Siqueira Ferreira A, Barboza AD, Roesch LF (2013). Changes in diversity, abundance, and structure of soil bacterial communities in Brazilian Savanna under different land use systems. Microb Ecol..

[18] Meriweather M, Mathews S, Rio R, Baucom RS (2013). A 454 survey reveals the community composition and core microbiome of the common bed bug (*Cimex lectularius*) across an Urban Landscape. PLoS One.

[19] Zouache K (2011). Bacterial diversity of field-caught mosquitoes, *Aedes albopictus* and *Aedes aegypti*, from different geographic regions of Madagascar. FEMS Microbiol. Ecol..

[20] Narasimhan S, Fikrig E (2015). Tick microbiome: the force within. Trends Parasitol..

[21] Clark ME, Anderson CL, Cande J, Karr TL (2005). Widespread prevalence of *Wolbachia* in laboratory stocks and the implications for *Drosophila* research. Genetics.

[22] Iturbe-Ormaetxe I, O’Neill SL (2007). *Wolbachia*-host interactions: connecting phenotype to genotype. Curr. Opin. Microbiol..

[23] Hurst GDD (1999). Male-killing *Wolbachia* in two species of insect. The Royal Society Proceeding B.

[24] Charlat S (2007). Extraordinary flux in sex ratio. Science.

[25] Charlat S (2007). Male-killing bacteria trigger a cycle of increasing male fatigue and female promiscuity. Curr. Biol..

[26] Knight J (2001). Meet the Herod bug. Nature.

[27] Rigaud T, Moreau J, Juchault P (1999). *Wolbachia* infection in the terrestrial isopod *Oniscus asellus*: sex ratio distortion and effect on fecundity. Heredity (Edinb).

[28] Zimmer C (2001). *Wolbachia* - A tale of sex and survival. Science.

[29] Behura SK, Sahu SC, Mohan M, Nair S (2001). *Wolbachia* in the Asian rice gall midge, *Orseolia oryzae* (Wood-Mason): correlation between host mitotypes and infection status. Insect Mol. Biol..

[30] Chen WJ (2014). Characterization of an insecticidal toxin and pathogenicity of *Pseudomonas taiwanensis* against insects. PLoS Pathog..

[31] Matsumura F, Boush GM (1967). Dieldrin: degradation by soil microorganism. Science.

[32] O’Mahony MM, Dobson AD, Barnes JD, Singleton I (2006). The use of ozone in the remediation of polycyclic aromatic hydrocarbon contaminated soil. Chemosphere.

[33] Su LD, Zhang QL, Lu ZQ (2015). Oxidation resistance 1 (OXR1) participates in silkworm defense against bacterial infection through the JNK pathway. Insect Sci..

[34] Blaser MJ, Webb GF (2014). Host demise as a beneficial function of indigenous microbiota in human hosts. mBio..

[35] Jones RT, Sanchez LG, Fierer N (2013). A cross-taxon analysis of insect-associated bacterial diversity. PLoS One.

[36] Baumann, P., Moran, N.A., Baumann, L.C. Bacteriocyte-associated Endosymbionts of Insects. In *The Prokaryotes* **1**, 403-438 (ed. Dworkin, M., Falkow, S., Rosenberg, E., Schleifer, K-H., Stackerbrandt, E.) (Springer link, 2006).

[37] Douglas AE (2009). The microbial dimension in insect nutritional ecology. Funct. Ecol..

[38] Hammer TJ, McMillan WO, Fierer N (2014). Metamorphosis of a butterfly-associated bacterial community. PLoS One.

[39] Rani A, Sharma A, Rajagopal R, Adak T, Bhatnagar RK (2009). Bacterial diversity analysis of larvae and adult midgut microflora using culture-dependent and culture-independent methods in lab-reared and field-collected *Anopheles stephensi*- an Asian malarial vector. BMC Microbiol..

[40] Moll RM (2001). Meconial peritrophic membranes and the fate of midgut bacteria during mosquito (Diptera: Culicidae) metamorphosis. J. Med. Entomol..

[41] Sinha DK, Atray I, Bentur JS, Nair S (2012). Expression of *Orseolia oryzae nucleoside diphosphate kinase* (*OoNDPK*) is enhanced in rice gall midge feeding on susceptible rice hosts and its over-expression leads to salt tolerance in *Escherichia coli*. Insect Mol. Biol..

[42] Klindworth A (2013). Evaluation of general 16S ribosomal RNA gene PCR primers for classical and next-generation sequencing-based diversity studies. Nucleic Acids Res..

[43] Caporaso (2010). QIIME allows analysis of high-throughput community sequencing data. Nat. Methods.

[44] Li W, Godzik A (2006). Cd-hit: a fast program for clustering and comparing large sets of protei or nucleotide sequences. Bioinformatics.

[45] Lozupone CA, Knight R (2008). Species divergence and the measurement of microbial diversity. FEMS Microbiol. Rev..

[46] Shannon, C.E., Weaver, W. *The Mathematical Theory of Communication*. University of Illinois Press; Urbana, Il (1949).

[47] Simpson EH (1949). Measurement of diversity. Nature.

[48] Chao A (1984). Non-parametric estimation of the number of classes in a population. Scand. J. Stat..

[49] Spilker T, Coenye T, Vandamme P, LiPuma JJ (2004). PCR-based assay for differentiation of *Pseudomonas aeruginosa* from other *Pseudomonas* species recovered from cystic fibrosis patients. J. Clin. Microbiol..

[50] Sinha DK, Lakshmi M (2011). Serine proteases-like genes in the Asian rice gall midge show differential expression in compatible and incompatible interactions with rice. Int. J. Mol. Sci..

