# Supplementary material for: Bacterial Community Structure in the Asian Rice Gall Midge Reveals a Varied Microbiome Rich in Proteobacteria
Source: Sci Rep. 2017 Aug 25;7:9424. doi: 10.1038/s41598-017-09791-0 (PMC5573367; doi:10.1038/s41598-017-09791-0)

Bacterial Community Structure in the Asian Rice Gall Midge Reveals a Varied Microbiome Rich in Proteobacteria

Abhishek Ojha, Deepak Kumar Sinha, A. P. Padmakumari, J. S. Bentur, Suresh Nair

Supplementary Table 1| Quantification of the gel purified PCR product of the V3-V4 region of the 16S ribosomal DNA from the Asian rice gall midge biotype 1 (ARGMB1) DNA.

S. No.	Sample name	Concentration (ng/µl)	Volume (µl)	Yield (ng)
1.	GMB1F	1.44	15	21.6
2.	GMB1M	1.48	15	22.2
3.	GMB1LS	1.09	15	16.35
4.	GMB1LR	1.27	15	19.05
5.	GMB1P	1.66	15	24.9

Supplementary Table 2| Overview of Illumina-MiSeq sequencing results of the V3-V4 region of the 16S ribosomal DNA amplified from the Asia rice gall midge biotype 1 (ARGMB1) samples.

S. No.	Sample name	Total reads (10^6)	Processed reads (10^6)	HQ reads* (10^6)	% of HQ reads	Stitch reads (10^6)	CD-HIT DUP analysis (10^6)
1.	GMB1F	0.91	0.87	0.87	96.1	0.95	0.39
2.	GMB1M	0.88	0.85	0.84	96.1	0.92	0.42
3	GMB1LS	0.78	0.75	0.74	94.5	0.86	0.51
4.	GMB1LR	0.75	0.72	0.70	94.0	0.82	0.45
5.	GMB1P	1.00	0.96	0.95	95.0	1.05	0.49
	Total	4.32	4.15	4.1	-	4.6	2.26

HQ: High Quality, **(*)** >70% of bases have phred score >20

Supplementary Table 3| Comparison of bacterial population in Maggots (GMB1LR vs GMB1LS). **a**, Reduction of bacterial population in GMB1LR when compared with GMB1LS at phyla level, **b**, Increase of bacterial population in GMB1LR when compared with GMB1LS at phyla level.

a.

Taxonomy	GMB1LR (%)	GMB1LS (%)
p_Actinobacteria	0.7	1.4
p_Bacteriodetes	0.5	0.6
p_Proteobacteria	63.2	67.3

b.

Taxonomy	GMB1LR (%)	GMB1LS (%)
p_Cyanobacteria	34.8	30.1
p_Planctomycetes	0.1	0.0

Supplementary Figure 1| Multiple exposures of Figure 1a

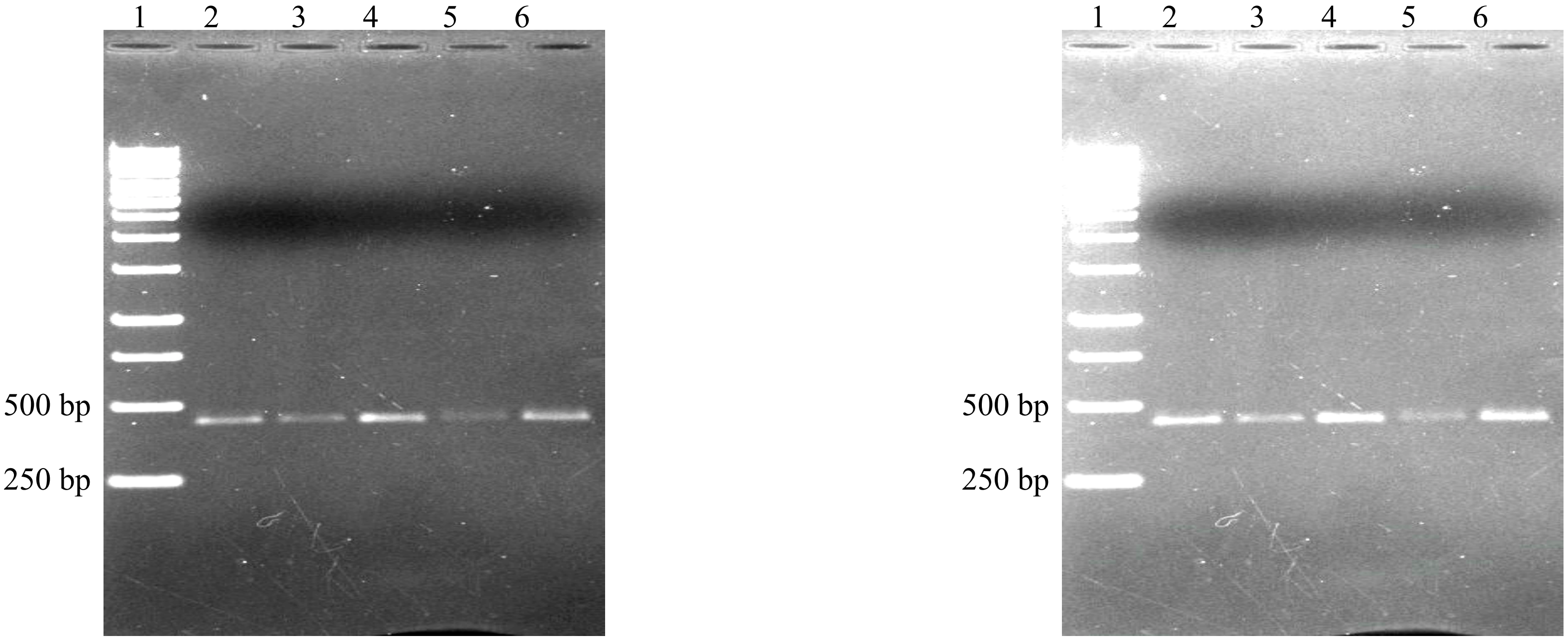

Supplementary data 2| Graphical representation of the relative abundance of bacterial diversity from phylum to species level of GMB1F, GMB1LR, GMB1LS, GMB1M and GMB1P samples. This data can be visualized using Krona visualization tool. This file will help visualize the relative distribution and abundance of the complex bacterial community composition of the sample through simple graphical representation.

GMB1F

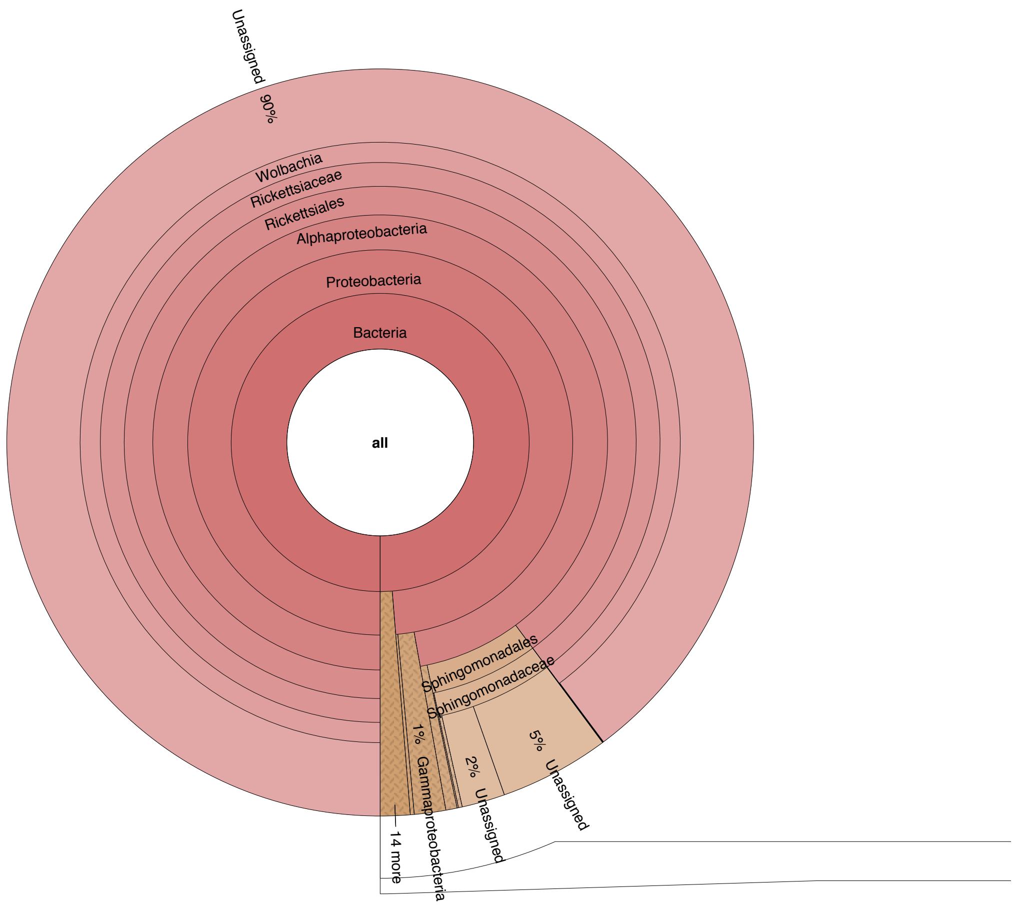
Unassigned 90%
Wolbachia
Rickettsiaceae
Rickettsiales
Alphaproteobacteria
Proteobacteria
Bacteria
all
Sphingomonadales
Sphingomonadaceae
5% Unassigned
2% Unassigned
1% Gammaproteobacteria
14 more

GMB1LR

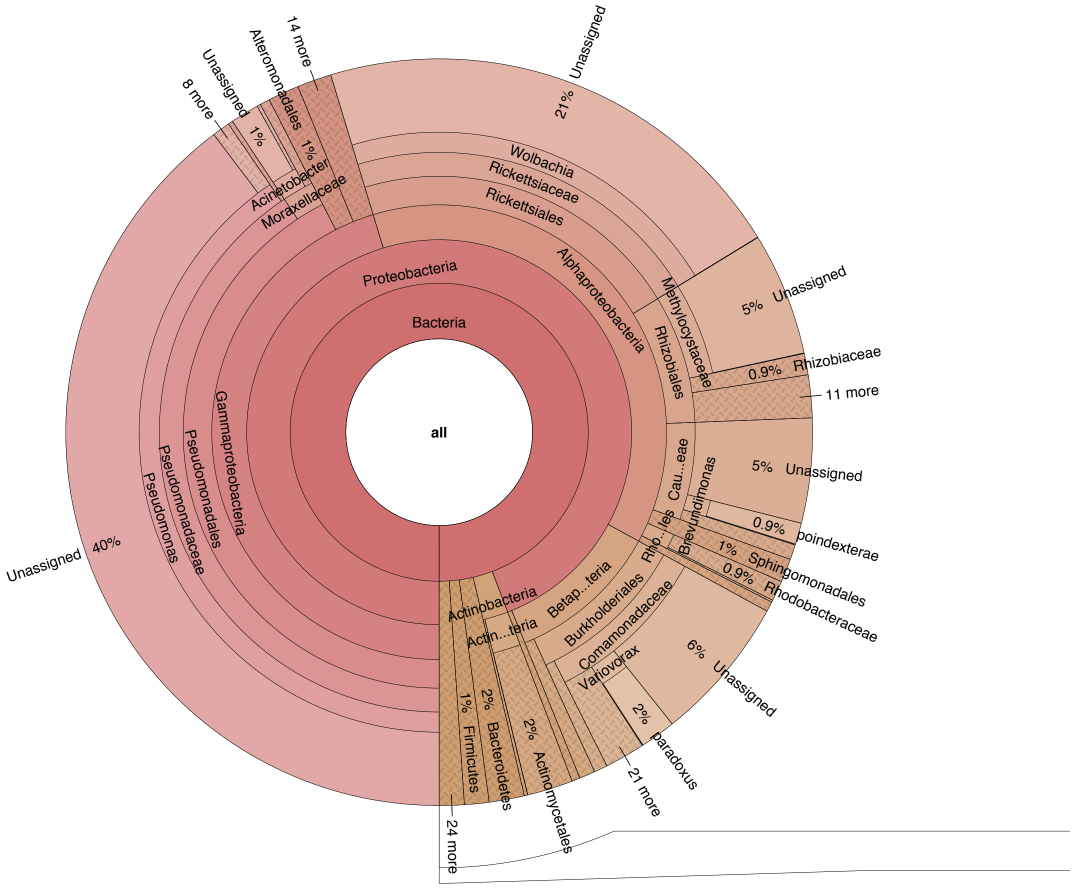
all
Bacteria
Proteobacteria
Gammaproteobacteria
Pseudomonadales
Pseudomonadaceae
Pseudomonas
Unassigned 40%
Moraxellaceae
Acinetobacter
Alteromonadales
1%
Unassigned
1%
8 more
14 more
Alphaproteobacteria
Rickettsiales
Rickettsiaceae
Wolbachia
21% Unassigned
Rhizobiales
Methylocystaceae
5% Unassigned
0.9% Rhizobiaceae
11 more
Cau...eae
Brevundimonas
5% Unassigned
0.9% poindexterae
Rho...les
1% Sphingomonadales
0.9% Rhodobacteraceae
Betap...teria
Burkholderiales
Comamonadaceae
Variovorax
6% Unassigned
2% paradoxus
21 more
Actinobacteria
Actin...teria
2% Actinomycetales
2% Bacteroidetes
1% Firmicutes
24 more

GMB1LS

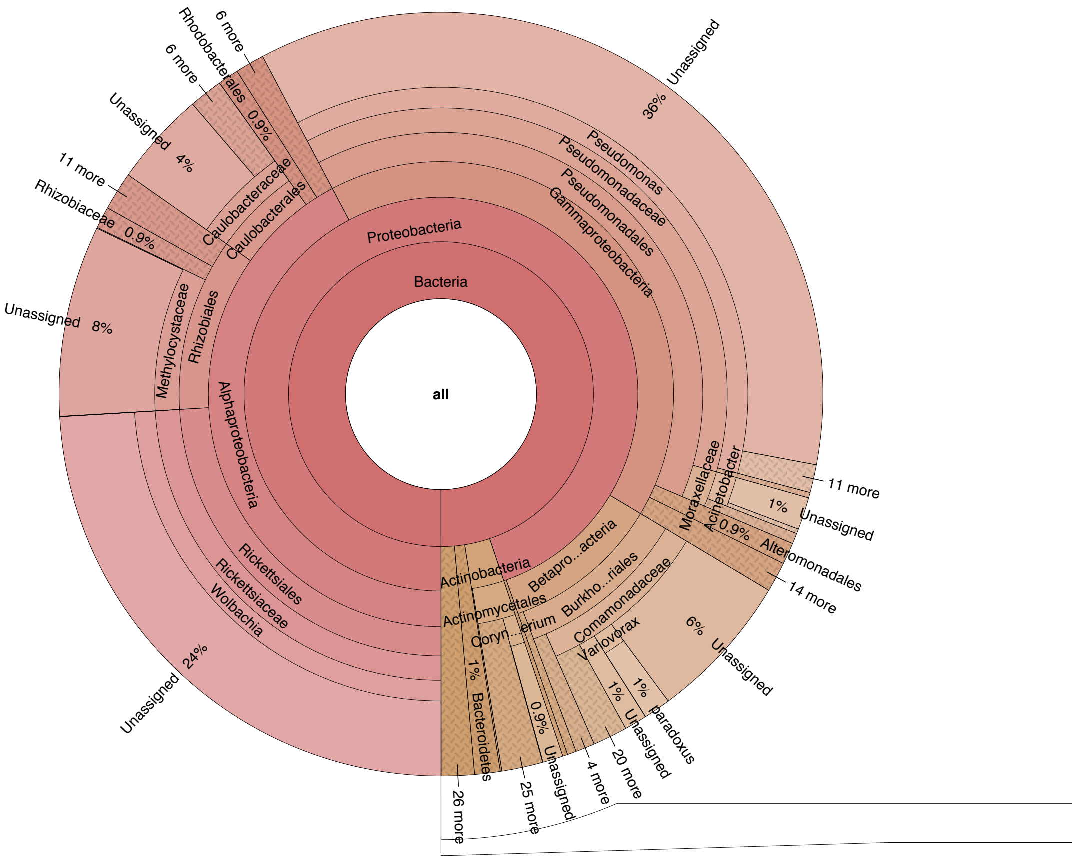
all
Bacteria
Proteobacteria
Gammaproteobacteria
Pseudomonadales
Pseudomonadaceae
Pseudomonas
Unassigned 36%
Moraxellaceae
Acinetobacter
11 more
1% Unassigned
0.9% Alteromonadales
14 more
6% Unassigned
Betapro...acteria
Burkho...riales
Comamonadaceae
Variovorax
1% paradoxus
1% Unassigned
20 more
4 more
Actinobacteria
Actinomycetales
Coryn...erium
0.9% Unassigned
25 more
1% Bacteroidetes
26 more
Alphaproteobacteria
Rickettsiales
Rickettsiaceae
Wolbachia
Unassigned 24%
Rhizobiales
Methylocystaceae
Unassigned 8%
Rhizobiaceae 0.9%
11 more
Caulobacterales
Caulobacteraceae
Unassigned 4%
6 more
Rhodobacterales 0.9%
6 more

GMB1M

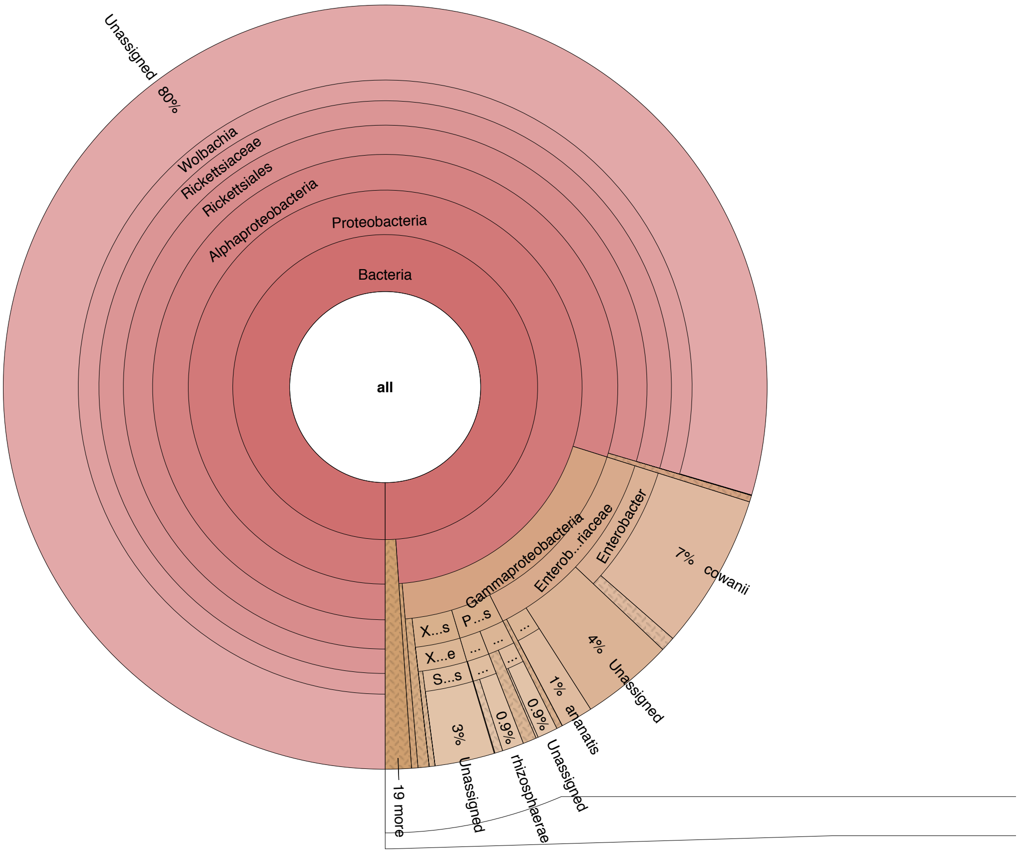

all
Bacteria
Proteobacteria
Alphaproteobacteria
Rickettsiales
Rickettsiaceae
Wolbachia
Unassigned 80%
Gammaproteobacteria
Enterob...riaceae
Enterobacter
7% cowanii
4% Unassigned
1% ananatis
0.9% Unassigned
0.9% rhizosphaerae
3% Unassigned
X...s
P...s
X...e
S...s
19 more

GMB1P

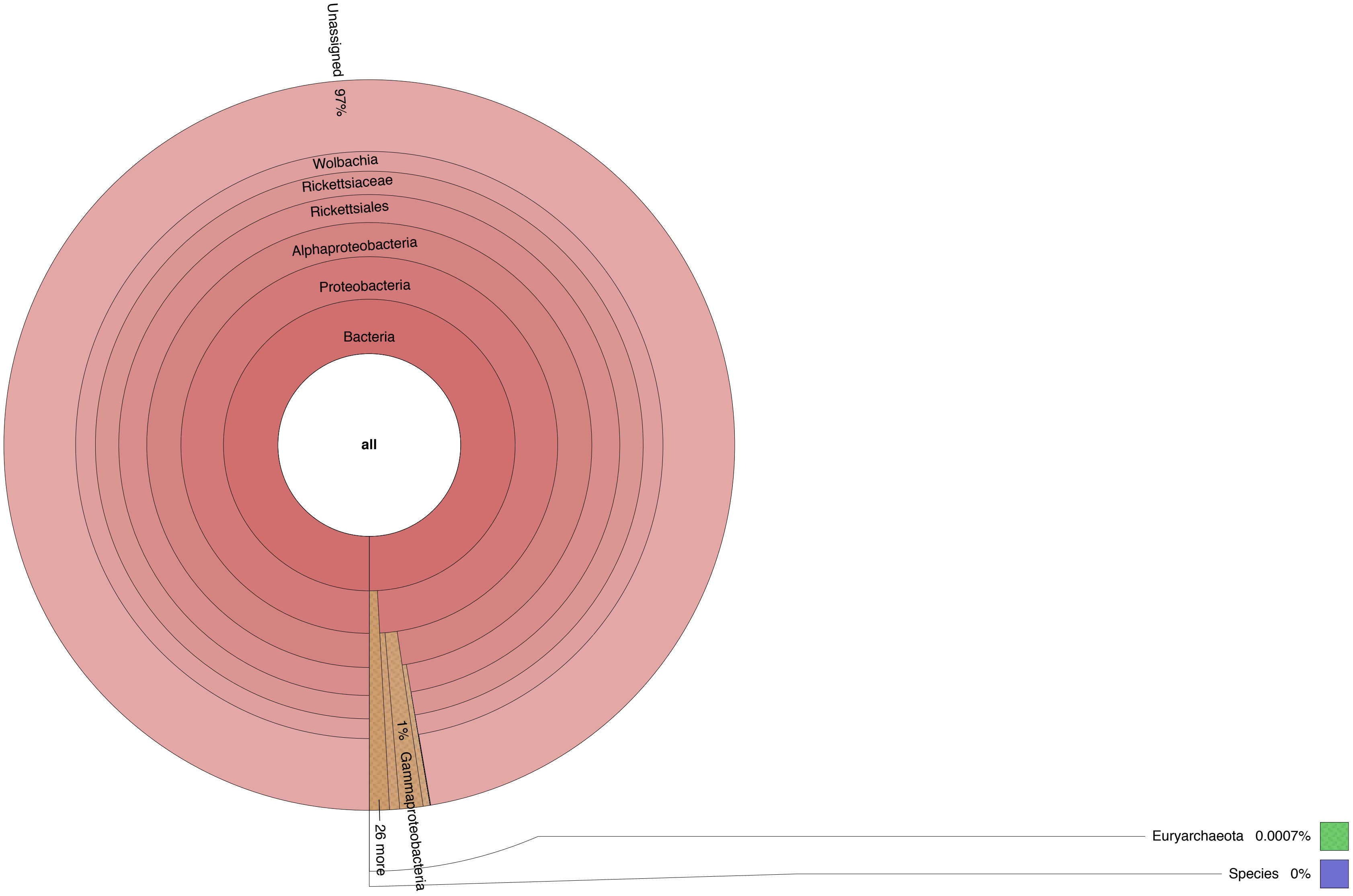

Unassigned 97%
Wolbachia
Rickettsiaceae
Rickettsiales
Alphaproteobacteria
Proteobacteria
Bacteria
all
1% Gammaproteobacteria
26 more
Euryarchaeota 0.0007%
Species 0%

Supplementary data 6| Comparison of bacterial populations between GMB1LR and GMB1LS at phyla level.

		GMB1LR	GMB1LS
Legend	Taxonomy	%	%
	k__Archaea;p__Crenarchaeota	0.0%	0.0%
	k__Archaea;p__Euryarchaeota	0.0%	0.0%
	k__Bacteria;p__Acidobacteria	0.0%	0.0%
	k__Bacteria;p__Actinobacteria	0.7%	1.4%
	k__Bacteria;p__Armatimonadetes	0.0%	0.0%
	k__Bacteria;p__Bacteroidetes	0.5%	0.6%
	k__Bacteria;p__Chlamydiae	0.0%	0.0%
	k__Bacteria;p__Chlorobi	0.0%	0.0%
	k__Bacteria;p__Chloroflexi	0.1%	0.1%
	k__Bacteria;p__Cyanobacteria	34.8%	30.1%
	k__Bacteria;p__Elusimicrobia	0.0%	0.0%
	k__Bacteria;p__Fibrobacteres	0.0%	0.0%
	k__Bacteria;p__Firmicutes	0.4%	0.4%
	k__Bacteria;p__Fusobacteria	0.0%	0.0%
	k__Bacteria;p__GN02	0.0%	0.0%
	k__Bacteria;p__Gemmatimonadetes	0.0%	0.0%
	k__Bacteria;p__NKB19	0.0%	0.0%
	k__Bacteria;p__Nitrospirae	0.0%	0.0%
	k__Bacteria;p__OD1	0.0%	0.0%
	k__Bacteria;p__OP1	0.0%	0.0%
	k__Bacteria;p__Planctomycetes	0.1%	0.0%
	k__Bacteria;p__Proteobacteria	63.2%	67.3%
	k__Bacteria;p__SR1	0.0%	0.0%
	k__Bacteria;p__Spirochaetes	0.0%	0.0%
	k__Bacteria;p__Synergistetes	0.0%	0.0%
	k__Bacteria;p__TM6	0.0%	0.0%
	k__Bacteria;p__TM7	0.0%	0.0%
	k__Bacteria;p__Tenericutes	0.0%	0.0%
	k__Bacteria;p__Thermotogae	0.0%	0.0%
	k__Bacteria;p__Verrucomicrobia	0.0%	0.0%
	k__Bacteria;p__WPS-2	0.0%	0.0%
	k__Bacteria;p__ZB3	0.0%	0.0%
	k__Bacteria;p__[Thermi]	0.0%	0.0%

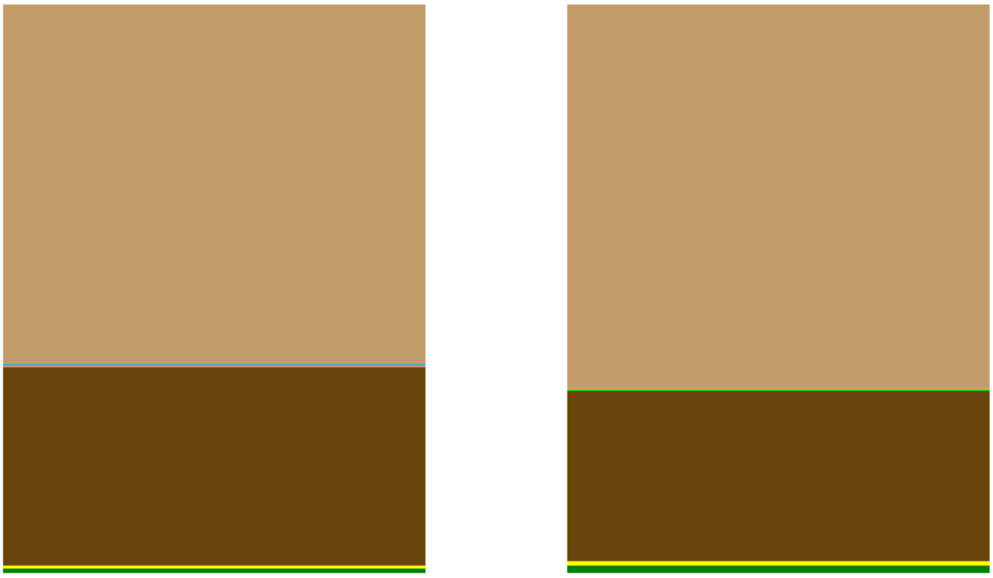

GMB1LR GMB1LS

Comparison of bacterial population at genus level in GMB1LR vs GMB1LS sample

		GMB1LR	GMB1LS
Legend	Taxonomy	%	%
	k__Archaea;p__Crenarchaeota;c__MCG;o__pGrfC26;f__;g__	0.0%	0.0%
	k__Archaea;p__Crenarchaeota;c__Thaumarchaeota;o__Cenarchaeales;f__Cenarchaeaceae;g__	0.0%	0.0%
	k__Archaea;p__Euryarchaeota;c__Halobacteria;o__Halobacteriales;f__Halobacteriaceae;g__Halococcus	0.0%	0.0%
	k__Archaea;p__Euryarchaeota;c__Methanobacteria;o__Methanobacteriales;f__Methanobacteriaceae;g__Methanobacterium	0.0%	0.0%
	k__Archaea;p__Euryarchaeota;c__Methanomicrobia;o__Methanomicrobiales;f__;g__	0.0%	0.0%
	k__Archaea;p__Euryarchaeota;c__Methanomicrobia;o__Methanosarcinales;f__Methanosarcinaceae;g__Methanomethylovorans	0.0%	0.0%
	k__Bacteria;p__Acidobacteria;c__AT-s2-57;o__;f__;g__	0.0%	0.0%
	k__Bacteria;p__Acidobacteria;c__Acidobacteria-6;o__CCU21;f__;g__	0.0%	0.0%
	k__Bacteria;p__Acidobacteria;c__Acidobacteria-6;o__iii1-15;f__;g__	0.0%	0.0%
	k__Bacteria;p__Acidobacteria;c__Acidobacteria-6;o__iii1-15;f__mb2424;g__	0.0%	0.0%
	k__Bacteria;p__Acidobacteria;c__Acidobacteriia;o__Acidobacteriales;f__Koribacteraceae;g__	0.0%	0.0%
	k__Bacteria;p__Acidobacteria;c__Acidobacteriia;o__Acidobacteriales;f__Koribacteraceae;g__Candidatus Koribacter	0.0%	0.0%
	k__Bacteria;p__Acidobacteria;c__BPC102;o__B110;f__;g__	0.0%	0.0%
	k__Bacteria;p__Acidobacteria;c__BPC102;o__MVS-40;f__;g__	0.0%	0.0%
	k__Bacteria;p__Acidobacteria;c__DA052;o__Ellin6513;f__;g__	0.0%	0.0%
	k__Bacteria;p__Acidobacteria;c__Solibacteres;o__Solibacterales;f__;g__	0.0%	0.0%
	k__Bacteria;p__Acidobacteria;c__Solibacteres;o__Solibacterales;f__Solibacteraceae;g__	0.0%	0.0%
	k__Bacteria;p__Acidobacteria;c__Solibacteres;o__Solibacterales;f__Solibacteraceae;g__Candidatus Solibacter	0.0%	0.0%
	k__Bacteria;p__Acidobacteria;c__Solibacteres;o__Solibacterales;f__[Bryobacteraceae];g__	0.0%	0.0%
	k__Bacteria;p__Acidobacteria;c__Sva0725;o__Sva0725;f__;g__	0.0%	0.0%
	k__Bacteria;p__Acidobacteria;c__[Chloracidobacteria];o__PK29;f__;g__	0.0%	0.0%
	k__Bacteria;p__Acidobacteria;c__[Chloracidobacteria];o__RB41;f__;g__	0.0%	0.0%
	k__Bacteria;p__Acidobacteria;c__[Chloracidobacteria];o__RB41;f__Ellin6075;g__	0.0%	0.0%
	k__Bacteria;p__Acidobacteria;c__iii1-8;o__32-20;f__;g__	0.0%	0.0%
	k__Bacteria;p__Acidobacteria;c__iii1-8;o__DS-18;f__;g__	0.0%	0.0%
	k__Bacteria;p__Acidobacteria;c__iii1-8;o__SJA-36;f__;g__	0.0%	0.0%
	k__Bacteria;p__Actinobacteria;c__Acidimicrobiia;o__Acidimicrobiales;f__;g__	0.0%	0.0%
	k__Bacteria;p__Actinobacteria;c__Acidimicrobiia;o__Acidimicrobiales;f__AKIW874;g__	0.0%	0.0%
	k__Bacteria;p__Actinobacteria;c__Acidimicrobiia;o__Acidimicrobiales;f__C111;g__	0.0%	0.0%
	k__Bacteria;p__Actinobacteria;c__Acidimicrobiia;o__Acidimicrobiales;f__Microthrixaceae;g__	0.0%	0.0%
	k__Bacteria;p__Actinobacteria;c__Actinobacteria;o__;f__;g__	0.0%	0.0%
	k__Bacteria;p__Actinobacteria;c__Actinobacteria;o__Actinomycetales;f__;g__	0.0%	0.0%
	k__Bacteria;p__Actinobacteria;c__Actinobacteria;o__Actinomycetales;f__Actinomycetaceae;g__Actinomyces	0.0%	0.0%
	k__Bacteria;p__Actinobacteria;c__Actinobacteria;o__Actinomycetales;f__Actinomycetaceae;g__Varibaculum	0.0%	0.0%
	k__Bacteria;p__Actinobacteria;c__Actinobacteria;o__Actinomycetales;f__Bogoriellaceae;g__Georgenia	0.0%	0.0%
	k__Bacteria;p__Actinobacteria;c__Actinobacteria;o__Actinomycetales;f__Brevibacteriaceae;g__Brevibacterium	0.0%	0.0%
	k__Bacteria;p__Actinobacteria;c__Actinobacteria;o__Actinomycetales;f__Cellulomonadaceae;g__Actinotalea	0.0%	0.0%
	k__Bacteria;p__Actinobacteria;c__Actinobacteria;o__Actinomycetales;f__Cellulomonadaceae;g__Cellulomonas	0.0%	0.0%
	k__Bacteria;p__Actinobacteria;c__Actinobacteria;o__Actinomycetales;f__Cellulomonadaceae;g__Demequina	0.0%	0.0%
	k__Bacteria;p__Actinobacteria;c__Actinobacteria;o__Actinomycetales;f__Corynebacteriaceae;g__Corynebacterium	0.2%	0.5%
	k__Bacteria;p__Actinobacteria;c__Actinobacteria;o__Actinomycetales;f__Dermabacteraceae;g__	0.0%	0.0%
	k__Bacteria;p__Actinobacteria;c__Actinobacteria;o__Actinomycetales;f__Dermabacteraceae;g__Brachybacterium	0.0%	0.0%
	k__Bacteria;p__Actinobacteria;c__Actinobacteria;o__Actinomycetales;f__Dermabacteraceae;g__Helcobacillus	0.0%	0.0%
	k__Bacteria;p__Actinobacteria;c__Actinobacteria;o__Actinomycetales;f__Dermacoccaceae;g__Dermacoccus	0.0%	0.0%
	k__Bacteria;p__Actinobacteria;c__Actinobacteria;o__Actinomycetales;f__Dermatophilaceae;g__Piscicoccus	0.0%	0.0%
	k__Bacteria;p__Actinobacteria;c__Actinobacteria;o__Actinomycetales;f__Dietziaceae;g__Dietzia	0.0%	0.0%
	k__Bacteria;p__Actinobacteria;c__Actinobacteria;o__Actinomycetales;f__Geodermatophilaceae;g__	0.0%	0.0%
	k__Bacteria;p__Actinobacteria;c__Actinobacteria;o__Actinomycetales;f__Geodermatophilaceae;g__Blastococcus	0.0%	0.0%
	k__Bacteria;p__Actinobacteria;c__Actinobacteria;o__Actinomycetales;f__Geodermatophilaceae;g__Geodermatophilus	0.0%	0.0%
	k__Bacteria;p__Actinobacteria;c__Actinobacteria;o__Actinomycetales;f__Geodermatophilaceae;g__Modestobacter	0.0%	0.0%
	k__Bacteria;p__Actinobacteria;c__Actinobacteria;o__Actinomycetales;f__Gordoniaceae;g__Gordonia	0.0%	0.0%
	k__Bacteria;p__Actinobacteria;c__Actinobacteria;o__Actinomycetales;f__Gordoniaceae;g__Millisia	0.0%	0.0%
	k__Bacteria;p__Actinobacteria;c__Actinobacteria;o__Actinomycetales;f__Intrasporangiaceae;g__	0.0%	0.0%

	k__Bacteria;p__Actinobacteria;c__Actinobacteria;o__Actinomycetales;f__Intrasporangiaceae;g__Knoellia	0.0%	0.0%
	k__Bacteria;p__Actinobacteria;c__Actinobacteria;o__Actinomycetales;f__Intrasporangiaceae;g__Kytococcus	0.0%	0.0%
	k__Bacteria;p__Actinobacteria;c__Actinobacteria;o__Actinomycetales;f__Intrasporangiaceae;g__Phycicoccus	0.0%	0.0%
	k__Bacteria;p__Actinobacteria;c__Actinobacteria;o__Actinomycetales;f__Intrasporangiaceae;g__Serinicoccus	0.0%	0.0%
	k__Bacteria;p__Actinobacteria;c__Actinobacteria;o__Actinomycetales;f__Jonesiaceae;g__Jonesia	0.0%	0.0%
	k__Bacteria;p__Actinobacteria;c__Actinobacteria;o__Actinomycetales;f__Kineosporiaceae;g__	0.0%	0.0%
	k__Bacteria;p__Actinobacteria;c__Actinobacteria;o__Actinomycetales;f__Microbacteriaceae;g__	0.0%	0.0%
	k__Bacteria;p__Actinobacteria;c__Actinobacteria;o__Actinomycetales;f__Microbacteriaceae;g__Agrococcus	0.0%	0.0%
	k__Bacteria;p__Actinobacteria;c__Actinobacteria;o__Actinomycetales;f__Microbacteriaceae;g__Agromyces	0.0%	0.0%
	k__Bacteria;p__Actinobacteria;c__Actinobacteria;o__Actinomycetales;f__Microbacteriaceae;g__Cryobacterium	0.0%	0.0%
	k__Bacteria;p__Actinobacteria;c__Actinobacteria;o__Actinomycetales;f__Microbacteriaceae;g__Curtobacterium	0.0%	0.0%
	k__Bacteria;p__Actinobacteria;c__Actinobacteria;o__Actinomycetales;f__Microbacteriaceae;g__Frigoribacterium	0.0%	0.0%
	k__Bacteria;p__Actinobacteria;c__Actinobacteria;o__Actinomycetales;f__Microbacteriaceae;g__Herbiconiux	0.0%	0.0%
	k__Bacteria;p__Actinobacteria;c__Actinobacteria;o__Actinomycetales;f__Microbacteriaceae;g__Leifsonia	0.0%	0.0%
	k__Bacteria;p__Actinobacteria;c__Actinobacteria;o__Actinomycetales;f__Microbacteriaceae;g__Leucobacter	0.0%	0.0%
	k__Bacteria;p__Actinobacteria;c__Actinobacteria;o__Actinomycetales;f__Microbacteriaceae;g__Microbacterium	0.0%	0.0%
	k__Bacteria;p__Actinobacteria;c__Actinobacteria;o__Actinomycetales;f__Microbacteriaceae;g__Pseudoclavibacter	0.0%	0.0%
	k__Bacteria;p__Actinobacteria;c__Actinobacteria;o__Actinomycetales;f__Microbacteriaceae;g__Rathayibacter	0.0%	0.0%
	k__Bacteria;p__Actinobacteria;c__Actinobacteria;o__Actinomycetales;f__Microbacteriaceae;g__Salinibacterium	0.0%	0.0%
	k__Bacteria;p__Actinobacteria;c__Actinobacteria;o__Actinomycetales;f__Microbacteriaceae;g__Yonghaparkia	0.0%	0.0%
	k__Bacteria;p__Actinobacteria;c__Actinobacteria;o__Actinomycetales;f__Micrococcaceae;g__	0.0%	0.0%
	k__Bacteria;p__Actinobacteria;c__Actinobacteria;o__Actinomycetales;f__Micrococcaceae;g__Arthrobacter	0.0%	0.0%
	k__Bacteria;p__Actinobacteria;c__Actinobacteria;o__Actinomycetales;f__Micrococcaceae;g__Citricoccus	0.0%	0.1%
	k__Bacteria;p__Actinobacteria;c__Actinobacteria;o__Actinomycetales;f__Micrococcaceae;g__Kocuria	0.0%	0.1%
	k__Bacteria;p__Actinobacteria;c__Actinobacteria;o__Actinomycetales;f__Micrococcaceae;g__Microbispora	0.0%	0.0%
	k__Bacteria;p__Actinobacteria;c__Actinobacteria;o__Actinomycetales;f__Micrococcaceae;g__Micrococcus	0.0%	0.0%
	k__Bacteria;p__Actinobacteria;c__Actinobacteria;o__Actinomycetales;f__Micrococcaceae;g__Nesterenkonia	0.0%	0.1%
	k__Bacteria;p__Actinobacteria;c__Actinobacteria;o__Actinomycetales;f__Micrococcaceae;g__Renibacterium	0.0%	0.0%
	k__Bacteria;p__Actinobacteria;c__Actinobacteria;o__Actinomycetales;f__Micrococcaceae;g__Rothia	0.0%	0.0%
	k__Bacteria;p__Actinobacteria;c__Actinobacteria;o__Actinomycetales;f__Micromonosporaceae;g__	0.0%	0.0%
	k__Bacteria;p__Actinobacteria;c__Actinobacteria;o__Actinomycetales;f__Micromonosporaceae;g__Actinoplanes	0.0%	0.0%
	k__Bacteria;p__Actinobacteria;c__Actinobacteria;o__Actinomycetales;f__Mycobacteriaceae;g__Mycobacterium	0.0%	0.0%
	k__Bacteria;p__Actinobacteria;c__Actinobacteria;o__Actinomycetales;f__Nocardiaceae;g__	0.0%	0.0%
	k__Bacteria;p__Actinobacteria;c__Actinobacteria;o__Actinomycetales;f__Nocardiaceae;g__Nocardia	0.0%	0.0%
	k__Bacteria;p__Actinobacteria;c__Actinobacteria;o__Actinomycetales;f__Nocardiaceae;g__Rhodococcus	0.0%	0.1%
	k__Bacteria;p__Actinobacteria;c__Actinobacteria;o__Actinomycetales;f__Nocardioidaceae;g__	0.0%	0.0%
	k__Bacteria;p__Actinobacteria;c__Actinobacteria;o__Actinomycetales;f__Nocardioidaceae;g__Aeromicrobium	0.0%	0.0%
	k__Bacteria;p__Actinobacteria;c__Actinobacteria;o__Actinomycetales;f__Nocardioidaceae;g__Friedmanniella	0.0%	0.0%
	k__Bacteria;p__Actinobacteria;c__Actinobacteria;o__Actinomycetales;f__Nocardioidaceae;g__Nocardioides	0.0%	0.0%
	k__Bacteria;p__Actinobacteria;c__Actinobacteria;o__Actinomycetales;f__Nocardioidaceae;g__Pimelobacter	0.0%	0.0%
	k__Bacteria;p__Actinobacteria;c__Actinobacteria;o__Actinomycetales;f__Nocardioidaceae;g__Propionicimonas	0.0%	0.0%
	k__Bacteria;p__Actinobacteria;c__Actinobacteria;o__Actinomycetales;f__Promicromonosporaceae;g__Cellulosimicrobium	0.0%	0.0%
	k__Bacteria;p__Actinobacteria;c__Actinobacteria;o__Actinomycetales;f__Propionibacteriaceae;g__	0.0%	0.0%
	k__Bacteria;p__Actinobacteria;c__Actinobacteria;o__Actinomycetales;f__Propionibacteriaceae;g__Propionibacterium	0.0%	0.2%
	k__Bacteria;p__Actinobacteria;c__Actinobacteria;o__Actinomycetales;f__Propionibacteriaceae;g__Tessaracoccus	0.0%	0.0%
	k__Bacteria;p__Actinobacteria;c__Actinobacteria;o__Actinomycetales;f__Pseudonocardiaceae;g__Actinomycetospora	0.0%	0.0%
	k__Bacteria;p__Actinobacteria;c__Actinobacteria;o__Actinomycetales;f__Pseudonocardiaceae;g__Amycolatopsis	0.0%	0.0%
	k__Bacteria;p__Actinobacteria;c__Actinobacteria;o__Actinomycetales;f__Pseudonocardiaceae;g__Pseudonocardia	0.0%	0.0%
	k__Bacteria;p__Actinobacteria;c__Actinobacteria;o__Actinomycetales;f__Pseudonocardiaceae;g__Saccharopolyspora	0.0%	0.0%
	k__Bacteria;p__Actinobacteria;c__Actinobacteria;o__Actinomycetales;f__Sanguibacteraceae;g__Sanguibacter	0.0%	0.0%
	k__Bacteria;p__Actinobacteria;c__Actinobacteria;o__Actinomycetales;f__Sporichthyaceae;g__	0.0%	0.0%
	k__Bacteria;p__Actinobacteria;c__Actinobacteria;o__Actinomycetales;f__Streptomycetaceae;g__	0.0%	0.0%
	k__Bacteria;p__Actinobacteria;c__Actinobacteria;o__Actinomycetales;f__Streptomycetaceae;g__Amycolatopsis	0.0%	0.0%
	k__Bacteria;p__Actinobacteria;c__Actinobacteria;o__Actinomycetales;f__Streptomycetaceae;g__Streptomyces	0.0%	0.0%
	k__Bacteria;p__Actinobacteria;c__Actinobacteria;o__Actinomycetales;f__Thermomonosporaceae;g__Actinocorallia	0.0%	0.0%
	k__Bacteria;p__Actinobacteria;c__Actinobacteria;o__Actinomycetales;f__Yaniellaceae;g__Yaniella	0.0%	0.0%
	k__Bacteria;p__Actinobacteria;c__Coriobacteriia;o__Coriobacteriales;f__Coriobacteriaceae;g__	0.0%	0.0%
	k__Bacteria;p__Actinobacteria;c__MB-A2-108;o__;f__;g__	0.0%	0.0%
	k__Bacteria;p__Actinobacteria;c__Nitriliruptoria;o__Euzebyales;f__Euzebyaceae;g__Euzebya	0.0%	0.0%

	k__Bacteria;p__Actinobacteria;c__OPB41;o__;f__;g__	0.0%	0.0%
	k__Bacteria;p__Actinobacteria;c__Rubrobacteria;o__Rubrobacterales;f__Rubrobacteraceae;g__	0.0%	0.0%
	k__Bacteria;p__Actinobacteria;c__Rubrobacteria;o__Rubrobacterales;f__Rubrobacteraceae;g__Rubrobacter	0.0%	0.0%
	k__Bacteria;p__Actinobacteria;c__Thermoleophilia;o__Gaiellales;f__;g__	0.0%	0.0%
	k__Bacteria;p__Actinobacteria;c__Thermoleophilia;o__Gaiellales;f__Gaiellaceae;g__	0.0%	0.0%
	k__Bacteria;p__Actinobacteria;c__Thermoleophilia;o__Solirubrobacterales;f__;g__	0.0%	0.0%
	k__Bacteria;p__Actinobacteria;c__Thermoleophilia;o__Solirubrobacterales;f__Solirubrobacteraceae;g__	0.0%	0.0%
	k__Bacteria;p__Armatimonadetes;c__[Fimbriimonadia];o__[Fimbriimonadales];f__[Fimbriimonadaceae];g__Fimbriimonas	0.0%	0.0%
	k__Bacteria;p__Bacteroidetes;c__;o__;f__;g__	0.0%	0.0%
	k__Bacteria;p__Bacteroidetes;c__Bacteroidia;o__Bacteroidales;f__;g__	0.0%	0.0%
	k__Bacteria;p__Bacteroidetes;c__Bacteroidia;o__Bacteroidales;f__BA008;g__	0.0%	0.0%
	k__Bacteria;p__Bacteroidetes;c__Bacteroidia;o__Bacteroidales;f__Bacteroidaceae;g__Bacteroides	0.0%	0.0%
	k__Bacteria;p__Bacteroidetes;c__Bacteroidia;o__Bacteroidales;f__Marinilabiaceae;g__	0.0%	0.0%
	k__Bacteria;p__Bacteroidetes;c__Bacteroidia;o__Bacteroidales;f__Porphyromonadaceae;g__	0.0%	0.0%
	k__Bacteria;p__Bacteroidetes;c__Bacteroidia;o__Bacteroidales;f__Porphyromonadaceae;g__Dysgonomonas	0.0%	0.0%
	k__Bacteria;p__Bacteroidetes;c__Bacteroidia;o__Bacteroidales;f__Porphyromonadaceae;g__Paludibacter	0.0%	0.0%
	k__Bacteria;p__Bacteroidetes;c__Bacteroidia;o__Bacteroidales;f__Porphyromonadaceae;g__Porphyromonas	0.0%	0.0%
	k__Bacteria;p__Bacteroidetes;c__Bacteroidia;o__Bacteroidales;f__Prevotellaceae;g__Prevotella	0.0%	0.0%
	k__Bacteria;p__Bacteroidetes;c__Bacteroidia;o__Bacteroidales;f__RF16;g__	0.0%	0.0%
	k__Bacteria;p__Bacteroidetes;c__Bacteroidia;o__Bacteroidales;f__S24-7;g__	0.0%	0.0%
	k__Bacteria;p__Bacteroidetes;c__Bacteroidia;o__Bacteroidales;f__SB-1;g__	0.0%	0.0%
	k__Bacteria;p__Bacteroidetes;c__Bacteroidia;o__Bacteroidales;f__[Paraprevotellaceae];g__CF231	0.0%	0.0%
	k__Bacteria;p__Bacteroidetes;c__Bacteroidia;o__Bacteroidales;f__[Paraprevotellaceae];g__[Prevotella]	0.0%	0.0%
	k__Bacteria;p__Bacteroidetes;c__Cytophagia;o__Cytophagales;f__;g__	0.0%	0.0%
	k__Bacteria;p__Bacteroidetes;c__Cytophagia;o__Cytophagales;f__Cyclobacteriaceae;g__	0.0%	0.0%
	k__Bacteria;p__Bacteroidetes;c__Cytophagia;o__Cytophagales;f__Cytophagaceae;g__	0.0%	0.0%
	k__Bacteria;p__Bacteroidetes;c__Cytophagia;o__Cytophagales;f__Cytophagaceae;g__Dyadobacter	0.0%	0.0%
	k__Bacteria;p__Bacteroidetes;c__Cytophagia;o__Cytophagales;f__Cytophagaceae;g__Emticicia	0.0%	0.0%
	k__Bacteria;p__Bacteroidetes;c__Cytophagia;o__Cytophagales;f__Cytophagaceae;g__Flectobacillus	0.0%	0.0%
	k__Bacteria;p__Bacteroidetes;c__Cytophagia;o__Cytophagales;f__Cytophagaceae;g__Leadbetterella	0.0%	0.0%
	k__Bacteria;p__Bacteroidetes;c__Cytophagia;o__Cytophagales;f__Cytophagaceae;g__Pontibacter	0.0%	0.0%
	k__Bacteria;p__Bacteroidetes;c__Cytophagia;o__Cytophagales;f__Cytophagaceae;g__Runella	0.0%	0.0%
	k__Bacteria;p__Bacteroidetes;c__Cytophagia;o__Cytophagales;f__Cytophagaceae;g__Spirosoma	0.0%	0.0%
	k__Bacteria;p__Bacteroidetes;c__Cytophagia;o__Cytophagales;f__Flammeovirgaceae;g__	0.0%	0.0%
	k__Bacteria;p__Bacteroidetes;c__Cytophagia;o__Cytophagales;f__[Amoebophilaceae];g__Candidatus Amoebophilus	0.0%	0.0%
	k__Bacteria;p__Bacteroidetes;c__Flavobacteriia;o__Flavobacteriales;f__Cryomorphaceae;g__Fluviicola	0.0%	0.0%
	k__Bacteria;p__Bacteroidetes;c__Flavobacteriia;o__Flavobacteriales;f__Flavobacteriaceae;g__	0.0%	0.0%
	k__Bacteria;p__Bacteroidetes;c__Flavobacteriia;o__Flavobacteriales;f__Flavobacteriaceae;g__Flavobacterium	0.1%	0.1%
	k__Bacteria;p__Bacteroidetes;c__Flavobacteriia;o__Flavobacteriales;f__Flavobacteriaceae;g__Gelidibacter	0.0%	0.0%
	k__Bacteria;p__Bacteroidetes;c__Flavobacteriia;o__Flavobacteriales;f__Flavobacteriaceae;g__Gillisia	0.0%	0.0%
	k__Bacteria;p__Bacteroidetes;c__Flavobacteriia;o__Flavobacteriales;f__Flavobacteriaceae;g__Myroides	0.0%	0.0%
	k__Bacteria;p__Bacteroidetes;c__Flavobacteriia;o__Flavobacteriales;f__Flavobacteriaceae;g__Salinimicrobium	0.0%	0.0%
	k__Bacteria;p__Bacteroidetes;c__Flavobacteriia;o__Flavobacteriales;f__Flavobacteriaceae;g__Sediminibacter	0.0%	0.0%
	k__Bacteria;p__Bacteroidetes;c__Flavobacteriia;o__Flavobacteriales;f__[Weeksellaceae];g__	0.0%	0.0%
	k__Bacteria;p__Bacteroidetes;c__Flavobacteriia;o__Flavobacteriales;f__[Weeksellaceae];g__Chryseobacterium	0.0%	0.0%
	k__Bacteria;p__Bacteroidetes;c__Flavobacteriia;o__Flavobacteriales;f__[Weeksellaceae];g__Cloacibacterium	0.0%	0.0%
	k__Bacteria;p__Bacteroidetes;c__Flavobacteriia;o__Flavobacteriales;f__[Weeksellaceae];g__Wautersiella	0.0%	0.0%
	k__Bacteria;p__Bacteroidetes;c__SM1A07;o__;f__;g__	0.0%	0.0%
	k__Bacteria;p__Bacteroidetes;c__Sphingobacteriia;o__Sphingobacteriales;f__;g__	0.0%	0.0%
	k__Bacteria;p__Bacteroidetes;c__Sphingobacteriia;o__Sphingobacteriales;f__Sphingobacteriaceae;g__	0.0%	0.0%
	k__Bacteria;p__Bacteroidetes;c__Sphingobacteriia;o__Sphingobacteriales;f__Sphingobacteriaceae;g__Mucilaginibacter	0.0%	0.0%
	k__Bacteria;p__Bacteroidetes;c__Sphingobacteriia;o__Sphingobacteriales;f__Sphingobacteriaceae;g__Pedobacter	0.0%	0.0%
	k__Bacteria;p__Bacteroidetes;c__Sphingobacteriia;o__Sphingobacteriales;f__Sphingobacteriaceae;g__Sphingobacterium	0.0%	0.0%
	k__Bacteria;p__Bacteroidetes;c__[Rhodothermi];o__[Rhodothermales];f__Rhodothermaceae;g__Rhodothermus	0.0%	0.0%
	k__Bacteria;p__Bacteroidetes;c__[Saprospirae];o__[Saprospirales];f__;g__	0.0%	0.0%
	k__Bacteria;p__Bacteroidetes;c__[Saprospirae];o__[Saprospirales];f__Chitinophagaceae;g__	0.0%	0.0%
	k__Bacteria;p__Bacteroidetes;c__[Saprospirae];o__[Saprospirales];f__Chitinophagaceae;g__Chitinophaga	0.0%	0.0%
	k__Bacteria;p__Bacteroidetes;c__[Saprospirae];o__[Saprospirales];f__Chitinophagaceae;g__Flavihumibacter	0.0%	0.0%
	k__Bacteria;p__Bacteroidetes;c__[Saprospirae];o__[Saprospirales];f__Chitinophagaceae;g__Flavisolibacter	0.0%	0.0%

k__Bacteria;p__Bacteroidetes;c__[Saprospirae];o__[Saprospirales];f__Chitinophagaceae;g__Lacibacter	0.0%	0.0%
k__Bacteria;p__Bacteroidetes;c__[Saprospirae];o__[Saprospirales];f__Chitinophagaceae;g__Niabella	0.0%	0.0%
k__Bacteria;p__Bacteroidetes;c__[Saprospirae];o__[Saprospirales];f__Chitinophagaceae;g__Parasegitibacter	0.0%	0.0%
k__Bacteria;p__Bacteroidetes;c__[Saprospirae];o__[Saprospirales];f__Chitinophagaceae;g__Sediminibacterium	0.2%	0.2%
k__Bacteria;p__Bacteroidetes;c__[Saprospirae];o__[Saprospirales];f__Saprospiraceae;g__	0.0%	0.0%
k__Bacteria;p__Bacteroidetes;c__[Saprospirae];o__[Saprospirales];f__Saprospiraceae;g__Haliscomenobacter	0.0%	0.0%
k__Bacteria;p__Chlamydiae;c__Chlamydiia;o__Chlamydiales;f__Criblamydiaceae;g__	0.0%	0.0%
k__Bacteria;p__Chlorobi;c__BSV26;o__C20;f__;g__	0.0%	0.0%
k__Bacteria;p__Chlorobi;c__Ignavibacteria;o__Ignavibacteriales;f__Ignavibacteriaceae;g__	0.0%	0.0%
k__Bacteria;p__Chlorobi;c__OPB56;o__;f__;g__	0.0%	0.0%
k__Bacteria;p__Chloroflexi;c__Anaerolineae;o__Anaerolineales;f__Anaerolinaceae;g__	0.0%	0.0%
k__Bacteria;p__Chloroflexi;c__Anaerolineae;o__Anaerolineales;f__Anaerolinaceae;g__Anaerolinea	0.0%	0.0%
k__Bacteria;p__Chloroflexi;c__Anaerolineae;o__Anaerolineales;f__Anaerolinaceae;g__C1_B004	0.0%	0.0%
k__Bacteria;p__Chloroflexi;c__Anaerolineae;o__Anaerolineales;f__Anaerolinaceae;g__SHD-14	0.0%	0.0%
k__Bacteria;p__Chloroflexi;c__Anaerolineae;o__Anaerolineales;f__Anaerolinaceae;g__SHD-231	0.0%	0.0%
k__Bacteria;p__Chloroflexi;c__Anaerolineae;o__Anaerolineales;f__Anaerolinaceae;g__WCHB1-05	0.0%	0.0%
k__Bacteria;p__Chloroflexi;c__Anaerolineae;o__Caldilineales;f__Caldilineaceae;g__	0.0%	0.0%
k__Bacteria;p__Chloroflexi;c__Anaerolineae;o__GCA004;f__;g__	0.0%	0.0%
k__Bacteria;p__Chloroflexi;c__Anaerolineae;o__S0208;f__;g__	0.0%	0.0%
k__Bacteria;p__Chloroflexi;c__Anaerolineae;o__SB-34;f__;g__	0.0%	0.0%
k__Bacteria;p__Chloroflexi;c__Anaerolineae;o__SBR1031;f__A4b;g__	0.0%	0.0%
k__Bacteria;p__Chloroflexi;c__Anaerolineae;o__SBR1031;f__SHA-31;g__	0.0%	0.0%
k__Bacteria;p__Chloroflexi;c__Anaerolineae;o__SBR1031;f__SJA-101;g__	0.0%	0.0%
k__Bacteria;p__Chloroflexi;c__Anaerolineae;o__SBR1031;f__oc28;g__	0.0%	0.0%
k__Bacteria;p__Chloroflexi;c__Anaerolineae;o__SJA-15;f__;g__	0.0%	0.0%
k__Bacteria;p__Chloroflexi;c__Anaerolineae;o__envOPS12;f__;g__	0.0%	0.0%
k__Bacteria;p__Chloroflexi;c__Chloroflexi;o__Chloroflexales;f__Chloroflexaceae;g__	0.0%	0.0%
k__Bacteria;p__Chloroflexi;c__Chloroflexi;o__Chloroflexales;f__Chloroflexaceae;g__Chloroflexus	0.0%	0.0%
k__Bacteria;p__Chloroflexi;c__Chloroflexi;o__Herpetosiphonales;f__;g__	0.0%	0.0%
k__Bacteria;p__Chloroflexi;c__Ellin6529;o__;f__;g__	0.0%	0.0%
k__Bacteria;p__Chloroflexi;c__Gitt-GS-136;o__;f__;g__	0.0%	0.0%
k__Bacteria;p__Chloroflexi;c__Ktedonobacteria;o__Thermogemmatisporales;f__Thermogemmatisporaceae;g__	0.0%	0.0%
k__Bacteria;p__Chloroflexi;c__S085;o__;f__;g__	0.0%	0.0%
k__Bacteria;p__Chloroflexi;c__TK10;o__AKYG885;f__Dolo_23;g__	0.0%	0.0%
k__Bacteria;p__Chloroflexi;c__TK10;o__B07_WMSP1;f__;g__	0.0%	0.0%
k__Bacteria;p__Chloroflexi;c__TK17;o__;f__;g__	0.0%	0.0%
k__Bacteria;p__Chloroflexi;c__Thermomicrobia;o__AKYG1722;f__;g__	0.0%	0.0%
k__Bacteria;p__Chloroflexi;c__Thermomicrobia;o__JG30-KF-CM45;f__;g__	0.0%	0.0%
k__Bacteria;p__Cyanobacteria;c__4C0d-2;o__MLE1-12;f__;g__	0.0%	0.0%
k__Bacteria;p__Cyanobacteria;c__Chloroplast;o__;f__;g__	0.0%	0.0%
k__Bacteria;p__Cyanobacteria;c__Chloroplast;o__Chlorophyta;f__;g__	0.0%	0.0%
k__Bacteria;p__Cyanobacteria;c__Chloroplast;o__Chlorophyta;f__Trebouxiophyceae;g__	0.0%	0.0%
k__Bacteria;p__Cyanobacteria;c__Chloroplast;o__Chlorophyta;f__Ulvophyceae;g__	0.0%	0.0%
k__Bacteria;p__Cyanobacteria;c__Chloroplast;o__Stramenopiles;f__;g__	0.0%	0.0%
k__Bacteria;p__Cyanobacteria;c__Chloroplast;o__Streptophyta;f__;g__	34.8%	30.0%
k__Bacteria;p__Cyanobacteria;c__Gloeobacterophycideae;o__Gloeobacterales;f__Gloeobacteraceae;g__Gloeobacter	0.0%	0.0%
k__Bacteria;p__Cyanobacteria;c__ML635J-21;o__;f__;g__	0.0%	0.0%
k__Bacteria;p__Cyanobacteria;c__Nostocophycideae;o__Nostocales;f__Nostocaceae;g__Nostoc	0.0%	0.0%
k__Bacteria;p__Cyanobacteria;c__Nostocophycideae;o__Stigonematales;f__Rivulariaceae;g__Calothrix	0.0%	0.0%
k__Bacteria;p__Cyanobacteria;c__Oscillatoriophycideae;o__Chroococcales;f__;g__	0.0%	0.0%
k__Bacteria;p__Cyanobacteria;c__Oscillatoriophycideae;o__Chroococcales;f__Cyanobacteriaceae;g__Rubidibacter	0.0%	0.0%
k__Bacteria;p__Cyanobacteria;c__Oscillatoriophycideae;o__Chroococcales;f__Spirulinaceae;g__Spirulina	0.0%	0.0%
k__Bacteria;p__Cyanobacteria;c__Oscillatoriophycideae;o__Chroococcales;f__Xenococcaceae;g__	0.0%	0.0%
k__Bacteria;p__Cyanobacteria;c__Oscillatoriophycideae;o__Chroococcales;f__Xenococcaceae;g__Gloeocapsopsis	0.0%	0.0%
k__Bacteria;p__Cyanobacteria;c__Oscillatoriophycideae;o__Oscillatoriales;f__Phormidiaceae;g__	0.0%	0.0%
k__Bacteria;p__Cyanobacteria;c__Oscillatoriophycideae;o__Oscillatoriales;f__Phormidiaceae;g__Phormidium	0.0%	0.0%
k__Bacteria;p__Cyanobacteria;c__Oscillatoriophycideae;o__Oscillatoriales;f__Phormidiaceae;g__Planktothrix	0.0%	0.0%
k__Bacteria;p__Cyanobacteria;c__Synechococcophycideae;o__Pseudanabaenales;f__;g__	0.0%	0.0%
k__Bacteria;p__Cyanobacteria;c__Synechococcophycideae;o__Pseudanabaenales;f__Pseudanabaenaceae;g__	0.0%	0.0%

	k__Bacteria;p__Cyanobacteria;c__Synechococcophycideae;o__Pseudanabaenales;f__Pseudanabaenaceae;g__Leptolyngbya	0.0%	0.0%
	k__Bacteria;p__Cyanobacteria;c__Synechococcophycideae;o__Pseudanabaenales;f__Pseudanabaenaceae;g__Pseudanabaena	0.0%	0.0%
	k__Bacteria;p__Cyanobacteria;c__Synechococcophycideae;o__Synechococcales;f__Acaryochloridaceae;g__Acaryochloris	0.0%	0.0%
	k__Bacteria;p__Cyanobacteria;c__Synechococcophycideae;o__Synechococcales;f__Synechococcaceae;g__	0.0%	0.0%
	k__Bacteria;p__Cyanobacteria;c__Synechococcophycideae;o__Synechococcales;f__Synechococcaceae;g__Paulinella	0.0%	0.0%
	k__Bacteria;p__Elusimicrobia;c__Elusimicrobia;o__Elusimicrobiales;f__;g__	0.0%	0.0%
	k__Bacteria;p__Elusimicrobia;c__Endomicrobia;o__;f__;g__	0.0%	0.0%
	k__Bacteria;p__Fibrobacteres;c__;o__;f__;g__	0.0%	0.0%
	k__Bacteria;p__Fibrobacteres;c__Fibrobacteria;o__258ds10;f__;g__	0.0%	0.0%
	k__Bacteria;p__Fibrobacteres;c__TG3;o__TG3-1;f__TSCOR003-O20;g__	0.0%	0.0%
	k__Bacteria;p__Firmicutes;c__Bacilli;o__Bacillales;f__;g__	0.0%	0.0%
	k__Bacteria;p__Firmicutes;c__Bacilli;o__Bacillales;f__Alicyclobacillaceae;g__Alicyclobacillus	0.0%	0.0%
	k__Bacteria;p__Firmicutes;c__Bacilli;o__Bacillales;f__Bacillaceae;g__	0.0%	0.0%
	k__Bacteria;p__Firmicutes;c__Bacilli;o__Bacillales;f__Bacillaceae;g__Bacillus	0.0%	0.0%
	k__Bacteria;p__Firmicutes;c__Bacilli;o__Bacillales;f__Bacillaceae;g__Geobacillus	0.0%	0.0%
	k__Bacteria;p__Firmicutes;c__Bacilli;o__Bacillales;f__Bacillaceae;g__Lentibacillus	0.0%	0.0%
	k__Bacteria;p__Firmicutes;c__Bacilli;o__Bacillales;f__Bacillaceae;g__Marinibacillus	0.0%	0.0%
	k__Bacteria;p__Firmicutes;c__Bacilli;o__Bacillales;f__Bacillaceae;g__Natronobacillus	0.0%	0.0%
	k__Bacteria;p__Firmicutes;c__Bacilli;o__Bacillales;f__Bacillaceae;g__Virgibacillus	0.0%	0.0%
	k__Bacteria;p__Firmicutes;c__Bacilli;o__Bacillales;f__Paenibacillaceae;g__	0.0%	0.0%
	k__Bacteria;p__Firmicutes;c__Bacilli;o__Bacillales;f__Paenibacillaceae;g__Ammoniphilus	0.0%	0.0%
	k__Bacteria;p__Firmicutes;c__Bacilli;o__Bacillales;f__Paenibacillaceae;g__Brevibacillus	0.0%	0.0%
	k__Bacteria;p__Firmicutes;c__Bacilli;o__Bacillales;f__Paenibacillaceae;g__Cohnella	0.0%	0.0%
	k__Bacteria;p__Firmicutes;c__Bacilli;o__Bacillales;f__Paenibacillaceae;g__Paenibacillus	0.0%	0.0%
	k__Bacteria;p__Firmicutes;c__Bacilli;o__Bacillales;f__Planococcaceae;g__	0.0%	0.0%
	k__Bacteria;p__Firmicutes;c__Bacilli;o__Bacillales;f__Planococcaceae;g__Lysinibacillus	0.0%	0.0%
	k__Bacteria;p__Firmicutes;c__Bacilli;o__Bacillales;f__Planococcaceae;g__Planococcus	0.0%	0.0%
	k__Bacteria;p__Firmicutes;c__Bacilli;o__Bacillales;f__Planococcaceae;g__Planomicrobium	0.0%	0.0%
	k__Bacteria;p__Firmicutes;c__Bacilli;o__Bacillales;f__Planococcaceae;g__Rummeliibacillus	0.0%	0.0%
	k__Bacteria;p__Firmicutes;c__Bacilli;o__Bacillales;f__Planococcaceae;g__Solibacillus	0.0%	0.0%
	k__Bacteria;p__Firmicutes;c__Bacilli;o__Bacillales;f__Planococcaceae;g__Staphylococcus	0.0%	0.0%
	k__Bacteria;p__Firmicutes;c__Bacilli;o__Bacillales;f__Staphylococcaceae;g__	0.0%	0.0%
	k__Bacteria;p__Firmicutes;c__Bacilli;o__Bacillales;f__Staphylococcaceae;g__Jeotgalicoccus	0.0%	0.0%
	k__Bacteria;p__Firmicutes;c__Bacilli;o__Bacillales;f__Staphylococcaceae;g__Macrococcus	0.0%	0.0%
	k__Bacteria;p__Firmicutes;c__Bacilli;o__Bacillales;f__Staphylococcaceae;g__Salinicoccus	0.0%	0.0%
	k__Bacteria;p__Firmicutes;c__Bacilli;o__Bacillales;f__Staphylococcaceae;g__Staphylococcus	0.1%	0.1%
	k__Bacteria;p__Firmicutes;c__Bacilli;o__Bacillales;f__Thermoactinomycetaceae;g__Planifilum	0.0%	0.0%
	k__Bacteria;p__Firmicutes;c__Bacilli;o__Bacillales;f__[Exiguobacteraceae];g__	0.0%	0.0%
	k__Bacteria;p__Firmicutes;c__Bacilli;o__Bacillales;f__[Exiguobacteraceae];g__Exiguobacterium	0.0%	0.0%
	k__Bacteria;p__Firmicutes;c__Bacilli;o__Gemellales;f__Gemellaceae;g__	0.0%	0.0%
	k__Bacteria;p__Firmicutes;c__Bacilli;o__Lactobacillales;f__;g__	0.0%	0.0%
	k__Bacteria;p__Firmicutes;c__Bacilli;o__Lactobacillales;f__Aerococcaceae;g__	0.0%	0.0%
	k__Bacteria;p__Firmicutes;c__Bacilli;o__Lactobacillales;f__Aerococcaceae;g__Aerococcus	0.0%	0.0%
	k__Bacteria;p__Firmicutes;c__Bacilli;o__Lactobacillales;f__Aerococcaceae;g__Alloiococcus	0.0%	0.0%
	k__Bacteria;p__Firmicutes;c__Bacilli;o__Lactobacillales;f__Aerococcaceae;g__Facklamia	0.0%	0.0%
	k__Bacteria;p__Firmicutes;c__Bacilli;o__Lactobacillales;f__Carnobacteriaceae;g__	0.0%	0.0%
	k__Bacteria;p__Firmicutes;c__Bacilli;o__Lactobacillales;f__Carnobacteriaceae;g__Granulicatella	0.0%	0.0%
	k__Bacteria;p__Firmicutes;c__Bacilli;o__Lactobacillales;f__Enterococcaceae;g__Enterococcus	0.0%	0.0%
	k__Bacteria;p__Firmicutes;c__Bacilli;o__Lactobacillales;f__Enterococcaceae;g__Vagococcus	0.0%	0.0%
	k__Bacteria;p__Firmicutes;c__Bacilli;o__Lactobacillales;f__Lactobacillaceae;g__Lactobacillus	0.0%	0.0%
	k__Bacteria;p__Firmicutes;c__Bacilli;o__Lactobacillales;f__Lactobacillaceae;g__Pediococcus	0.0%	0.0%
	k__Bacteria;p__Firmicutes;c__Bacilli;o__Lactobacillales;f__Leuconostocaceae;g__	0.0%	0.0%
	k__Bacteria;p__Firmicutes;c__Bacilli;o__Lactobacillales;f__Leuconostocaceae;g__Leuconostoc	0.0%	0.0%
	k__Bacteria;p__Firmicutes;c__Bacilli;o__Lactobacillales;f__Leuconostocaceae;g__Weissella	0.0%	0.0%
	k__Bacteria;p__Firmicutes;c__Bacilli;o__Lactobacillales;f__Streptococcaceae;g__Lactococcus	0.0%	0.0%
	k__Bacteria;p__Firmicutes;c__Bacilli;o__Lactobacillales;f__Streptococcaceae;g__Streptococcus	0.0%	0.0%
	k__Bacteria;p__Firmicutes;c__Clostridia;o__Clostridiales;f__;g__	0.0%	0.0%
	k__Bacteria;p__Firmicutes;c__Clostridia;o__Clostridiales;f__Christensenellaceae;g__	0.0%	0.0%
	k__Bacteria;p__Firmicutes;c__Clostridia;o__Clostridiales;f__Clostridiaceae;g__	0.0%	0.0%

	k__Bacteria;p__Firmicutes;c__Clostridia;o__Clostridiales;f__Clostridiaceae;g__Caloramator	0.0%	0.0%
	k__Bacteria;p__Firmicutes;c__Clostridia;o__Clostridiales;f__Clostridiaceae;g__Clostridium	0.0%	0.0%
	k__Bacteria;p__Firmicutes;c__Clostridia;o__Clostridiales;f__Clostridiaceae;g__Thermoanaerobacterium	0.0%	0.0%
	k__Bacteria;p__Firmicutes;c__Clostridia;o__Clostridiales;f__Clostridiaceae;g__Tindallia_Anoxynatronum	0.0%	0.0%
	k__Bacteria;p__Firmicutes;c__Clostridia;o__Clostridiales;f__Dehalobacteriaceae;g__	0.0%	0.0%
	k__Bacteria;p__Firmicutes;c__Clostridia;o__Clostridiales;f__EtOH8;g__	0.0%	0.0%
	k__Bacteria;p__Firmicutes;c__Clostridia;o__Clostridiales;f__Eubacteriaceae;g__Acetobacterium	0.0%	0.0%
	k__Bacteria;p__Firmicutes;c__Clostridia;o__Clostridiales;f__Heliobacteriaceae;g__	0.0%	0.0%
	k__Bacteria;p__Firmicutes;c__Clostridia;o__Clostridiales;f__JTB215;g__	0.0%	0.0%
	k__Bacteria;p__Firmicutes;c__Clostridia;o__Clostridiales;f__Lachnospiraceae;g__	0.0%	0.0%
	k__Bacteria;p__Firmicutes;c__Clostridia;o__Clostridiales;f__Lachnospiraceae;g__Butyrivibrio	0.0%	0.0%
	k__Bacteria;p__Firmicutes;c__Clostridia;o__Clostridiales;f__Lachnospiraceae;g__Coprococcus	0.0%	0.0%
	k__Bacteria;p__Firmicutes;c__Clostridia;o__Clostridiales;f__Peptococcaceae;g__	0.0%	0.0%
	k__Bacteria;p__Firmicutes;c__Clostridia;o__Clostridiales;f__Peptococcaceae;g__Desulfotomaculum	0.0%	0.0%
	k__Bacteria;p__Firmicutes;c__Clostridia;o__Clostridiales;f__Peptostreptococcaceae;g__	0.0%	0.0%
	k__Bacteria;p__Firmicutes;c__Clostridia;o__Clostridiales;f__Peptostreptococcaceae;g__Clostridium	0.0%	0.0%
	k__Bacteria;p__Firmicutes;c__Clostridia;o__Clostridiales;f__Peptostreptococcaceae;g__Tepidibacter	0.0%	0.0%
	k__Bacteria;p__Firmicutes;c__Clostridia;o__Clostridiales;f__Ruminococcaceae;g__	0.0%	0.0%
	k__Bacteria;p__Firmicutes;c__Clostridia;o__Clostridiales;f__Ruminococcaceae;g__Ethanoligenens	0.0%	0.0%
	k__Bacteria;p__Firmicutes;c__Clostridia;o__Clostridiales;f__Ruminococcaceae;g__Oscillospira	0.0%	0.0%
	k__Bacteria;p__Firmicutes;c__Clostridia;o__Clostridiales;f__SBYG_4172;g__	0.0%	0.0%
	k__Bacteria;p__Firmicutes;c__Clostridia;o__Clostridiales;f__Symbiobacteriaceae;g__	0.0%	0.0%
	k__Bacteria;p__Firmicutes;c__Clostridia;o__Clostridiales;f__Veillonellaceae;g__	0.0%	0.0%
	k__Bacteria;p__Firmicutes;c__Clostridia;o__Clostridiales;f__Veillonellaceae;g__Dialister	0.0%	0.0%
	k__Bacteria;p__Firmicutes;c__Clostridia;o__Clostridiales;f__Veillonellaceae;g__Megasphaera	0.0%	0.0%
	k__Bacteria;p__Firmicutes;c__Clostridia;o__Clostridiales;f__Veillonellaceae;g__Phascolarctobacterium	0.0%	0.0%
	k__Bacteria;p__Firmicutes;c__Clostridia;o__Clostridiales;f__Veillonellaceae;g__Selenomonas	0.0%	0.0%
	k__Bacteria;p__Firmicutes;c__Clostridia;o__Clostridiales;f__Veillonellaceae;g__Sporomusa	0.0%	0.0%
	k__Bacteria;p__Firmicutes;c__Clostridia;o__Clostridiales;f__Veillonellaceae;g__Thermosinus	0.0%	0.0%
	k__Bacteria;p__Firmicutes;c__Clostridia;o__Clostridiales;f__Veillonellaceae;g__Veillonella	0.0%	0.0%
	k__Bacteria;p__Firmicutes;c__Clostridia;o__Clostridiales;f__[Acidaminobacteraceae];g__Acidaminobacter	0.0%	0.0%
	k__Bacteria;p__Firmicutes;c__Clostridia;o__Clostridiales;f__[Acidaminobacteraceae];g__Fusibacter	0.0%	0.0%
	k__Bacteria;p__Firmicutes;c__Clostridia;o__Clostridiales;f__[Mogibacteriaceae];g__	0.0%	0.0%
	k__Bacteria;p__Firmicutes;c__Clostridia;o__Clostridiales;f__[Tissierellaceae];g__Anaerococcus	0.0%	0.0%
	k__Bacteria;p__Firmicutes;c__Clostridia;o__Clostridiales;f__[Tissierellaceae];g__Finegoldia	0.0%	0.0%
	k__Bacteria;p__Firmicutes;c__Clostridia;o__Clostridiales;f__[Tissierellaceae];g__Gallicola	0.0%	0.0%
	k__Bacteria;p__Firmicutes;c__Clostridia;o__Clostridiales;f__[Tissierellaceae];g__Peptoniphilus	0.0%	0.0%
	k__Bacteria;p__Firmicutes;c__Clostridia;o__Clostridiales;f__[Tissierellaceae];g__Tissierella_Soehngenia	0.0%	0.0%
	k__Bacteria;p__Firmicutes;c__Clostridia;o__Halanaerobiales;f__Halobacteroidaceae;g__Halanaerobacter	0.0%	0.0%
	k__Bacteria;p__Firmicutes;c__Clostridia;o__Natranaerobiales;f__;g__	0.0%	0.0%
	k__Bacteria;p__Firmicutes;c__Clostridia;o__SHA-98;f__;g__	0.0%	0.0%
	k__Bacteria;p__Firmicutes;c__Clostridia;o__Thermoanaerobacterales;f__Thermoanaerobacterales Family III. Incertae Sedis;g__Thermovenabulum	0.0%	0.0%
	k__Bacteria;p__Firmicutes;c__Erysipelotrichi;o__Erysipelotrichales;f__Erysipelotrichaceae;g__	0.0%	0.0%
	k__Bacteria;p__Firmicutes;c__Erysipelotrichi;o__Erysipelotrichales;f__Erysipelotrichaceae;g__Catenibacterium	0.0%	0.0%
	k__Bacteria;p__Firmicutes;c__Erysipelotrichi;o__Erysipelotrichales;f__Erysipelotrichaceae;g__PSB-M-3	0.0%	0.0%
	k__Bacteria;p__Fusobacteria;c__Fusobacteriia;o__Fusobacteriales;f__;g__	0.0%	0.0%
	k__Bacteria;p__Fusobacteria;c__Fusobacteriia;o__Fusobacteriales;f__Fusobacteriaceae;g__Fusobacterium	0.0%	0.0%
	k__Bacteria;p__Fusobacteria;c__Fusobacteriia;o__Fusobacteriales;f__Leptotrichiaceae;g__Leptotrichia	0.0%	0.0%
	k__Bacteria;p__GN02;c__BD1-5;o__;f__;g__	0.0%	0.0%
	k__Bacteria;p__Gemmatimonadetes;c__Gemm-1;o__;f__;g__	0.0%	0.0%
	k__Bacteria;p__Gemmatimonadetes;c__Gemm-3;o__;f__;g__	0.0%	0.0%
	k__Bacteria;p__Gemmatimonadetes;c__Gemmatimonadetes;o__;f__;g__	0.0%	0.0%
	k__Bacteria;p__Gemmatimonadetes;c__Gemmatimonadetes;o__Gemmatimonadales;f__Gemmatimonadaceae;g__Gemmatimonas	0.0%	0.0%
	k__Bacteria;p__Gemmatimonadetes;c__Gemmatimonadetes;o__KD8-87;f__;g__	0.0%	0.0%
	k__Bacteria;p__NKB19;c__;o__;f__;g__	0.0%	0.0%
	k__Bacteria;p__NKB19;c__TSBW08;o__;f__;g__	0.0%	0.0%
	k__Bacteria;p__Nitrospirae;c__Nitrospira;o__Nitrospirales;f__0319-6A21;g__	0.0%	0.0%

k__Bacteria;p__Nitrospirae;c__Nitrospira;o__Nitrospirales;f__FW;g__4-29	0.0%	0.0%
k__Bacteria;p__Nitrospirae;c__Nitrospira;o__Nitrospirales;f__Nitrospiraceae;g__Nitrospira	0.0%	0.0%
k__Bacteria;p__Nitrospirae;c__Nitrospira;o__Nitrospirales;f__[Thermodesulfovibrionaceae];g__GOUTA19	0.0%	0.0%
k__Bacteria;p__OD1;c__SM2F11;o__;f__;g__	0.0%	0.0%
k__Bacteria;p__OD1;c__ZB2;o__;f__;g__	0.0%	0.0%
k__Bacteria;p__OP1;c__MSBL6;o__;f__;g__	0.0%	0.0%
k__Bacteria;p__Planctomycetes;c__BD7-11;o__;f__;g__	0.0%	0.0%
k__Bacteria;p__Planctomycetes;c__ODP123;o__;f__;g__	0.0%	0.0%
k__Bacteria;p__Planctomycetes;c__OM190;o__CL500-15;f__;g__	0.0%	0.0%
k__Bacteria;p__Planctomycetes;c__Phycisphaerae;o__Phycisphaerales;f__;g__	0.0%	0.0%
k__Bacteria;p__Planctomycetes;c__Phycisphaerae;o__Pla1;f__;g__	0.0%	0.0%
k__Bacteria;p__Planctomycetes;c__Phycisphaerae;o__WD2101;f__;g__	0.0%	0.0%
k__Bacteria;p__Planctomycetes;c__Planctomycetia;o__Gemmatales;f__Gemmataceae;g__Gemmata	0.0%	0.0%
k__Bacteria;p__Planctomycetes;c__Planctomycetia;o__Gemmatales;f__Isosphaeraceae;g__	0.0%	0.0%
k__Bacteria;p__Planctomycetes;c__Planctomycetia;o__Pirellulales;f__Pirellulaceae;g__	0.1%	0.0%
k__Bacteria;p__Planctomycetes;c__Planctomycetia;o__Pirellulales;f__Pirellulaceae;g__Pirellula	0.0%	0.0%
k__Bacteria;p__Planctomycetes;c__Planctomycetia;o__Pirellulales;f__Pirellulaceae;g__planctomycete	0.0%	0.0%
k__Bacteria;p__Planctomycetes;c__Planctomycetia;o__Planctomycetales;f__Planctomycetaceae;g__Planctomyces	0.0%	0.0%
k__Bacteria;p__Proteobacteria;c__;o__;f__;g__	0.0%	0.0%
k__Bacteria;p__Proteobacteria;c__Alphaproteobacteria;o__;f__;g__	0.0%	0.0%
k__Bacteria;p__Proteobacteria;c__Alphaproteobacteria;o__BD7-3;f__;g__	0.0%	0.0%
k__Bacteria;p__Proteobacteria;c__Alphaproteobacteria;o__Caulobacterales;f__;g__	0.0%	0.0%
k__Bacteria;p__Proteobacteria;c__Alphaproteobacteria;o__Caulobacterales;f__Caulobacteraceae;g__	1.5%	2.1%
k__Bacteria;p__Proteobacteria;c__Alphaproteobacteria;o__Caulobacterales;f__Caulobacteraceae;g__Arthrospira	0.0%	0.0%
k__Bacteria;p__Proteobacteria;c__Alphaproteobacteria;o__Caulobacterales;f__Caulobacteraceae;g__Asticcacaulis	0.1%	0.1%
k__Bacteria;p__Proteobacteria;c__Alphaproteobacteria;o__Caulobacterales;f__Caulobacteraceae;g__Brevundimonas	0.3%	0.4%
k__Bacteria;p__Proteobacteria;c__Alphaproteobacteria;o__Caulobacterales;f__Caulobacteraceae;g__Caulobacter	0.0%	0.0%
k__Bacteria;p__Proteobacteria;c__Alphaproteobacteria;o__Caulobacterales;f__Caulobacteraceae;g__Mycoplana	0.0%	0.0%
k__Bacteria;p__Proteobacteria;c__Alphaproteobacteria;o__Caulobacterales;f__Caulobacteraceae;g__Phenylobacterium	0.1%	0.2%
k__Bacteria;p__Proteobacteria;c__Alphaproteobacteria;o__Ellin329;f__;g__	0.0%	0.0%
k__Bacteria;p__Proteobacteria;c__Alphaproteobacteria;o__Kiloniellales;f__;g__	0.0%	0.0%
k__Bacteria;p__Proteobacteria;c__Alphaproteobacteria;o__Kiloniellales;f__Kiloniellaceae;g__Thalassospira	0.0%	0.0%
k__Bacteria;p__Proteobacteria;c__Alphaproteobacteria;o__Rhizobiales;f__;g__	0.2%	0.3%
k__Bacteria;p__Proteobacteria;c__Alphaproteobacteria;o__Rhizobiales;f__Aurantimonadaceae;g__	0.0%	0.0%
k__Bacteria;p__Proteobacteria;c__Alphaproteobacteria;o__Rhizobiales;f__Aurantimonadaceae;g__Aurantimonas	0.0%	0.0%
k__Bacteria;p__Proteobacteria;c__Alphaproteobacteria;o__Rhizobiales;f__Bartonellaceae;g__	0.0%	0.0%
k__Bacteria;p__Proteobacteria;c__Alphaproteobacteria;o__Rhizobiales;f__Beijerinckiaceae;g__	0.0%	0.0%
k__Bacteria;p__Proteobacteria;c__Alphaproteobacteria;o__Rhizobiales;f__Beijerinckiaceae;g__Chelatococcus	0.0%	0.0%
k__Bacteria;p__Proteobacteria;c__Alphaproteobacteria;o__Rhizobiales;f__Bradyrhizobiaceae;g__	0.0%	0.0%
k__Bacteria;p__Proteobacteria;c__Alphaproteobacteria;o__Rhizobiales;f__Bradyrhizobiaceae;g__Balneimonas	0.0%	0.0%
k__Bacteria;p__Proteobacteria;c__Alphaproteobacteria;o__Rhizobiales;f__Bradyrhizobiaceae;g__Bosea	0.0%	0.0%
k__Bacteria;p__Proteobacteria;c__Alphaproteobacteria;o__Rhizobiales;f__Bradyrhizobiaceae;g__Bradyrhizobium	0.0%	0.0%
k__Bacteria;p__Proteobacteria;c__Alphaproteobacteria;o__Rhizobiales;f__Brucellaceae;g__	0.0%	0.0%
k__Bacteria;p__Proteobacteria;c__Alphaproteobacteria;o__Rhizobiales;f__Brucellaceae;g__Ochrobactrum	0.0%	0.0%
k__Bacteria;p__Proteobacteria;c__Alphaproteobacteria;o__Rhizobiales;f__Brucellaceae;g__Pseudochrobactrum	0.0%	0.0%
k__Bacteria;p__Proteobacteria;c__Alphaproteobacteria;o__Rhizobiales;f__Cohaesibacteraceae;g__	0.0%	0.0%
k__Bacteria;p__Proteobacteria;c__Alphaproteobacteria;o__Rhizobiales;f__Hyphomicrobiaceae;g__	0.0%	0.0%
k__Bacteria;p__Proteobacteria;c__Alphaproteobacteria;o__Rhizobiales;f__Hyphomicrobiaceae;g__Devosia	0.2%	0.2%
k__Bacteria;p__Proteobacteria;c__Alphaproteobacteria;o__Rhizobiales;f__Hyphomicrobiaceae;g__Hyphomicrobium	0.0%	0.0%
k__Bacteria;p__Proteobacteria;c__Alphaproteobacteria;o__Rhizobiales;f__Hyphomicrobiaceae;g__Pannonibacter	0.0%	0.0%
k__Bacteria;p__Proteobacteria;c__Alphaproteobacteria;o__Rhizobiales;f__Hyphomicrobiaceae;g__Parvibaculum	0.0%	0.0%
k__Bacteria;p__Proteobacteria;c__Alphaproteobacteria;o__Rhizobiales;f__Hyphomicrobiaceae;g__Pedomicrobium	0.0%	0.0%
k__Bacteria;p__Proteobacteria;c__Alphaproteobacteria;o__Rhizobiales;f__Hyphomicrobiaceae;g__Polymorphum	0.0%	0.0%
k__Bacteria;p__Proteobacteria;c__Alphaproteobacteria;o__Rhizobiales;f__Hyphomicrobiaceae;g__Rhodoplanes	0.0%	0.0%
k__Bacteria;p__Proteobacteria;c__Alphaproteobacteria;o__Rhizobiales;f__Methylobacteriaceae;g__	0.0%	0.1%
k__Bacteria;p__Proteobacteria;c__Alphaproteobacteria;o__Rhizobiales;f__Methylobacteriaceae;g__Methylobacterium	0.0%	0.0%
k__Bacteria;p__Proteobacteria;c__Alphaproteobacteria;o__Rhizobiales;f__Methylocystaceae;g__	0.0%	0.0%
k__Bacteria;p__Proteobacteria;c__Alphaproteobacteria;o__Rhizobiales;f__Methylocystaceae;g__Methylopila	1.8%	4.2%
k__Bacteria;p__Proteobacteria;c__Alphaproteobacteria;o__Rhizobiales;f__Methylocystaceae;g__Methylosinus	0.0%	0.0%

	k__Bacteria;p__Proteobacteria;c__Alphaproteobacteria;o__Rhizobiales;f__Methylocystaceae;g__Pleomorphomonas	0.0%	0.0%
	k__Bacteria;p__Proteobacteria;c__Alphaproteobacteria;o__Rhizobiales;f__Phyllobacteriaceae;g__	0.0%	0.1%
	k__Bacteria;p__Proteobacteria;c__Alphaproteobacteria;o__Rhizobiales;f__Phyllobacteriaceae;g__Chelativorans	0.0%	0.0%
	k__Bacteria;p__Proteobacteria;c__Alphaproteobacteria;o__Rhizobiales;f__Phyllobacteriaceae;g__Hoeflea	0.0%	0.0%
	k__Bacteria;p__Proteobacteria;c__Alphaproteobacteria;o__Rhizobiales;f__Phyllobacteriaceae;g__Mesorhizobium	0.0%	0.0%
	k__Bacteria;p__Proteobacteria;c__Alphaproteobacteria;o__Rhizobiales;f__Phyllobacteriaceae;g__Nitratireductor	0.0%	0.0%
	k__Bacteria;p__Proteobacteria;c__Alphaproteobacteria;o__Rhizobiales;f__Phyllobacteriaceae;g__Phyllobacterium	0.0%	0.0%
	k__Bacteria;p__Proteobacteria;c__Alphaproteobacteria;o__Rhizobiales;f__Rhizobiaceae;g__	0.0%	0.0%
	k__Bacteria;p__Proteobacteria;c__Alphaproteobacteria;o__Rhizobiales;f__Rhizobiaceae;g__Agrobacterium	0.3%	0.4%
	k__Bacteria;p__Proteobacteria;c__Alphaproteobacteria;o__Rhizobiales;f__Rhizobiaceae;g__Amorphomonas	0.0%	0.0%
	k__Bacteria;p__Proteobacteria;c__Alphaproteobacteria;o__Rhizobiales;f__Rhizobiaceae;g__Ensifer	0.0%	0.0%
	k__Bacteria;p__Proteobacteria;c__Alphaproteobacteria;o__Rhizobiales;f__Rhizobiaceae;g__Kaistia	0.0%	0.0%
	k__Bacteria;p__Proteobacteria;c__Alphaproteobacteria;o__Rhizobiales;f__Rhizobiaceae;g__Prosthecomicrobium	0.0%	0.0%
	k__Bacteria;p__Proteobacteria;c__Alphaproteobacteria;o__Rhizobiales;f__Rhizobiaceae;g__Shinella	0.0%	0.0%
	k__Bacteria;p__Proteobacteria;c__Alphaproteobacteria;o__Rhizobiales;f__Rhizobiaceae;g__Sinorhizobium	0.0%	0.0%
	k__Bacteria;p__Proteobacteria;c__Alphaproteobacteria;o__Rhizobiales;f__Rhodobiaceae;g__	0.0%	0.0%
	k__Bacteria;p__Proteobacteria;c__Alphaproteobacteria;o__Rhizobiales;f__Rhodobiaceae;g__Afifella	0.0%	0.0%
	k__Bacteria;p__Proteobacteria;c__Alphaproteobacteria;o__Rhizobiales;f__Xanthobacteraceae;g__	0.0%	0.0%
	k__Bacteria;p__Proteobacteria;c__Alphaproteobacteria;o__Rhizobiales;f__Xanthobacteraceae;g__Ancylobacter	0.0%	0.0%
	k__Bacteria;p__Proteobacteria;c__Alphaproteobacteria;o__Rhizobiales;f__Xanthobacteraceae;g__Azorhizobium	0.0%	0.0%
	k__Bacteria;p__Proteobacteria;c__Alphaproteobacteria;o__Rhizobiales;f__Xanthobacteraceae;g__Labrys	0.0%	0.0%
	k__Bacteria;p__Proteobacteria;c__Alphaproteobacteria;o__Rhizobiales;f__Xanthobacteraceae;g__Xanthobacter	0.0%	0.0%
	k__Bacteria;p__Proteobacteria;c__Alphaproteobacteria;o__Rhodobacterales;f__Hyphomonadaceae;g__	0.0%	0.0%
	k__Bacteria;p__Proteobacteria;c__Alphaproteobacteria;o__Rhodobacterales;f__Hyphomonadaceae;g__Hyphomonas	0.0%	0.0%
	k__Bacteria;p__Proteobacteria;c__Alphaproteobacteria;o__Rhodobacterales;f__Rhodobacteraceae;g__	0.1%	0.1%
	k__Bacteria;p__Proteobacteria;c__Alphaproteobacteria;o__Rhodobacterales;f__Rhodobacteraceae;g__Albidovulum	0.0%	0.0%
	k__Bacteria;p__Proteobacteria;c__Alphaproteobacteria;o__Rhodobacterales;f__Rhodobacteraceae;g__Amaricoccus	0.0%	0.0%
	k__Bacteria;p__Proteobacteria;c__Alphaproteobacteria;o__Rhodobacterales;f__Rhodobacteraceae;g__Anaerospora	0.0%	0.0%
	k__Bacteria;p__Proteobacteria;c__Alphaproteobacteria;o__Rhodobacterales;f__Rhodobacteraceae;g__Paracoccus	0.1%	0.2%
	k__Bacteria;p__Proteobacteria;c__Alphaproteobacteria;o__Rhodobacterales;f__Rhodobacteraceae;g__Rhodobacter	0.1%	0.1%
	k__Bacteria;p__Proteobacteria;c__Alphaproteobacteria;o__Rhodobacterales;f__Rhodobacteraceae;g__Rubellimicrobium	0.0%	0.0%
	k__Bacteria;p__Proteobacteria;c__Alphaproteobacteria;o__Rhodobacterales;f__Rhodobacteraceae;g__Shimia	0.0%	0.0%
	k__Bacteria;p__Proteobacteria;c__Alphaproteobacteria;o__Rhodospirillales;f__;g__	0.0%	0.0%
	k__Bacteria;p__Proteobacteria;c__Alphaproteobacteria;o__Rhodospirillales;f__Acetobacteraceae;g__	0.0%	0.0%
	k__Bacteria;p__Proteobacteria;c__Alphaproteobacteria;o__Rhodospirillales;f__Acetobacteraceae;g__Acetobacter	0.0%	0.0%
	k__Bacteria;p__Proteobacteria;c__Alphaproteobacteria;o__Rhodospirillales;f__Acetobacteraceae;g__Neoasaia	0.0%	0.0%
	k__Bacteria;p__Proteobacteria;c__Alphaproteobacteria;o__Rhodospirillales;f__Acetobacteraceae;g__Roseomonas	0.0%	0.0%
	k__Bacteria;p__Proteobacteria;c__Alphaproteobacteria;o__Rhodospirillales;f__Acetobacteraceae;g__Swaminathania	0.0%	0.0%
	k__Bacteria;p__Proteobacteria;c__Alphaproteobacteria;o__Rhodospirillales;f__Rhodospirillaceae;g__	0.0%	0.0%
	k__Bacteria;p__Proteobacteria;c__Alphaproteobacteria;o__Rhodospirillales;f__Rhodospirillaceae;g__Azospirillum	0.0%	0.1%
	k__Bacteria;p__Proteobacteria;c__Alphaproteobacteria;o__Rhodospirillales;f__Rhodospirillaceae;g__Magnetospirillum	0.0%	0.0%
	k__Bacteria;p__Proteobacteria;c__Alphaproteobacteria;o__Rhodospirillales;f__Rhodospirillaceae;g__Novispirillum	0.0%	0.0%
	k__Bacteria;p__Proteobacteria;c__Alphaproteobacteria;o__Rhodospirillales;f__Rhodospirillaceae;g__Phaeospirillum	0.0%	0.0%
	k__Bacteria;p__Proteobacteria;c__Alphaproteobacteria;o__Rhodospirillales;f__Rhodospirillaceae;g__Rhodospirillum	0.0%	0.0%
	k__Bacteria;p__Proteobacteria;c__Alphaproteobacteria;o__Rhodospirillales;f__Rhodospirillaceae;g__Skermanella	0.0%	0.0%
	k__Bacteria;p__Proteobacteria;c__Alphaproteobacteria;o__Rickettsiales;f__;g__	0.0%	0.0%
	k__Bacteria;p__Proteobacteria;c__Alphaproteobacteria;o__Rickettsiales;f__Rickettsiaceae;g__	0.0%	0.0%
	k__Bacteria;p__Proteobacteria;c__Alphaproteobacteria;o__Rickettsiales;f__Rickettsiaceae;g__Orientia	0.0%	0.0%
	k__Bacteria;p__Proteobacteria;c__Alphaproteobacteria;o__Rickettsiales;f__Rickettsiaceae;g__Wolbachia	7.1%	12.5%
	k__Bacteria;p__Proteobacteria;c__Alphaproteobacteria;o__Rickettsiales;f__mitochondria;g__	2.4%	1.6%
	k__Bacteria;p__Proteobacteria;c__Alphaproteobacteria;o__Rickettsiales;f__mitochondria;g__Acorus	0.0%	0.0%
	k__Bacteria;p__Proteobacteria;c__Alphaproteobacteria;o__Rickettsiales;f__mitochondria;g__Angiopteris	0.0%	0.0%
	k__Bacteria;p__Proteobacteria;c__Alphaproteobacteria;o__Rickettsiales;f__mitochondria;g__Arabidopsis	0.0%	0.0%
	k__Bacteria;p__Proteobacteria;c__Alphaproteobacteria;o__Rickettsiales;f__mitochondria;g__Aristolochia	0.0%	0.0%
	k__Bacteria;p__Proteobacteria;c__Alphaproteobacteria;o__Rickettsiales;f__mitochondria;g__Asarum	0.0%	0.0%
	k__Bacteria;p__Proteobacteria;c__Alphaproteobacteria;o__Rickettsiales;f__mitochondria;g__Botrychium	0.0%	0.0%
	k__Bacteria;p__Proteobacteria;c__Alphaproteobacteria;o__Rickettsiales;f__mitochondria;g__Calycanthus	0.0%	0.0%
	k__Bacteria;p__Proteobacteria;c__Alphaproteobacteria;o__Rickettsiales;f__mitochondria;g__Carica	0.0%	0.0%
	k__Bacteria;p__Proteobacteria;c__Alphaproteobacteria;o__Rickettsiales;f__mitochondria;g__Carludovica	0.0%	0.0%

k__Bacteria;p__Proteobacteria;c__Alphaproteobacteria;o__Rickettsiales;f__mitochondria;g__Cinnamomum	0.0%	0.0%
k__Bacteria;p__Proteobacteria;c__Alphaproteobacteria;o__Rickettsiales;f__mitochondria;g__Citrullus	0.0%	0.0%
k__Bacteria;p__Proteobacteria;c__Alphaproteobacteria;o__Rickettsiales;f__mitochondria;g__Didymeles	0.0%	0.0%
k__Bacteria;p__Proteobacteria;c__Alphaproteobacteria;o__Rickettsiales;f__mitochondria;g__Galbulimima	0.0%	0.0%
k__Bacteria;p__Proteobacteria;c__Alphaproteobacteria;o__Rickettsiales;f__mitochondria;g__Geranium	0.0%	0.0%
k__Bacteria;p__Proteobacteria;c__Alphaproteobacteria;o__Rickettsiales;f__mitochondria;g__Grevillea	1.5%	1.0%
k__Bacteria;p__Proteobacteria;c__Alphaproteobacteria;o__Rickettsiales;f__mitochondria;g__Gyrocarpus	0.0%	0.0%
k__Bacteria;p__Proteobacteria;c__Alphaproteobacteria;o__Rickettsiales;f__mitochondria;g__Helosis	0.0%	0.0%
k__Bacteria;p__Proteobacteria;c__Alphaproteobacteria;o__Rickettsiales;f__mitochondria;g__Lardizabala	0.0%	0.0%
k__Bacteria;p__Proteobacteria;c__Alphaproteobacteria;o__Rickettsiales;f__mitochondria;g__Lepidoceras	0.0%	0.0%
k__Bacteria;p__Proteobacteria;c__Alphaproteobacteria;o__Rickettsiales;f__mitochondria;g__Lupinus	0.0%	0.0%
k__Bacteria;p__Proteobacteria;c__Alphaproteobacteria;o__Rickettsiales;f__mitochondria;g__Ophioglossum	0.0%	0.0%
k__Bacteria;p__Proteobacteria;c__Alphaproteobacteria;o__Rickettsiales;f__mitochondria;g__Plantago	0.0%	0.0%
k__Bacteria;p__Proteobacteria;c__Alphaproteobacteria;o__Rickettsiales;f__mitochondria;g__Sarcandra	0.9%	0.5%
k__Bacteria;p__Proteobacteria;c__Alphaproteobacteria;o__Rickettsiales;f__mitochondria;g__Spirodela	0.0%	0.0%
k__Bacteria;p__Proteobacteria;c__Alphaproteobacteria;o__Rickettsiales;f__mitochondria;g__Treubia	0.0%	0.0%
k__Bacteria;p__Proteobacteria;c__Alphaproteobacteria;o__Rickettsiales;f__mitochondria;g__Trochodendron	0.0%	0.0%
k__Bacteria;p__Proteobacteria;c__Alphaproteobacteria;o__Rickettsiales;f__mitochondria;g__Victoria	0.0%	0.0%
k__Bacteria;p__Proteobacteria;c__Alphaproteobacteria;o__Rickettsiales;f__mitochondria;g__Zamia	0.0%	0.0%
k__Bacteria;p__Proteobacteria;c__Alphaproteobacteria;o__Rickettsiales;f__mitochondria;g__Zea	26.2%	14.7%
k__Bacteria;p__Proteobacteria;c__Alphaproteobacteria;o__Sphingomonadales;f__;g__	0.0%	0.0%
k__Bacteria;p__Proteobacteria;c__Alphaproteobacteria;o__Sphingomonadales;f__Erythrobacteraceae;g__	0.0%	0.0%
k__Bacteria;p__Proteobacteria;c__Alphaproteobacteria;o__Sphingomonadales;f__Erythrobacteraceae;g__Altererythrobacter	0.0%	0.0%
k__Bacteria;p__Proteobacteria;c__Alphaproteobacteria;o__Sphingomonadales;f__Erythrobacteraceae;g__Erythrobacter	0.0%	0.0%
k__Bacteria;p__Proteobacteria;c__Alphaproteobacteria;o__Sphingomonadales;f__Erythrobacteraceae;g__Erythromicrobium	0.0%	0.0%
k__Bacteria;p__Proteobacteria;c__Alphaproteobacteria;o__Sphingomonadales;f__Erythrobacteraceae;g__Lutibacterium	0.0%	0.0%
k__Bacteria;p__Proteobacteria;c__Alphaproteobacteria;o__Sphingomonadales;f__Sphingomonadaceae;g__	0.0%	0.0%
k__Bacteria;p__Proteobacteria;c__Alphaproteobacteria;o__Sphingomonadales;f__Sphingomonadaceae;g__Blastomonas	0.0%	0.0%
k__Bacteria;p__Proteobacteria;c__Alphaproteobacteria;o__Sphingomonadales;f__Sphingomonadaceae;g__Kaistobacter	0.0%	0.0%
k__Bacteria;p__Proteobacteria;c__Alphaproteobacteria;o__Sphingomonadales;f__Sphingomonadaceae;g__Novosphingobium	0.0%	0.1%
k__Bacteria;p__Proteobacteria;c__Alphaproteobacteria;o__Sphingomonadales;f__Sphingomonadaceae;g__Sphingobium	0.0%	0.0%
k__Bacteria;p__Proteobacteria;c__Alphaproteobacteria;o__Sphingomonadales;f__Sphingomonadaceae;g__Sphingomonas	0.1%	0.2%
k__Bacteria;p__Proteobacteria;c__Alphaproteobacteria;o__Sphingomonadales;f__Sphingomonadaceae;g__Sphingopyxis	0.0%	0.0%
k__Bacteria;p__Proteobacteria;c__Alphaproteobacteria;o__Sphingomonadales;f__Sphingomonadaceae;g__Sphingosinicella	0.0%	0.0%
k__Bacteria;p__Proteobacteria;c__Betaproteobacteria;o__;f__;g__	0.1%	0.1%
k__Bacteria;p__Proteobacteria;c__Betaproteobacteria;o__ASSO-13;f__;g__	0.0%	0.0%
k__Bacteria;p__Proteobacteria;c__Betaproteobacteria;o__Burkholderiales;f__;g__	0.0%	0.0%
k__Bacteria;p__Proteobacteria;c__Betaproteobacteria;o__Burkholderiales;f__Alcaligenaceae;g__	0.0%	0.0%
k__Bacteria;p__Proteobacteria;c__Betaproteobacteria;o__Burkholderiales;f__Alcaligenaceae;g__Achromobacter	0.0%	0.0%
k__Bacteria;p__Proteobacteria;c__Betaproteobacteria;o__Burkholderiales;f__Burkholderiaceae;g__	0.0%	0.0%
k__Bacteria;p__Proteobacteria;c__Betaproteobacteria;o__Burkholderiales;f__Burkholderiaceae;g__Burkholderia	0.0%	0.0%
k__Bacteria;p__Proteobacteria;c__Betaproteobacteria;o__Burkholderiales;f__Burkholderiaceae;g__Pandoraea	0.0%	0.0%
k__Bacteria;p__Proteobacteria;c__Betaproteobacteria;o__Burkholderiales;f__Comamonadaceae;g__	2.1%	3.3%
k__Bacteria;p__Proteobacteria;c__Betaproteobacteria;o__Burkholderiales;f__Comamonadaceae;g__Acidovorax	0.0%	0.0%
k__Bacteria;p__Proteobacteria;c__Betaproteobacteria;o__Burkholderiales;f__Comamonadaceae;g__Alicycliphilus	0.0%	0.0%
k__Bacteria;p__Proteobacteria;c__Betaproteobacteria;o__Burkholderiales;f__Comamonadaceae;g__Aquabacterium	0.0%	0.0%
k__Bacteria;p__Proteobacteria;c__Betaproteobacteria;o__Burkholderiales;f__Comamonadaceae;g__Aquincola	0.0%	0.0%
k__Bacteria;p__Proteobacteria;c__Betaproteobacteria;o__Burkholderiales;f__Comamonadaceae;g__Comamonas	0.0%	0.0%
k__Bacteria;p__Proteobacteria;c__Betaproteobacteria;o__Burkholderiales;f__Comamonadaceae;g__Curvibacter	0.0%	0.1%
k__Bacteria;p__Proteobacteria;c__Betaproteobacteria;o__Burkholderiales;f__Comamonadaceae;g__Delftia	0.1%	0.3%
k__Bacteria;p__Proteobacteria;c__Betaproteobacteria;o__Burkholderiales;f__Comamonadaceae;g__Diaphorobacter	0.0%	0.0%
k__Bacteria;p__Proteobacteria;c__Betaproteobacteria;o__Burkholderiales;f__Comamonadaceae;g__Hydrogenophaga	0.2%	0.3%
k__Bacteria;p__Proteobacteria;c__Betaproteobacteria;o__Burkholderiales;f__Comamonadaceae;g__Hylemonella	0.0%	0.0%
k__Bacteria;p__Proteobacteria;c__Betaproteobacteria;o__Burkholderiales;f__Comamonadaceae;g__Lampropedia	0.0%	0.0%
k__Bacteria;p__Proteobacteria;c__Betaproteobacteria;o__Burkholderiales;f__Comamonadaceae;g__Leptothrix	0.0%	0.0%
k__Bacteria;p__Proteobacteria;c__Betaproteobacteria;o__Burkholderiales;f__Comamonadaceae;g__Limnobacter	0.0%	0.0%
k__Bacteria;p__Proteobacteria;c__Betaproteobacteria;o__Burkholderiales;f__Comamonadaceae;g__Limnohabitans	0.0%	0.0%
k__Bacteria;p__Proteobacteria;c__Betaproteobacteria;o__Burkholderiales;f__Comamonadaceae;g__Methylibium	0.0%	0.0%
k__Bacteria;p__Proteobacteria;c__Betaproteobacteria;o__Burkholderiales;f__Comamonadaceae;g__Polaromonas	0.0%	0.0%

k__Bacteria;p__Proteobacteria;c__Betaproteobacteria;o__Burkholderiales;f__Comamonadaceae;g__Ramlibacter	0.2%	0.5%
k__Bacteria;p__Proteobacteria;c__Betaproteobacteria;o__Burkholderiales;f__Comamonadaceae;g__Rhodoferax	0.0%	0.0%
k__Bacteria;p__Proteobacteria;c__Betaproteobacteria;o__Burkholderiales;f__Comamonadaceae;g__Roseateles	0.0%	0.0%
k__Bacteria;p__Proteobacteria;c__Betaproteobacteria;o__Burkholderiales;f__Comamonadaceae;g__Rubrivivax	0.0%	0.0%
k__Bacteria;p__Proteobacteria;c__Betaproteobacteria;o__Burkholderiales;f__Comamonadaceae;g__Schlegelella	0.0%	0.0%
k__Bacteria;p__Proteobacteria;c__Betaproteobacteria;o__Burkholderiales;f__Comamonadaceae;g__Simplicispira	0.0%	0.0%
k__Bacteria;p__Proteobacteria;c__Betaproteobacteria;o__Burkholderiales;f__Comamonadaceae;g__Tepidimonas	0.0%	0.0%
k__Bacteria;p__Proteobacteria;c__Betaproteobacteria;o__Burkholderiales;f__Comamonadaceae;g__Thiomonas	0.0%	0.0%
k__Bacteria;p__Proteobacteria;c__Betaproteobacteria;o__Burkholderiales;f__Comamonadaceae;g__Variovorax	0.5%	0.6%
k__Bacteria;p__Proteobacteria;c__Betaproteobacteria;o__Burkholderiales;f__Comamonadaceae;g__Verminephrobacter	0.0%	0.0%
k__Bacteria;p__Proteobacteria;c__Betaproteobacteria;o__Burkholderiales;f__Comamonadaceae;g__Xenophilus	0.0%	0.0%
k__Bacteria;p__Proteobacteria;c__Betaproteobacteria;o__Burkholderiales;f__Oxalobacteraceae;g__	0.2%	0.4%
k__Bacteria;p__Proteobacteria;c__Betaproteobacteria;o__Burkholderiales;f__Oxalobacteraceae;g__Herbaspirillum	0.0%	0.0%
k__Bacteria;p__Proteobacteria;c__Betaproteobacteria;o__Burkholderiales;f__Oxalobacteraceae;g__Janthinobacterium	0.0%	0.0%
k__Bacteria;p__Proteobacteria;c__Betaproteobacteria;o__Burkholderiales;f__Oxalobacteraceae;g__Massilia	0.0%	0.0%
k__Bacteria;p__Proteobacteria;c__Betaproteobacteria;o__Burkholderiales;f__Oxalobacteraceae;g__Polynucleobacter	0.0%	0.0%
k__Bacteria;p__Proteobacteria;c__Betaproteobacteria;o__Burkholderiales;f__Oxalobacteraceae;g__Ralstonia	0.0%	0.0%
k__Bacteria;p__Proteobacteria;c__Betaproteobacteria;o__Ellin6067;f__;g__	0.0%	0.0%
k__Bacteria;p__Proteobacteria;c__Betaproteobacteria;o__Gallionellales;f__Gallionellaceae;g__	0.0%	0.0%
k__Bacteria;p__Proteobacteria;c__Betaproteobacteria;o__Gallionellales;f__Gallionellaceae;g__Gallionella	0.0%	0.0%
k__Bacteria;p__Proteobacteria;c__Betaproteobacteria;o__Hydrogenophilales;f__Hydrogenophilaceae;g__Thiobacillus	0.0%	0.0%
k__Bacteria;p__Proteobacteria;c__Betaproteobacteria;o__MND1;f__;g__	0.0%	0.0%
k__Bacteria;p__Proteobacteria;c__Betaproteobacteria;o__Methylophilales;f__;g__	0.0%	0.0%
k__Bacteria;p__Proteobacteria;c__Betaproteobacteria;o__Methylophilales;f__Methylophilaceae;g__	0.0%	0.0%
k__Bacteria;p__Proteobacteria;c__Betaproteobacteria;o__Methylophilales;f__Methylophilaceae;g__Methylotenera	0.0%	0.0%
k__Bacteria;p__Proteobacteria;c__Betaproteobacteria;o__Neisseriales;f__Neisseriaceae;g__	0.0%	0.0%
k__Bacteria;p__Proteobacteria;c__Betaproteobacteria;o__Neisseriales;f__Neisseriaceae;g__Alysiella	0.0%	0.0%
k__Bacteria;p__Proteobacteria;c__Betaproteobacteria;o__Neisseriales;f__Neisseriaceae;g__Chitinimonas	0.0%	0.0%
k__Bacteria;p__Proteobacteria;c__Betaproteobacteria;o__Neisseriales;f__Neisseriaceae;g__Kingella	0.0%	0.0%
k__Bacteria;p__Proteobacteria;c__Betaproteobacteria;o__Neisseriales;f__Neisseriaceae;g__Neisseria	0.0%	0.0%
k__Bacteria;p__Proteobacteria;c__Betaproteobacteria;o__Neisseriales;f__Neisseriaceae;g__Vogesella	0.0%	0.0%
k__Bacteria;p__Proteobacteria;c__Betaproteobacteria;o__Nitrosomonadales;f__Nitrosomonadaceae;g__	0.0%	0.0%
k__Bacteria;p__Proteobacteria;c__Betaproteobacteria;o__Rhodocyclales;f__Rhodocyclaceae;g__	0.1%	0.0%
k__Bacteria;p__Proteobacteria;c__Betaproteobacteria;o__Rhodocyclales;f__Rhodocyclaceae;g__Azoarcus	0.0%	0.0%
k__Bacteria;p__Proteobacteria;c__Betaproteobacteria;o__Rhodocyclales;f__Rhodocyclaceae;g__Azospira	0.0%	0.0%
k__Bacteria;p__Proteobacteria;c__Betaproteobacteria;o__Rhodocyclales;f__Rhodocyclaceae;g__Azovibrio	0.0%	0.0%
k__Bacteria;p__Proteobacteria;c__Betaproteobacteria;o__Rhodocyclales;f__Rhodocyclaceae;g__Dechloromonas	0.0%	0.0%
k__Bacteria;p__Proteobacteria;c__Betaproteobacteria;o__Rhodocyclales;f__Rhodocyclaceae;g__Dok59	0.0%	0.0%
k__Bacteria;p__Proteobacteria;c__Betaproteobacteria;o__Rhodocyclales;f__Rhodocyclaceae;g__Hydrogenophilus	0.0%	0.0%
k__Bacteria;p__Proteobacteria;c__Betaproteobacteria;o__Rhodocyclales;f__Rhodocyclaceae;g__Methyloversatilis	0.0%	0.0%
k__Bacteria;p__Proteobacteria;c__Betaproteobacteria;o__Rhodocyclales;f__Rhodocyclaceae;g__Propionivibrio	0.0%	0.0%
k__Bacteria;p__Proteobacteria;c__Betaproteobacteria;o__Rhodocyclales;f__Rhodocyclaceae;g__Rhodocyclus	0.0%	0.0%
k__Bacteria;p__Proteobacteria;c__Betaproteobacteria;o__Rhodocyclales;f__Rhodocyclaceae;g__Thauera	0.0%	0.0%
k__Bacteria;p__Proteobacteria;c__Betaproteobacteria;o__Rhodocyclales;f__Rhodocyclaceae;g__Uliginosibacterium	0.0%	0.0%
k__Bacteria;p__Proteobacteria;c__Betaproteobacteria;o__Rhodocyclales;f__Rhodocyclaceae;g__Zoogloea	0.0%	0.0%
k__Bacteria;p__Proteobacteria;c__Betaproteobacteria;o__SBla14;f__;g__	0.0%	0.0%
k__Bacteria;p__Proteobacteria;c__Betaproteobacteria;o__SC-I-84;f__;g__	0.0%	0.0%
k__Bacteria;p__Proteobacteria;c__Deltaproteobacteria;o__;f__;g__	0.0%	0.0%
k__Bacteria;p__Proteobacteria;c__Deltaproteobacteria;o__Bdellovibrionales;f__Bacteriovoracaceae;g__Bacteriovorax	0.0%	0.0%
k__Bacteria;p__Proteobacteria;c__Deltaproteobacteria;o__Bdellovibrionales;f__Bdellovibrionaceae;g__Bdellovibrio	0.0%	0.0%
k__Bacteria;p__Proteobacteria;c__Deltaproteobacteria;o__Desulfobacterales;f__Desulfobacteraceae;g__	0.0%	0.0%
k__Bacteria;p__Proteobacteria;c__Deltaproteobacteria;o__Desulfobacterales;f__Desulfobacteraceae;g__Desulfococcus	0.0%	0.0%
k__Bacteria;p__Proteobacteria;c__Deltaproteobacteria;o__Desulfobacterales;f__Desulfobulbaceae;g__	0.0%	0.0%
k__Bacteria;p__Proteobacteria;c__Deltaproteobacteria;o__Desulfobacterales;f__Desulfobulbaceae;g__Desulfobulbus	0.0%	0.0%
k__Bacteria;p__Proteobacteria;c__Deltaproteobacteria;o__Desulfovibrionales;f__Desulfomicrobiaceae;g__Desulfomicrobium	0.0%	0.0%
k__Bacteria;p__Proteobacteria;c__Deltaproteobacteria;o__Desulfovibrionales;f__Desulfovibrionaceae;g__Desulfovibrio	0.0%	0.0%
k__Bacteria;p__Proteobacteria;c__Deltaproteobacteria;o__Desulfuromonadales;f__Desulfuromonadaceae;g__	0.0%	0.0%
k__Bacteria;p__Proteobacteria;c__Deltaproteobacteria;o__Desulfuromonadales;f__Geobacteraceae;g__Geobacter	0.0%	0.0%
k__Bacteria;p__Proteobacteria;c__Deltaproteobacteria;o__Desulfuromonadales;f__Pelobacteraceae;g__	0.0%	0.0%

k__Bacteria;p__Proteobacteria;c__Deltaproteobacteria;o__GMD14H09;f__;g__	0.0%	0.0%
k__Bacteria;p__Proteobacteria;c__Deltaproteobacteria;o__MBNT15;f__;g__	0.0%	0.0%
k__Bacteria;p__Proteobacteria;c__Deltaproteobacteria;o__MIZ46;f__;g__	0.0%	0.0%
k__Bacteria;p__Proteobacteria;c__Deltaproteobacteria;o__Myxococcales;f__;g__	0.0%	0.0%
k__Bacteria;p__Proteobacteria;c__Deltaproteobacteria;o__Myxococcales;f__0319-6G20;g__	0.0%	0.0%
k__Bacteria;p__Proteobacteria;c__Deltaproteobacteria;o__Myxococcales;f__Haliangiaceae;g__	0.0%	0.0%
k__Bacteria;p__Proteobacteria;c__Deltaproteobacteria;o__Myxococcales;f__Haliangiaceae;g__Haliangium	0.0%	0.0%
k__Bacteria;p__Proteobacteria;c__Deltaproteobacteria;o__Myxococcales;f__Myxococcaceae;g__Anaeromyxobacter	0.0%	0.0%
k__Bacteria;p__Proteobacteria;c__Deltaproteobacteria;o__Myxococcales;f__Myxococcaceae;g__Myxococcus	0.0%	0.0%
k__Bacteria;p__Proteobacteria;c__Deltaproteobacteria;o__Myxococcales;f__Nannocystaceae;g__Nannocystis	0.0%	0.0%
k__Bacteria;p__Proteobacteria;c__Deltaproteobacteria;o__Myxococcales;f__Nannocystaceae;g__Plesiocystis	0.0%	0.0%
k__Bacteria;p__Proteobacteria;c__Deltaproteobacteria;o__Myxococcales;f__OM27;g__	0.0%	0.0%
k__Bacteria;p__Proteobacteria;c__Deltaproteobacteria;o__Myxococcales;f__Polyangiaceae;g__	0.0%	0.0%
k__Bacteria;p__Proteobacteria;c__Deltaproteobacteria;o__Myxococcales;f__Polyangiaceae;g__Aetherobacter	0.0%	0.0%
k__Bacteria;p__Proteobacteria;c__Deltaproteobacteria;o__Myxococcales;f__Polyangiaceae;g__Chondromyces	0.0%	0.0%
k__Bacteria;p__Proteobacteria;c__Deltaproteobacteria;o__Myxococcales;f__Polyangiaceae;g__Polyangium	0.0%	0.0%
k__Bacteria;p__Proteobacteria;c__Deltaproteobacteria;o__Myxococcales;f__Polyangiaceae;g__Sorangium	0.0%	0.0%
k__Bacteria;p__Proteobacteria;c__Deltaproteobacteria;o__NB1-j;f__;g__	0.0%	0.0%
k__Bacteria;p__Proteobacteria;c__Deltaproteobacteria;o__NB1-j;f__JTB38;g__	0.0%	0.0%
k__Bacteria;p__Proteobacteria;c__Deltaproteobacteria;o__Spirobacillales;f__;g__	0.0%	0.0%
k__Bacteria;p__Proteobacteria;c__Deltaproteobacteria;o__Sva0853;f__JTB36;g__	0.0%	0.0%
k__Bacteria;p__Proteobacteria;c__Deltaproteobacteria;o__Syntrophobacterales;f__Syntrophaceae;g__Desulfobacca	0.0%	0.0%
k__Bacteria;p__Proteobacteria;c__Deltaproteobacteria;o__Syntrophobacterales;f__Syntrophobacteraceae;g__	0.0%	0.0%
k__Bacteria;p__Proteobacteria;c__Deltaproteobacteria;o__[Entotheonellales];f__;g__	0.0%	0.0%
k__Bacteria;p__Proteobacteria;c__Epsilonproteobacteria;o__Campylobacterales;f__Campylobacteraceae;g__	0.0%	0.0%
k__Bacteria;p__Proteobacteria;c__Epsilonproteobacteria;o__Campylobacterales;f__Campylobacteraceae;g__Arcobacter	0.0%	0.0%
k__Bacteria;p__Proteobacteria;c__Epsilonproteobacteria;o__Campylobacterales;f__Campylobacteraceae;g__Sulfurospirillum	0.0%	0.0%
k__Bacteria;p__Proteobacteria;c__Epsilonproteobacteria;o__Campylobacterales;f__Helicobacteraceae;g__	0.0%	0.0%
k__Bacteria;p__Proteobacteria;c__Epsilonproteobacteria;o__Campylobacterales;f__Helicobacteraceae;g__Sulfuricurvum	0.0%	0.0%
k__Bacteria;p__Proteobacteria;c__Gammaproteobacteria;o__;f__;g__	0.0%	0.0%
k__Bacteria;p__Proteobacteria;c__Gammaproteobacteria;o__34P16;f__;g__	0.0%	0.0%
k__Bacteria;p__Proteobacteria;c__Gammaproteobacteria;o__Aeromonadales;f__;g__	0.0%	0.0%
k__Bacteria;p__Proteobacteria;c__Gammaproteobacteria;o__Aeromonadales;f__Aeromonadaceae;g__	0.1%	0.2%
k__Bacteria;p__Proteobacteria;c__Gammaproteobacteria;o__Aeromonadales;f__Aeromonadaceae;g__Aeromonas	0.0%	0.0%
k__Bacteria;p__Proteobacteria;c__Gammaproteobacteria;o__Aeromonadales;f__Aeromonadaceae;g__Oceanimonas	0.0%	0.0%
k__Bacteria;p__Proteobacteria;c__Gammaproteobacteria;o__Aeromonadales;f__Aeromonadaceae;g__Tolumonas	0.0%	0.0%
k__Bacteria;p__Proteobacteria;c__Gammaproteobacteria;o__Aeromonadales;f__Succinivibrionaceae;g__Succinivibrio	0.0%	0.0%
k__Bacteria;p__Proteobacteria;c__Gammaproteobacteria;o__Alteromonadales;f__;g__	0.0%	0.0%
k__Bacteria;p__Proteobacteria;c__Gammaproteobacteria;o__Alteromonadales;f__125ds10;g__	0.0%	0.0%
k__Bacteria;p__Proteobacteria;c__Gammaproteobacteria;o__Alteromonadales;f__211ds20;g__	0.0%	0.0%
k__Bacteria;p__Proteobacteria;c__Gammaproteobacteria;o__Alteromonadales;f__Alteromonadaceae;g__	0.1%	0.1%
k__Bacteria;p__Proteobacteria;c__Gammaproteobacteria;o__Alteromonadales;f__Alteromonadaceae;g__Cellvibrio	0.2%	0.1%
k__Bacteria;p__Proteobacteria;c__Gammaproteobacteria;o__Alteromonadales;f__Alteromonadaceae;g__Glaciecola	0.0%	0.0%
k__Bacteria;p__Proteobacteria;c__Gammaproteobacteria;o__Alteromonadales;f__Alteromonadaceae;g__Marinobacter	0.0%	0.0%
k__Bacteria;p__Proteobacteria;c__Gammaproteobacteria;o__Alteromonadales;f__Colwelliaceae;g__	0.0%	0.0%
k__Bacteria;p__Proteobacteria;c__Gammaproteobacteria;o__Alteromonadales;f__Colwelliaceae;g__Thalassomonas	0.0%	0.0%
k__Bacteria;p__Proteobacteria;c__Gammaproteobacteria;o__Alteromonadales;f__HTCC2188;g__HTCC	0.0%	0.0%
k__Bacteria;p__Proteobacteria;c__Gammaproteobacteria;o__Alteromonadales;f__Idiomarinaceae;g__Pseudidiomarina	0.0%	0.0%
k__Bacteria;p__Proteobacteria;c__Gammaproteobacteria;o__Alteromonadales;f__Shewanellaceae;g__Shewanella	0.0%	0.1%
k__Bacteria;p__Proteobacteria;c__Gammaproteobacteria;o__Alteromonadales;f__[Chromatiaceae];g__	0.0%	0.0%
k__Bacteria;p__Proteobacteria;c__Gammaproteobacteria;o__Alteromonadales;f__[Chromatiaceae];g__Alishewanella	0.0%	0.0%
k__Bacteria;p__Proteobacteria;c__Gammaproteobacteria;o__Alteromonadales;f__[Chromatiaceae];g__Alkalimonas	0.0%	0.0%
k__Bacteria;p__Proteobacteria;c__Gammaproteobacteria;o__Alteromonadales;f__[Chromatiaceae];g__Rheinheimera	0.2%	0.2%
k__Bacteria;p__Proteobacteria;c__Gammaproteobacteria;o__Cardiobacteriales;f__;g__	0.0%	0.0%
k__Bacteria;p__Proteobacteria;c__Gammaproteobacteria;o__Chromatiales;f__;g__	0.0%	0.0%
k__Bacteria;p__Proteobacteria;c__Gammaproteobacteria;o__Chromatiales;f__Ectothiorhodospiraceae;g__	0.0%	0.1%
k__Bacteria;p__Proteobacteria;c__Gammaproteobacteria;o__Chromatiales;f__Halothiobacillaceae;g__Thiovirga	0.0%	0.0%
k__Bacteria;p__Proteobacteria;c__Gammaproteobacteria;o__Enterobacteriales;f__Enterobacteriaceae;g__	0.0%	0.0%
k__Bacteria;p__Proteobacteria;c__Gammaproteobacteria;o__Enterobacteriales;f__Enterobacteriaceae;g__Citrobacter	0.0%	0.0%

k__Bacteria;p__Proteobacteria;c__Gammaproteobacteria;o__Enterobacteriales;f__Enterobacteriaceae;g__Enterobacter	0.0%	0.0%
k__Bacteria;p__Proteobacteria;c__Gammaproteobacteria;o__Enterobacteriales;f__Enterobacteriaceae;g__Erwinia	0.0%	0.0%
k__Bacteria;p__Proteobacteria;c__Gammaproteobacteria;o__Enterobacteriales;f__Enterobacteriaceae;g__Escherichia	0.0%	0.0%
k__Bacteria;p__Proteobacteria;c__Gammaproteobacteria;o__Enterobacteriales;f__Enterobacteriaceae;g__Klebsiella	0.0%	0.0%
k__Bacteria;p__Proteobacteria;c__Gammaproteobacteria;o__Enterobacteriales;f__Enterobacteriaceae;g__Pantoea	0.0%	0.0%
k__Bacteria;p__Proteobacteria;c__Gammaproteobacteria;o__Enterobacteriales;f__Enterobacteriaceae;g__Salmonella	0.0%	0.0%
k__Bacteria;p__Proteobacteria;c__Gammaproteobacteria;o__Enterobacteriales;f__Enterobacteriaceae;g__Stenotrophomonas	0.0%	0.0%
k__Bacteria;p__Proteobacteria;c__Gammaproteobacteria;o__Enterobacteriales;f__Enterobacteriaceae;g__Trabulsiella	0.0%	0.0%
k__Bacteria;p__Proteobacteria;c__Gammaproteobacteria;o__Enterobacteriales;f__Enterobacteriaceae;g__Xenorhabdus	0.0%	0.0%
k__Bacteria;p__Proteobacteria;c__Gammaproteobacteria;o__Legionellales;f__;g__	0.0%	0.0%
k__Bacteria;p__Proteobacteria;c__Gammaproteobacteria;o__Legionellales;f__Coxiellaceae;g__	0.0%	0.0%
k__Bacteria;p__Proteobacteria;c__Gammaproteobacteria;o__Legionellales;f__Coxiellaceae;g__Aquicella	0.0%	0.0%
k__Bacteria;p__Proteobacteria;c__Gammaproteobacteria;o__Legionellales;f__Legionellaceae;g__	0.0%	0.0%
k__Bacteria;p__Proteobacteria;c__Gammaproteobacteria;o__Legionellales;f__Legionellaceae;g__Legionella	0.0%	0.0%
k__Bacteria;p__Proteobacteria;c__Gammaproteobacteria;o__Methylococcales;f__;g__	0.0%	0.0%
k__Bacteria;p__Proteobacteria;c__Gammaproteobacteria;o__Methylococcales;f__Crenotrichaceae;g__Crenothrix	0.0%	0.0%
k__Bacteria;p__Proteobacteria;c__Gammaproteobacteria;o__Methylococcales;f__Methylococcaceae;g__Methylosarcina	0.0%	0.0%
k__Bacteria;p__Proteobacteria;c__Gammaproteobacteria;o__Oceanospirillales;f__;g__	0.0%	0.0%
k__Bacteria;p__Proteobacteria;c__Gammaproteobacteria;o__Oceanospirillales;f__Alcanivoracaceae;g__Alcanivorax	0.0%	0.0%
k__Bacteria;p__Proteobacteria;c__Gammaproteobacteria;o__Oceanospirillales;f__Endozoicimonaceae;g__	0.0%	0.0%
k__Bacteria;p__Proteobacteria;c__Gammaproteobacteria;o__Oceanospirillales;f__Halomonadaceae;g__	0.0%	0.0%
k__Bacteria;p__Proteobacteria;c__Gammaproteobacteria;o__Oceanospirillales;f__Halomonadaceae;g__Candidatus Portiera	0.0%	0.0%
k__Bacteria;p__Proteobacteria;c__Gammaproteobacteria;o__Oceanospirillales;f__Halomonadaceae;g__Chromohalobacter	0.0%	0.0%
k__Bacteria;p__Proteobacteria;c__Gammaproteobacteria;o__Oceanospirillales;f__Halomonadaceae;g__Haererehalobacter	0.0%	0.0%
k__Bacteria;p__Proteobacteria;c__Gammaproteobacteria;o__Oceanospirillales;f__Halomonadaceae;g__Halomonas	0.0%	0.0%
k__Bacteria;p__Proteobacteria;c__Gammaproteobacteria;o__Oceanospirillales;f__Oceanospirillaceae;g__	0.0%	0.0%
k__Bacteria;p__Proteobacteria;c__Gammaproteobacteria;o__Oceanospirillales;f__Oceanospirillaceae;g__Marinomonas	0.0%	0.0%
k__Bacteria;p__Proteobacteria;c__Gammaproteobacteria;o__Oceanospirillales;f__Saccharospirillaceae;g__Saccharospirillum	0.0%	0.0%
k__Bacteria;p__Proteobacteria;c__Gammaproteobacteria;o__Pasteurellales;f__Pasteurellaceae;g__Aggregatibacter	0.0%	0.0%
k__Bacteria;p__Proteobacteria;c__Gammaproteobacteria;o__Pasteurellales;f__Pasteurellaceae;g__Haemophilus	0.0%	0.0%
k__Bacteria;p__Proteobacteria;c__Gammaproteobacteria;o__Pseudomonadales;f__Moraxellaceae;g__	0.1%	0.2%
k__Bacteria;p__Proteobacteria;c__Gammaproteobacteria;o__Pseudomonadales;f__Moraxellaceae;g__Acinetobacter	0.5%	0.8%
k__Bacteria;p__Proteobacteria;c__Gammaproteobacteria;o__Pseudomonadales;f__Moraxellaceae;g__Enhydrobacter	0.0%	0.0%
k__Bacteria;p__Proteobacteria;c__Gammaproteobacteria;o__Pseudomonadales;f__Moraxellaceae;g__Moraxella	0.0%	0.0%
k__Bacteria;p__Proteobacteria;c__Gammaproteobacteria;o__Pseudomonadales;f__Moraxellaceae;g__Perlucidibaca	0.0%	0.0%
k__Bacteria;p__Proteobacteria;c__Gammaproteobacteria;o__Pseudomonadales;f__Moraxellaceae;g__Psychrobacter	0.0%	0.0%
k__Bacteria;p__Proteobacteria;c__Gammaproteobacteria;o__Pseudomonadales;f__Pseudomonadaceae;g__	0.1%	0.1%
k__Bacteria;p__Proteobacteria;c__Gammaproteobacteria;o__Pseudomonadales;f__Pseudomonadaceae;g__Azorhizophilus	0.0%	0.0%
k__Bacteria;p__Proteobacteria;c__Gammaproteobacteria;o__Pseudomonadales;f__Pseudomonadaceae;g__Pseudomonas	13.7%	19.2%
k__Bacteria;p__Proteobacteria;c__Gammaproteobacteria;o__Pseudomonadales;f__Pseudomonadaceae;g__Serpens	0.0%	0.0%
k__Bacteria;p__Proteobacteria;c__Gammaproteobacteria;o__Thiotrichales;f__Piscirickettsiaceae;g__	0.0%	0.0%
k__Bacteria;p__Proteobacteria;c__Gammaproteobacteria;o__Thiotrichales;f__Piscirickettsiaceae;g__Methylophaga	0.0%	0.0%
k__Bacteria;p__Proteobacteria;c__Gammaproteobacteria;o__Vibrionales;f__Pseudoalteromonadaceae;g__	0.0%	0.0%
k__Bacteria;p__Proteobacteria;c__Gammaproteobacteria;o__Vibrionales;f__Pseudoalteromonadaceae;g__Pseudoalteromonas	0.0%	0.0%
k__Bacteria;p__Proteobacteria;c__Gammaproteobacteria;o__Vibrionales;f__Pseudoalteromonadaceae;g__Vibrio	0.0%	0.0%
k__Bacteria;p__Proteobacteria;c__Gammaproteobacteria;o__Vibrionales;f__Vibrionaceae;g__Photobacterium	0.0%	0.0%
k__Bacteria;p__Proteobacteria;c__Gammaproteobacteria;o__Vibrionales;f__Vibrionaceae;g__Vibrio	0.0%	0.0%
k__Bacteria;p__Proteobacteria;c__Gammaproteobacteria;o__Xanthomonadales;f__Sinobacteraceae;g__	0.0%	0.0%
k__Bacteria;p__Proteobacteria;c__Gammaproteobacteria;o__Xanthomonadales;f__Xanthomonadaceae;g__	0.0%	0.0%
k__Bacteria;p__Proteobacteria;c__Gammaproteobacteria;o__Xanthomonadales;f__Xanthomonadaceae;g__Aquimonas	0.0%	0.0%
k__Bacteria;p__Proteobacteria;c__Gammaproteobacteria;o__Xanthomonadales;f__Xanthomonadaceae;g__Arenimonas	0.0%	0.0%
k__Bacteria;p__Proteobacteria;c__Gammaproteobacteria;o__Xanthomonadales;f__Xanthomonadaceae;g__Aspromonas	0.0%	0.0%
k__Bacteria;p__Proteobacteria;c__Gammaproteobacteria;o__Xanthomonadales;f__Xanthomonadaceae;g__Dokdonella	0.0%	0.0%
k__Bacteria;p__Proteobacteria;c__Gammaproteobacteria;o__Xanthomonadales;f__Xanthomonadaceae;g__Luteimonas	0.0%	0.0%
k__Bacteria;p__Proteobacteria;c__Gammaproteobacteria;o__Xanthomonadales;f__Xanthomonadaceae;g__Lysobacter	0.0%	0.0%
k__Bacteria;p__Proteobacteria;c__Gammaproteobacteria;o__Xanthomonadales;f__Xanthomonadaceae;g__Pseudoxanthomonas	0.0%	0.0%
k__Bacteria;p__Proteobacteria;c__Gammaproteobacteria;o__Xanthomonadales;f__Xanthomonadaceae;g__Rhodanobacter	0.0%	0.0%
k__Bacteria;p__Proteobacteria;c__Gammaproteobacteria;o__Xanthomonadales;f__Xanthomonadaceae;g__Silanimonas	0.0%	0.0%

	k__Bacteria;p__Proteobacteria;c__Gammaproteobacteria;o__Xanthomonadales;f__Xanthomonadaceae;g__Stenotrophomonas	0.0%	0.1%
	k__Bacteria;p__Proteobacteria;c__Gammaproteobacteria;o__Xanthomonadales;f__Xanthomonadaceae;g__Thermomonas	0.0%	0.0%
	k__Bacteria;p__Proteobacteria;c__Gammaproteobacteria;o__Xanthomonadales;f__Xanthomonadaceae;g__Xanthomonas	0.0%	0.0%
	k__Bacteria;p__Proteobacteria;c__Gammaproteobacteria;o__[Marinicellales];f__[Marinicellaceae];g__	0.0%	0.0%
	k__Bacteria;p__SR1;c__;o__;f__;g__	0.0%	0.0%
	k__Bacteria;p__Spirochaetes;c__Spirochaetes;o__Spirochaetales;f__Spirochaetaceae;g__Spirochaeta	0.0%	0.0%
	k__Bacteria;p__Spirochaetes;c__Spirochaetes;o__Spirochaetales;f__Spirochaetaceae;g__Treponema	0.0%	0.0%
	k__Bacteria;p__Spirochaetes;c__[Leptospirae];o__[Leptospirales];f__Leptospiraceae;g__Leptonema	0.0%	0.0%
	k__Bacteria;p__Spirochaetes;c__[Leptospirae];o__[Leptospirales];f__Leptospiraceae;g__Leptospira	0.0%	0.0%
	k__Bacteria;p__Synergistetes;c__Synergistia;o__Synergistales;f__Synergistaceae;g__vadinCA02	0.0%	0.0%
	k__Bacteria;p__TM6;c__SJA-4;o__S1198;f__;g__	0.0%	0.0%
	k__Bacteria;p__TM7;c__;o__;f__;g__	0.0%	0.0%
	k__Bacteria;p__TM7;c__TM7-1;o__;f__;g__	0.0%	0.0%
	k__Bacteria;p__TM7;c__TM7-3;o__;f__;g__	0.0%	0.0%
	k__Bacteria;p__TM7;c__TM7-3;o__CW040;f__;g__	0.0%	0.0%
	k__Bacteria;p__TM7;c__TM7-3;o__EW055;f__;g__	0.0%	0.0%
	k__Bacteria;p__TM7;c__TM7-3;o__I025;f__;g__	0.0%	0.0%
	k__Bacteria;p__Tenericutes;c__Mollicutes;o__Acholeplasmatales;f__Acholeplasmataceae;g__Acholeplasma	0.0%	0.0%
	k__Bacteria;p__Thermotogae;c__MS9;o__;f__;g__	0.0%	0.0%
	k__Bacteria;p__Verrucomicrobia;c__Opitutae;o__;f__;g__	0.0%	0.0%
	k__Bacteria;p__Verrucomicrobia;c__Opitutae;o__Opitutales;f__Opitutaceae;g__	0.0%	0.0%
	k__Bacteria;p__Verrucomicrobia;c__Opitutae;o__Opitutales;f__Opitutaceae;g__Opitutus	0.0%	0.0%
	k__Bacteria;p__Verrucomicrobia;c__Verruco-5;o__WCHB1-41;f__RFP12;g__	0.0%	0.0%
	k__Bacteria;p__Verrucomicrobia;c__Verrucomicrobiae;o__Verrucomicrobiales;f__Verrucomicrobiaceae;g__	0.0%	0.0%
	k__Bacteria;p__Verrucomicrobia;c__Verrucomicrobiae;o__Verrucomicrobiales;f__Verrucomicrobiaceae;g__Luteolibacter	0.0%	0.0%
	k__Bacteria;p__Verrucomicrobia;c__Verrucomicrobiae;o__Verrucomicrobiales;f__Verrucomicrobiaceae;g__Prosthecobacter	0.0%	0.0%
	k__Bacteria;p__Verrucomicrobia;c__Verrucomicrobiae;o__Verrucomicrobiales;f__Verrucomicrobiaceae;g__Verrucomicrobium	0.0%	0.0%
	k__Bacteria;p__Verrucomicrobia;c__[Pedosphaerae];o__[Pedosphaerales];f__;g__	0.0%	0.0%
	k__Bacteria;p__Verrucomicrobia;c__[Pedosphaerae];o__[Pedosphaerales];f__Ellin515;g__	0.0%	0.0%
	k__Bacteria;p__Verrucomicrobia;c__[Pedosphaerae];o__[Pedosphaerales];f__auto67_4W;g__	0.0%	0.0%
	k__Bacteria;p__Verrucomicrobia;c__[Spartobacteria];o__[Chthoniobacterales];f__[Chthoniobacteraceae];g__	0.0%	0.0%
	k__Bacteria;p__Verrucomicrobia;c__[Spartobacteria];o__[Chthoniobacterales];f__[Chthoniobacteraceae];g__Candidatus Xiphinematobacter	0.0%	0.0%
	k__Bacteria;p__WPS-2;c__;o__;f__;g__	0.0%	0.0%
	k__Bacteria;p__ZB3;c__;o__;f__;g__	0.0%	0.0%
	k__Bacteria;p__[Thermi];c__Deinococci;o__Deinococcales;f__Deinococcaceae;g__Deinococcus	0.0%	0.0%
	k__Bacteria;p__[Thermi];c__Deinococci;o__Deinococcales;f__Trueperaceae;g__Truepera	0.0%	0.0%

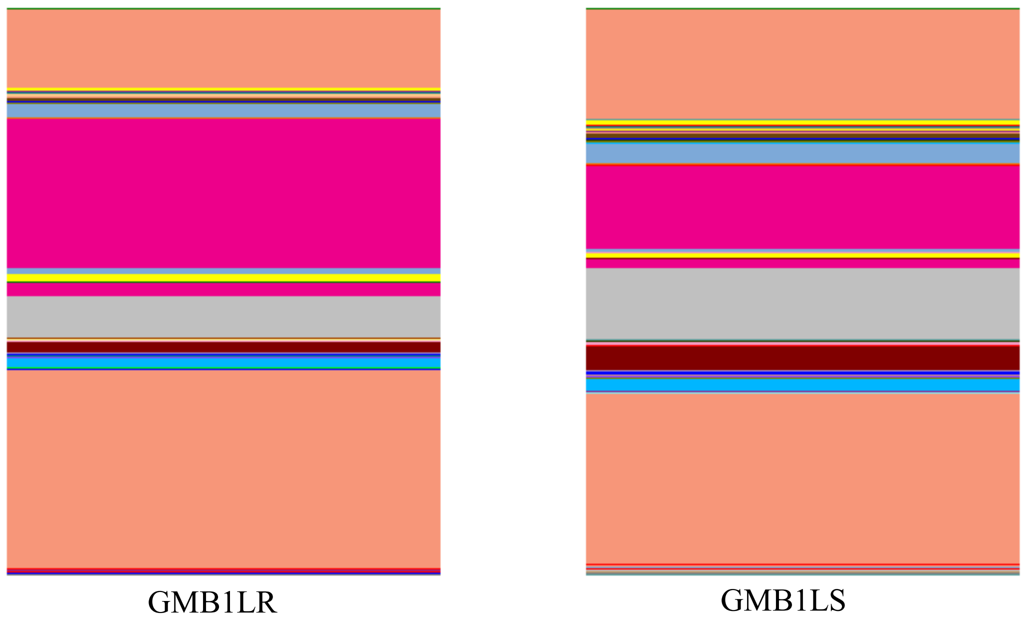

Supplement: Supplementary file 1 — Supplementary Files [file 41598_2017_9791_MOESM1_ESM.pdf]
